# Evaluation of the therapeutic effect of new hypoglycemic drugs on patients with heart failure with reduced ejection fraction and type 2 diabetes: a systematic review and network meta-analysis

**DOI:** 10.3389/fcvm.2026.1799254

**Published:** 2026-05-08

**Authors:** Xin-Rao Chen, Xiao-ping Mao, Dan Luo, Chang-qing Yu, Jia-ning Bai

**Affiliations:** 1 West China Hospital Sichuan University, Meishan Hospital, Meishan, China; 2 Department of General Practice, Meishan People’s Hospital, Meishan, China; 3 Department of Cardiovascular Medicin, Bishan Hospital of Chongqing Medical University, Chongqing, China

**Keywords:** heart failure, hypoglycemic drugs, network meta-analysis, reduced ejection fraction, type 2 diabetes

## Abstract

**Background:**

This systematic review and network meta-analysis synthesizes evidence from randomized controlled trials (RCTs) investigating three novel glucose-lowering therapies in patients with coexisting heart failure with reduced ejection fraction (HFrEF) and type 2 diabetes mellitus(T2DM). We evaluated composite cardiovascular outcomes in this high-risk cohort.

**Methods:**

A comprehensive literature search without language restrictions was performed across PubMed, EMBASE, and the Cochrane Library from inception to September 1, 2025. The network meta-analysis assessed the following endpoints: Composite of cardiovascular death and heart failure hospitalization; Hospitalization for heart failure; Left ventricular ejection fraction (LVEF); N-terminal pro-B-type natriuretic peptide (NT-proBNP); Estimated glomerular filtration rate (eGFR); Glycated hemoglobin (HbA1c). Quality appraisal of included RCTs using the Cochrane Risk of Bias Tool 2.0 (ROB2) and Confidence in Network Meta-Analysis (CINeMA) for grading evidence certainty. The study protocol was prospectively registered with PROSPERO (CRD420251269519).

**Results:**

The final analysis pooled data from 16 randomized trials encompassing 14,710 patients. For the composite of cardiovascular death or hospitalization for heart failure, Sotagliflozin demonstrated the most substantial risk reduction (OR = 0.49; 95% CI 0.39, 0.62). This agent also showed significant efficacy against heart failure readmission (OR = 0.53; 95% CI 0.45, 0.63). Notably, Dapagliflozin improved LVEF more effectively than controls (MD −2.94%; 95% CI −3.89, −1.99). Among patients with HFrEF and T2DM, Empagliflozin significantly reduced NT-proBNP plasma levels vs. placebo (SMD −0.61; 95% CI −0.91, −0.31). Empagliflozin additionally exhibited superior preservation of eGFR (MD −2.08; 95% CI −3.38, −0.78), whereas Vildagliptin achieved the greatest reduction in HbA1c (MD −0.62%; 95% CI −1.12, −0.12).

**Conclusions:**

This meta-analysis provides evidence that Sodium-dependent glucose transporter 2 inhibitors (SGLT2i) may be associated with a reduction in major adverse cardiovascular events (MACE) and exert superior renoprotective effects in patients with HFrEF and comorbid T2DM. Notably, Sotagliflozin demonstrates potentially favorable effects for primary cardiovascular endpoints, whereas Vildagliptin appears to be more effective for HbA1c reduction in this analysis.

**Systematic Review Registration:**

PROSPERO CRD420251269519.

## 1 Introduction

Heart failure (HF) represents a leading cause of global mortality and disability, with an escalating disease burden driven by demographic aging and rising metabolic comorbidities (1–3). Type 2 diabetes mellitus(T2DM) independently elevates incident HF risk and portends higher hospitalization frequency and mortality in established disease. Clinically, HF and T2DM exhibit frequent comorbidity, engaging in bidirectional exacerbation mediated by interconnected pathways such as dysregulated energy metabolism, neurohormonal overactivation, inflammatory cascades, and microvascular dysfunction. This pathophysiological synergy generates a high-risk clinical phenotype marked by accelerated disease progression and diminished long-term survival (4–9). Contemporary HF classification stratifies patients by left ventricular ejection fraction (LVEF) into four distinct categories: heart failure with reduced ejection fraction (HFrEF), heart failure with mildly reduced ejection fraction (HFmrEF), heart failure with preserved ejection fraction (HFpEF) and heart failure with improved ejection fraction (HFimpEF) (10). HFrEF, defined by LVEF ≤40% with concomitant systolic impairment, this distinct clinical entity typically manifests left ventricular dilation, regional wall motion abnormalities, and variable reductions in cardiac output (11). Notably, epidemiological evidence confirms frequent comorbidity between HFrEF and T2DM (8). Cardiac functional impairment and cardiomyocyte injury in diabetes arise from complex interactions among multiple molecular mechanisms (12). Insulin resistance/hyperinsulinemia and impaired glucose tolerance contribute to myocardial dysfunction in patients with T2DM (13), resulting in deleterious effects associated with a number of metabolic abnormalities, such as deposition of advanced glycation end products (AGEs), lipotoxicity and microvascular thinning, and deleterious interactions between these pathophysiological mechanisms may have potentiating effects, leading to several adverse adaptive effects and resulting in myocyte alterations (14). Conversely, HFrEF independently predicts both fatal and non-fatal adverse outcomes in individuals with diabetes (5, 15). Notably, HFrEF may also contribute to the pathogenesis and progression of type 2 diabetes (16).

Accumulating evidence indicates that novel glucose-lowering agents, particularly Sodium-dependent glucose transporter 2 inhibitors (SGLT2i), confer significant cardioprotective benefits beyond glycemic control in HF. By inhibiting renal glucose reabsorption and promoting glycosuria, SGLT2i reduce blood pressure, body weight, and preserve renal function. Consequently, SGLT2i lower cardiovascular mortality, major adverse cardiovascular events (MACE), and HF hospitalization rates while improving key cardiac parameters across the ejection fraction spectrum (17–19). Accumulating clinical evidence suggests that glucagon-like peptide-1 receptor agonists (GLP-1RA) may exert multiorgan benefits beyond glycemic control in specific metabolic disease populations, including potential cardioprotective effects (20–22). GLP-1RA may improves left ventricular function, quality of life and reduces HF hospitalizations, all-cause hospitalizations, and all-cause death (23, 24). However, these benefits must be weighed against emerging safety concerns: large-scale cohort studies report significantly elevated risks of psychiatric disorders (e.g., 195% increase in major depression) among GLP-1RA users (25).In contrast, Dipeptidyl peptidase-4 inhibitors (DPP-4i) demonstrate more modest glycemic effects. DPP-4i, a class of antihyperglycemic agents, demonstrate preclinical efficacy in enhancing perfusion recovery and capillary density in ischemic limbs, while attenuating isoproterenol-induced left ventricular hypertrophy in rodent models (26, 27),While these experimental findings suggest potential cardioprotective mechanisms that may parallel those of SGLT2i, it is important to note that large outcome trials showed neutral effects on major adverse cardiovascular events (28–30). Current evidence regarding glucose-lowering therapies in patients with HF and comorbid T2DM predominantly derives from placebo-controlled or conventional therapy comparisons. Direct head-to-head trials remain scarce. To our knowledge, this is the first Network Meta-Analysis (NMA) specifically designed to rank the relative efficacy of new hypoglycemic drugs—SGLT2i, GLP-1RA, and DPP-4i—on HF-specific endpoints, including cardiovascular death/HF hospitalization and cardiac function (LVEF change and biomarker profiles, within the dedicated HFrEF and T2DM population. To address this gap, we will conduct a systematic review and network meta-analysis of randomized controlled trials. This study aims to quantitatively compare these agents within a unified analytical framework, evaluating their effects on cardiovascular outcomes, cardiac function, and safety in HFrEF-T2DM populations. The findings will provide actionable evidence to support evidence-based clinical decision-making.

## 2 Materials and methods

This network meta-analysis adhered to the PRISMA-NMA guidelines. The study protocol was prospectively registered with PROSPERO (CRD420251269519) to ensure methodological transparency and rigor.

### 2.1 Search strategy

A comprehensive systematic search was conducted across the PubMed, EMBASE, and Cochrane Library databases from their inception to September 1, 2025. The search strategy incorporated both free-text terms and controlled vocabulary (e.g., MeSH in PubMed, Emtree in EMBASE) where applicable. Key search concepts included: “Heart Failure, Systolic”, 'Systolic Heart Failure', “Heart Failure, Reduced Ejection Fraction”, 'Systolic Heart Failure’, “Diabetes Mellitus, Type 2”, “Noninsulin-Dependent Diabetes Mellitus”, “Diabetes Mellitus, Stable”, “Diabetes Mellitus, Noninsulin Dependent”, “Diabetes Mellitus, Adult-Onset”, “Dapagliflozin”, “Vildagliptin”, “Liraglutide”, “Empagliflozin”, 'Sotagliflozin', “Canagliflozin”, and “RCT”. Meanwhile, our systematic search included grey literature sources, clinical trial registries, and conference proceedings. The final literature search was conducted on September 30, 2025, and the manuscript was submitted on January 29, 2026, representing an interval of 4 months. No major publications altering the study conclusions were identified during this period through ongoing surveillance of key sources. The complete search syntax is detailed in Supplementary Table S2. No language restrictions were applied during the search process.

### 2.2 Selection criteria

#### 2.2.1 Inclusion criteria

Inclusion criteria were defined:(1) Patients aged ≥18 years with a confirmed diagnosis of HFrEF comorbid with T2DM were included. HFrEF was defined as New York Heart Association (NYHA) cardiac function class II to IV with LVEF ≤40%. T2DM was defined by a documented history of diabetes diagnosis or meeting diagnostic criteria at baseline;(2) Intervention measures comprised treatment regimens primarily using hypoglycemic drugs as the core strategy. Both monotherapy and combination therapy were permitted, whether utilized as first-line or second-line hypoglycemic treatments,specific drug names and dosing regimens must be clearly stated;(3) Control measures include placebo or conventional therapy (standard heart failure treatments, including ACEi/ARB/ARNI, beta-blockers, MRAs, Diuretic), and glucose-lowering regimens used across the included trials;(4) Only randomized controlled trials were included, encompassing parallel-group designs. For crossover trials, only data from the first phase that was accessible was incorporated;(5) Randomized controlled trials must report at least one of the following outcome measures: The Primary Composite Outcome, defined as a composite of cardiovascular death or hospitalization for heart failure; Hospitalization for Heart Failure(HHF), defined as hospitalization due to heart failure, including first and recurrent events; LVEF is defined as the percentage of blood volume pumped out of the left ventricle relative to its end-diastolic volume during each contraction. NT-proBNP is defined as N-terminal pro-B-type natriuretic peptide, a signal molecule released by cardiac myocytes in response to pressure or volume overload. Estimated glomerular filtration rate (eGFR) is defined as the volume of ultrafiltrate produced by both kidneys per unit time, measured in milliliters per minute. Glycated hemoglobin (HbA1c) is defined as the product formed by the non-enzymatic binding of glucose to hemoglobin within red blood cells, reflecting the average blood glucose levels over the preceding 2–3 months.

#### 2.2.2 Exclusion criteria

Exclusion criteria were included:(1) Studies where required data were unobtainable;(2) Non-randomized controlled trials (including animal studies, protocols, cohort studies, and case reports/letters);(3) Duplicate publications or multiple reports from the same trial: the publication with the most complete data, longest follow-up, or most recent publication date was retained; others were used for supplementary information but not repeatedly included.

Randomized controlled trials (RCTs) were initially screened against predefined inclusion criteria through independent review of titles and abstracts. All retained studies underwent duplicate verification by two investigators to confirm the inclusion of the most current evidence available.

### 2.3 Data extraction and screening process

Two investigators independently extracted trial data in accordance with Preferred Reporting Items for Systematic Reviews and Meta-Analyses (PRISMA) guidelines. Discrepancies were resolved through consensus consultation with a third reviewer. The following variables were systematically recorded from each included study: trial identifiers, authorship, publication year, intervention protocols, and outcome metrics. Primary endpoints included: the composite outcome of cardiovascular death or hospitalization for heart failure, LVEF, NT-proBNP concentrations, eGFR, and HbA1c levels. Studies with incongruent outcome definitions were mapped to consensus criteria (31, 32). Continuous variables reported as Median[Q_1,_Q_3_] were converted to mean ± SD using established formulae $Mean\approx \frac{Q1+2Median+Q3}{4}$, $SD\approx \frac{IQR}{1.35}$, (IQR=Q3−Q1) and unit conversions followed international biological standards. Sensitivity analyses excluding studies requiring data transformation confirmed result robustness. For continuous variables, we prioritized extraction of baseline-to-endpoint mean changes with standard deviations. When only endpoint values were reported, mean differences and dispersion metrics were derived using Cochrane Handbook computational methods. Where within-study correlation coefficients (r) were unreported and could not be extrapolated from existing data, we conservatively assumed r = 0.5 for variance imputation. Sensitivity analyses evaluated the robustness of pooled estimates to these methodological assumptions.

MEANchange=EndpointMean−BaselineMean

$$SDchange=\sqrt{\begin{array}{rl} & \mathrm{Baseline}\;SD^{2}+\mathrm{Endpoint}\;SD^{2}\\ & -2R\cdot \mathrm{Baseline}\;\mathrm{SD}\cdot \mathrm{Endpoint}\;\mathrm{SD} \end{array}}$$

For dichotomous outcomes, extract the number of events and total participants in each group.

### 2.4 Statistical analysis

We conducted the network meta-analysis using Stata MP 17.0. Laboratory biomarkers and left ventricular function parameters were analyzed as continuous variables, with mean differences (MDs) and 95% confidence intervals (95% CIs) quantifying intervention effects from baseline. For outcomes with heterogeneous measurement scales, standardized mean differences (SMDs) were calculated to harmonize effect sizes. Cardiovascular events—treated as binary variables—were summarized as group-level counts and percentages, with odds ratios (ORs) and 95% CIs employed as effect measures. In the primary analysis, effect sizes were pooled using a random-effects model predicated on the consistency framework. Between-study heterogeneity variance *τ*^2^ was quantified via restricted maximum likelihood (REML) estimation. If the network contains a closed-loop structure, the global inconsistency test is employed to assess consistency, while the node-splitting approach is used for local inconsistency testing; *p* < 0.1 is considered indicative of potential inconsistency. Loop inconsistency factors are utilized to evaluate the consistency of closed loops. The inclusion of zero within the 95% for the inconsistency factor (IF) demonstrates that no statistically significant inconsistency exists between direct and indirect evidence. A network plot is generated to visualize the geometric structure of the network. In the network plot, the size of each node is proportional to the total sample size for each treatment, and the width of the connecting lines represents the number of studies providing direct comparisons between the intervention measures. To rank the intervention measures, we applied multiple ranking metrics including the surface under the cumulative ranking curve, the probability of being the best treatment, and the mean rank to enhance the robustness and interpretability of the results. Publication bias and small-study effects were assessed using comparison-adjusted funnel plots (when the number of included studies exceeded 10). Robustness testing was conducted through Leave-One-Out sensitivity analysis, where individual studies were sequentially excluded followed by repeated random-effects consistency model calculations to compare the direction and magnitude of the pooled effect. To quantify the association between study-level covariates and therapeutic efficacy, We based on prior clinical evidence (33) and theoretical relevance to treatment efficacy (34), we pre-specified patient age and follow-up duration as covariates to be included in the univariate network Meta-regression analysis. Variables were not screened via statistical significance in the dataset to avoid data-driven bias. Reporting regression coefficients (*β*) with 95% CI and Wald test-derived *P*-values; Statistical evidence indicating a modifying effect of covariates was considered when *P* < 0.05.

### 2.5 GRADE

This network meta-analysis applied the GRADE framework and integrated the CINeMA approach to evaluate evidence certainty through standardized methodological rigour. The specific procedures were as follows: using randomized controlled trials as the evaluation benchmark, we first conducted a multidimensional risk of bias assessment for individual studies using the RoB 2.0 tool and achieved weighted integration of risk of bias at the comparison level through the CINeMA contribution matrix. For indirect comparisons, potential effect modifiers (including baseline disease severity, intensity of intervention measures, follow-up duration, etc.) were pre-identified based on the exchangeability assumption, and systematic verification of homogeneity between direct and indirect evidence was performed across aspects such as study population, intervention methods, outcome measures, and study setting. For the precision of effect size estimation, the pre-specified minimal clinically important difference served as the criterion to determine whether the 95% CI crossed the null value or the threshold of clinically important effect. Heterogeneity assessment was conducted by comparing the distribution characteristics of confidence intervals and prediction intervals relative to the minimal clinically important difference, combined with comprehensive evaluation using *τ*^2^/I^2^ statistics. When closed evidence loops existed, evidence consistency was assessed using the direct-indirect evidence agreement test methods provided by CINeMA (SIDE/node-splitting and design-by-treatment information). Between-study bias (including reporting bias) was evaluated through systematic searches of clinical trial registries and grey literature, complemented by small-study effect analysis. Each assessment dimension forms judgment conclusions according to the three-tier standard of “No concerns/Some concerns/Major concerns,” and adjusts the evidence quality level accordingly: each instance of 'Some concerns' reduces the level by one grade, while “Major concerns” reduces it by two grades. After cumulative adjustments, the evidence level at the comparative level is classified as High/Moderate/Low/Very low.

### 2.6 Risk of bias assessment

Two researchers independently appraised the risk of bias in the included RCTs using the Cochrane Risk of Bias Tool for Randomized Trials version 2.0 (RoB 2), with disagreements resolved through consensus or third-party adjudication (35). This evaluation tool encompasses the following five bias domains: randomization process, deviations from intended interventions, measurement of the outcome, missing outcome data and selection of the reported result. For each domain, studies are classified into one of three risk categories: low risk, some concerns, or high risk. The overall risk judgment follows a hierarchical decision algorithm: low risk when all domains receive low-risk ratings; some concerns when ≥1 domain is rated “some concerns” (without any high-risk ratings); high risk if any single domain is judged high risk.

## 3 Results

### 3.1 Systematic review and characteristics of the included studies

**Figure 1:**
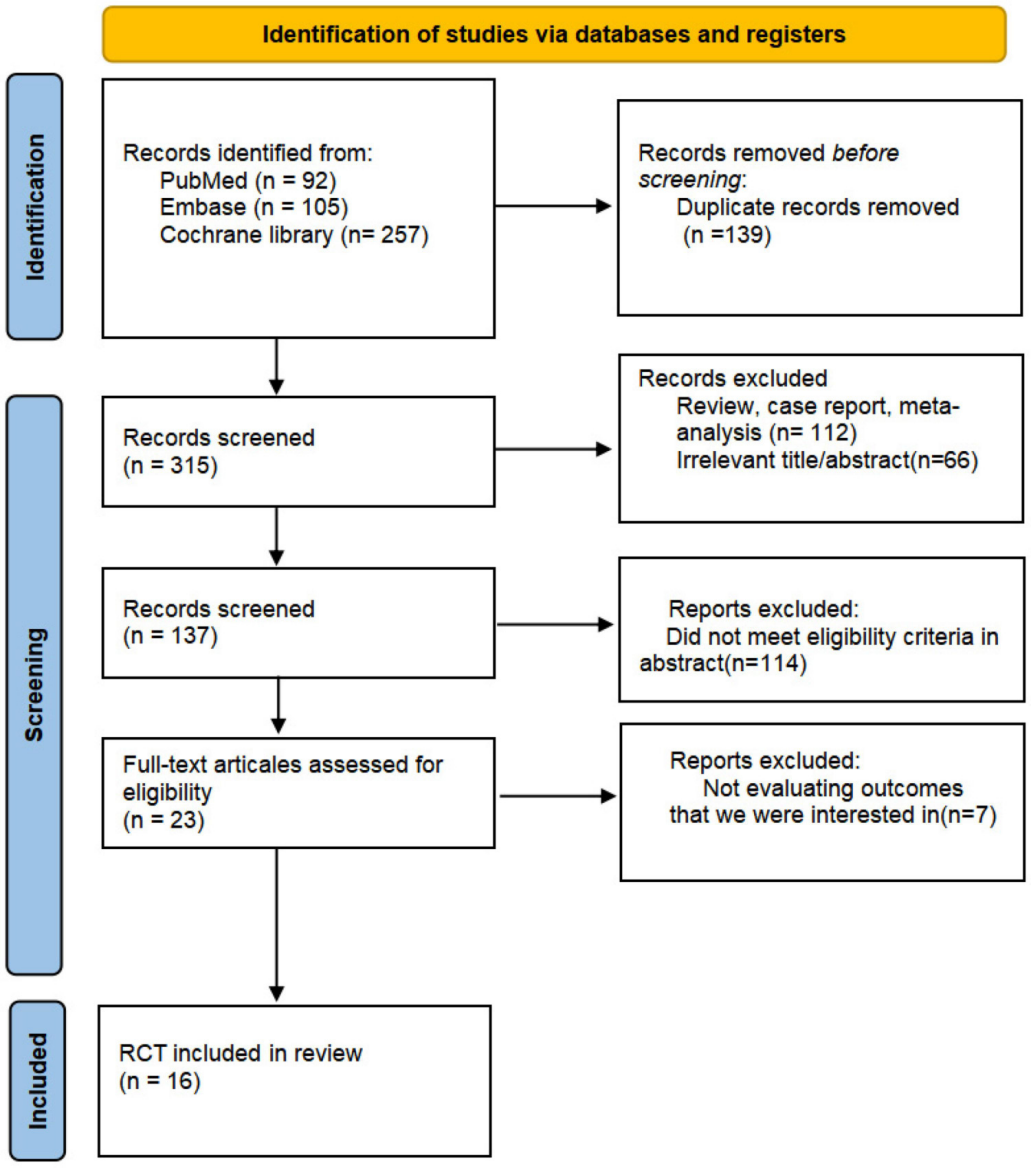
PRISMA flow chart. The flow chart shows the detailed procedures of the study screening and exclusion process.

In the preliminary literature search, we identified 454 records from databases. After excluding duplicates and irrelevant articles through abstract screening, following full-text screening of 23 potentially eligible studies, 16 satisfied the inclusion criteria (Figure 1). These trials enrolled a total of 14,710 participants, involving three classes of drugs (SGLT2i, GLP-1 RA, and DPP4i) (36–50). Among them, there were 13 studies on SGLT2i (including Dapagliflozin, Empagliflozin, Sotagliflozin, and Remogliflozin) (18–50), 2 studies on GLP-1 RA (Albiglutide and Liraglutide) (40, 42) and 1 study on DPP4i (Vildagliptin) (41). The total sample size of the study subjects was moderate, predominantly consisting of middle-aged and elderly patients (with an average age concentrated between 55 and 73 years), and the gender ratio was predominantly male. The post-intervention follow-up duration varied, commonly ranging from 2 to 192 weeks. Among the 14,710 included patients in the study, baseline characteristics were generally balanced across treatment groups, satisfying the exchangeability assumption for indirect comparison. Detailed information for all included studies is presented in Table 1.

**Table 1:** Baseline characteristics of studies included in the network meta-analysis.

First author, year	Registered ID	Sample Size, n	Treatment	Median age	Male/Female	Dosage, mg	Follow-up (weeks)	Background Therapies	Outcomes reported
Drug Type: SGLT2i									
Mats Christian Højbjerg Lassen,2024	NCT03619213	3,150	Dapagliflozin	71	1,818/1,332	10 mg once daily/placebo	32	ACEI/ARB/ARNi, *β*-Blocker, MRA	①
Stefan D. Anker,2021	NCT03057977	1,856	Empagliflozin	66	1,428/428	10 mg once daily/placebo	116	ACEI/ARB/ARNi, Diuretic, β-Blocker, MRA	①②③④⑤⑥
D.L. Bhatt,2020	NCT03521934	1,222	Sotagliflozin	70	810/412	200 mg once daily/placebo	36	ACEI/ARB/ARNi, Diuretic, β-Blocker, MRA	①②③④⑤⑥
Eri Toda Kato,2019	NCT01730534	680	Dapagliflozin	64	571/109	10 mg once daily/placebo	192	ACEI/ARB, Diuretic, β-Blocker, MRA	①②
Mark C. Petrie,2020	NCT03036124	2,139	Dapagliflozin	66	1,662/477	10 mg once daily/placebo	96	ACEI/ARB, Diuretic, β-Blocker, MRA	①②④⑤⑥
A. Eshraghi,2025	IRCT20120215009014N484	73	Empagliflozin	65.5	48/25	10 mg once daily/placebo	12	ACEI/ARB/ARNi, Diuretic, β-Blocker, MRA	③⑥
M. R. Afshani,2024	IRCT20211219053450N1	104	Empagliflozin	55	62/42	10 mg once daily/placebo	24	ACEI/ARB, Diuretic, β-Blocker, MRA, Antiplatelet, Statin	③
Fahmida Ilyas,2021	-	38	Dapagliflozin	73	28/10	10 mg once daily/placebo	2	ACEI/ARB/ARNi, β-Blocker, MRA	③④
Matthew M.Y. Lee,2021	NCT03485092	94	Empagliflozin	68.7	69/25	10 mg once daily/placebo	36	ACEI/ARB/ARNi, β-Blocker, MRA	③④⑤⑥
Qianyu Fu,2023	ChiCTR2300072707	60	Dapagliflozin	70	43/17	10 mg once daily/placebo	48	ACEI/ARB/ARNi, β-Blocker, MRA	③⑥
S. Bhushan,2023	-	35	Remogliflozin	65	20/15	10 mg once daily/placebo	12	ACEI/ARB, Diuretic, β-Blocker, MRA	③⑤
John J.V. McMurray,2023	NCT03877237/NCT03877224	145	Dapagliflozin	69	107/38	10 mg once daily/placebo	16	ACEI/ARB, Diuretic, β-Blocker, MRA, Digitalis glycoside	④
Massimo Iacoviello,2023	-	4,744	Dapagliflozin	66	3,635/1,109	10 mg once daily/placebo	26	ACEI/ARB/ARNi, Diuretic, β-Blocker, MRA, Digitalis glycoside	⑤
Drug Type: GLP-1 RA									
John J. Lepore,2016	NCT01357850	57	Albiglutide	56	41/16	30 mg once weekly/placebo	12	ACEI/ARB, Diuretic, β-Blocker, MRA	③④
Roni Nielsen,2020	NCT01472640	59	Liraglutide	56	53/6	1.8 mg once daily/placebo	24	ACEI/ARB, β-Blocker, Aspirin, Statin	⑤⑥
Drug Type: DPP4i									
John J.V. McMurray,2017	NCT00894868	254	Vildagliptin	63	195/59	50 mg twice daily/placebo	52	ACEI/ARB, Diuretic, β-Blocker, MRA, Digitalis glycoside	①②③⑥

①: the Composite Outcome=the composite of cardiovascular death or hospitalization for heart failure; ②: HHF, Hospitalization for Heart Failure; ③: LVEF, left ventricular ejection fraction; ④: NT-proBNP, N-terminal pro-B-type natriuretic peptide; ⑤: eGFR, estimated glomerular filtration rate; ⑥: HbA1c, Glycated Hemoglobin.

### 3.2 ROB 2.0 assessment

**Figure 2:**
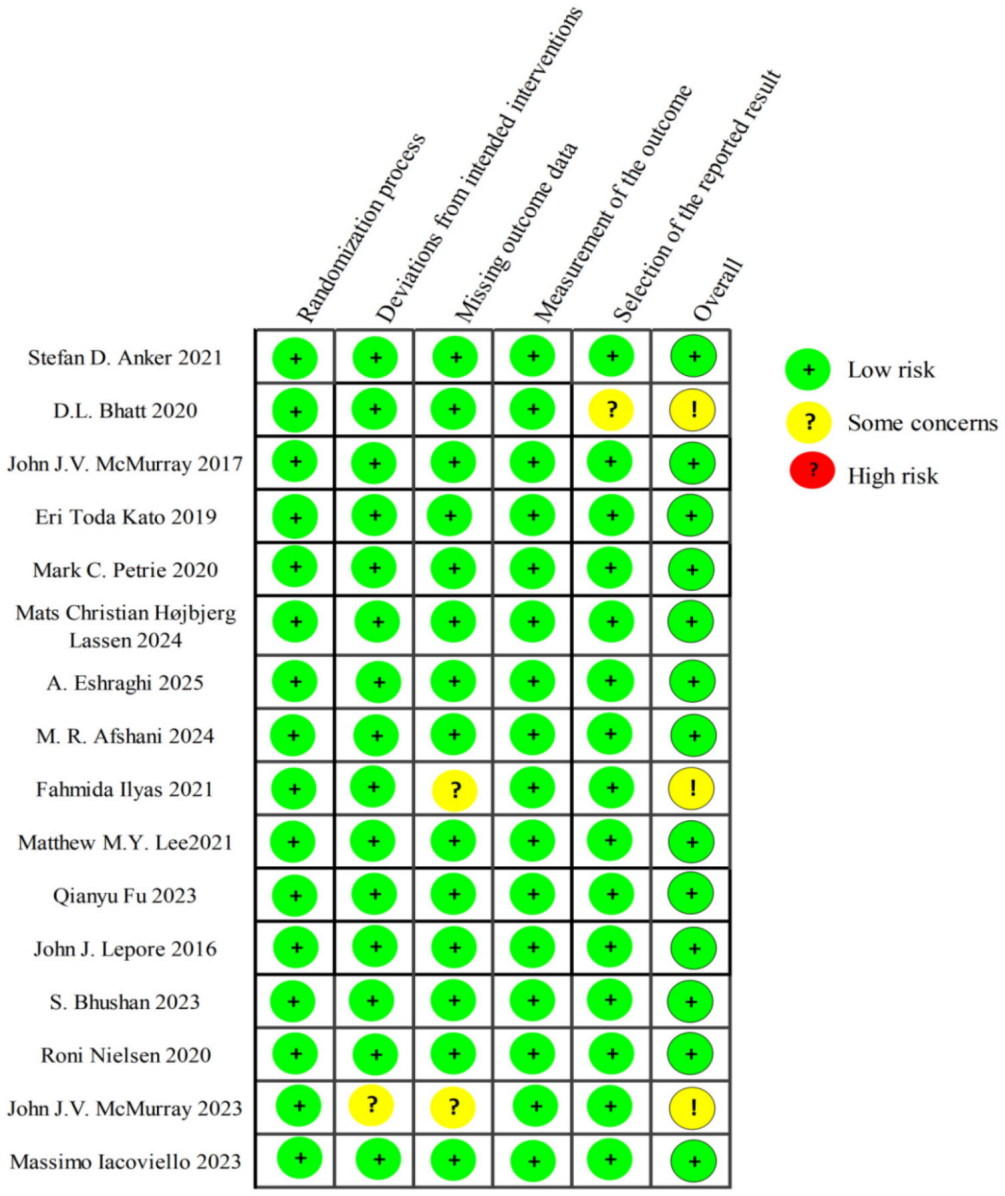
Summary of results from assessment of studies by using cochrane risk of bias tool 2.0.

This study utilized the Cochrane RoB 2.0 tool to assess methodological quality across five predefined bias domains. Of the 16 included studies, 13 demonstrated an overall low risk of bias. Three studies warranted “some concerns” ratings, two of which were primarily attributed to missing outcome data resulting from incomplete baseline documentation. However, the magnitude of missing data was minimal, and the associated parameters were not primary endpoints in this analysis. Consequently, their omission exerts negligible influence on the core findings, yielding an overall low risk of bias. Notably, one trial terminated early due to funding limitations and modified its primary outcome, introducing some concerns regarding bias in the selection of reported results. It should be noted that this endpoint adjustment was completed without knowledge of treatment group assignment, thus expected to have minimal impact on bias from selective outcome reporting. Detailed assessments for each study are presented in Figure 2.

### 3.3 Network meta-analyses

#### 3.3.1 Consistency and inconsistency

**Figure 3:**
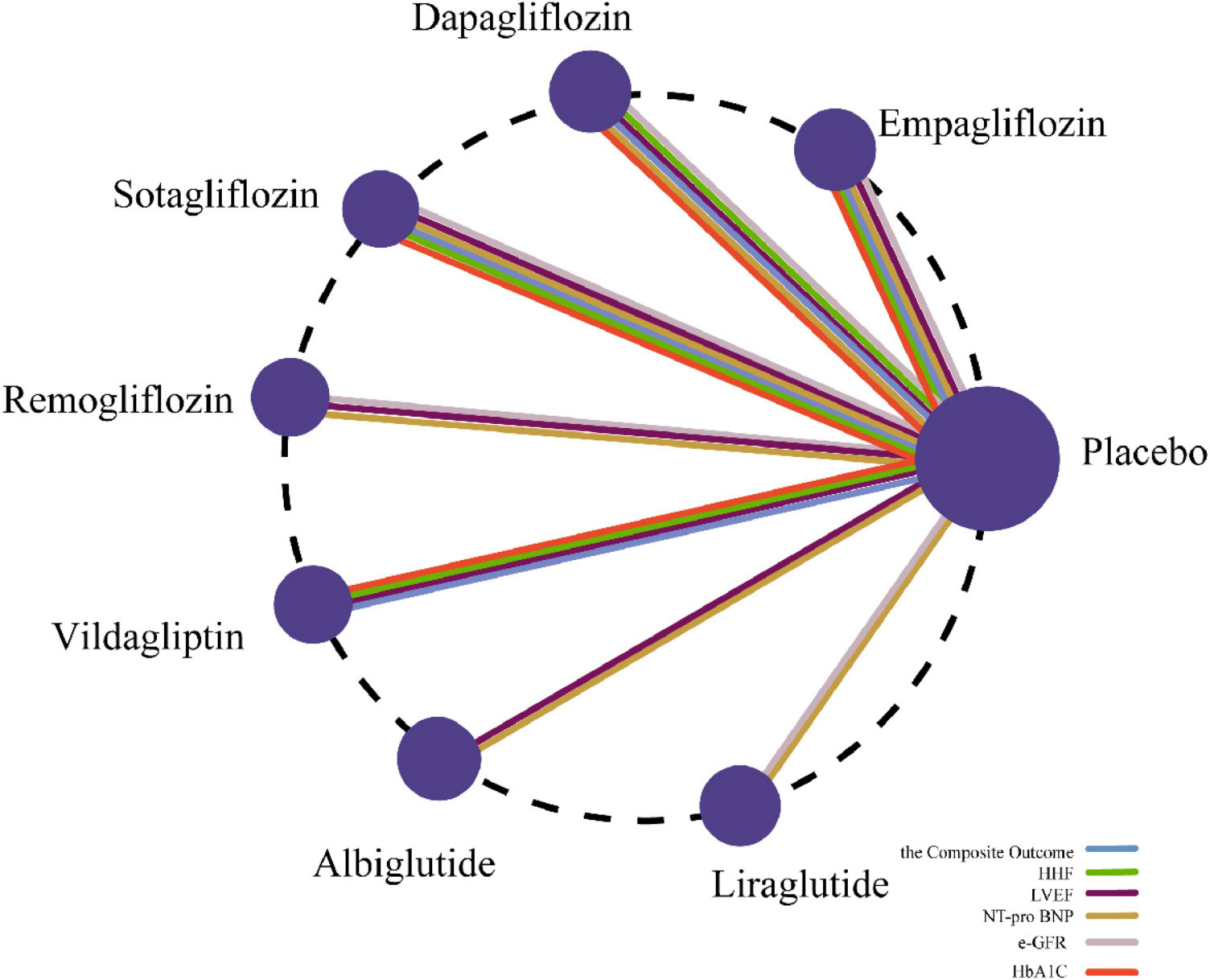
Network plot.

This study designated a composite endpoint of cardiovascular death and heart failure hospitalization, supplemented by first/recurrent heart failure hospitalizations, as primary outcomes. Secondary outcomes comprised LVEF, NT-proBNP, eGFR, and HbA1c. Given that all interventions were exclusively compared against placebo—preventing closed-loop formation in the network—we employed a consistency model for analysis. During evidence quality assessment using GRADE framework, transitivity uncertainties and the absence of closed loops necessitated downgrading for indirectness. Consequently, these findings warrant cautious interpretation. The network plot (Figure 3) was generated to clarify the comparisons involved in the NMA.

#### 3.3.2 Composite outcome of cardiovascular death and hospitalization for heart failure

Network meta-analysis of six studies (*n* = 9,301 patients) evaluating five pharmacologic interventions revealed significantly reduced risk for the composite endpoint of cardiovascular death or heart failure hospitalization in patients with HFrEF and T2DM. Versus placebo, odds ratios were: Sotagliflozin (OR =  0.49, 95% CI: 0.39, 0.62), Empagliflozin (OR =  0.69, 95% CI: 0.56, 0.85), Dapagliflozin (OR =  0.75, 95% CI: 0.66, 0.84), and Vildagliptin (OR =  0.39, 95% CI: 0.18, 0.84).

#### 3.3.3 Hospitalization due to heart failure

**Figure 4:**
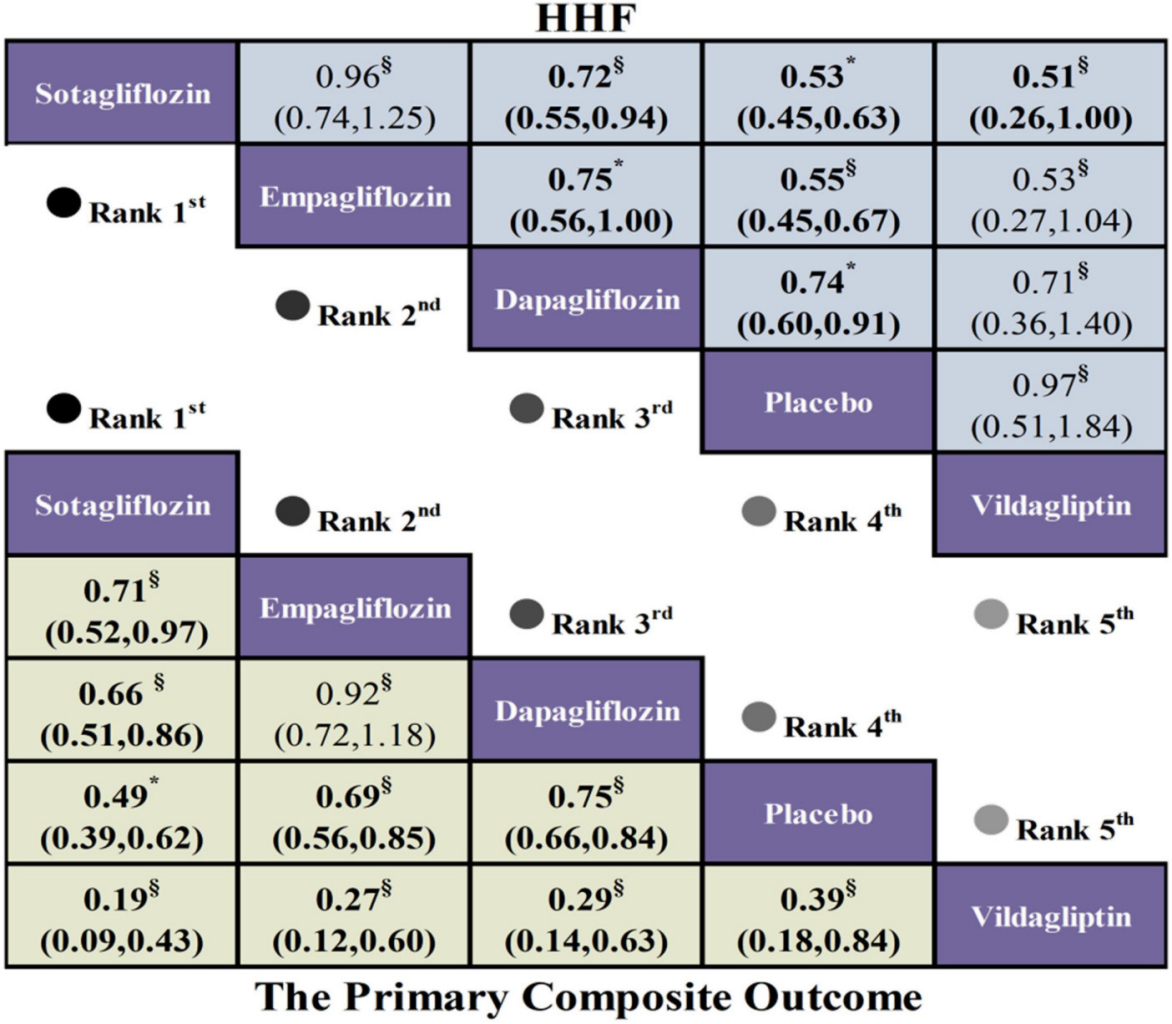
League table comparing therapeutic effects of hypoglycemic drugs on composite outcome of cardiovascular death or hospitalization for heart failure and HHF in HFrEF patients with T2DM (GRADE: Low:§ very Low:* moderate:#).

This meta-analysis evaluated HHF outcomes across five randomized controlled trials, comprising five distinct intervention protocols and 6,151 patients. Relative to placebo, significant reductions in HHF risk were observed with Sotagliflozin (OR =  0.53, 95% CI 0.45, 0.63), Empagliflozin (OR =  0.55, 95% CI 0.45, 0.67), and Dapagliflozin (OR =  0.74, 95% CI 0.60, 0.91). In contrast, Vildagliptin showed no significant difference in HHF risk compared with placebo (OR =  0.97, 95% CI 0.51, 1.84) (Figure 4).

#### 3.3.4 Left ventricular ejection fraction

Analysis of LVEF incorporated data from 10 studies comprising 3,791 patients and 7 treatment regimens. Dapagliflozin (MD=–2.94; 95% CI: −3.89, −1.99) significantly improved LVEF vs. placebo. Direct and indirect comparisons further revealed superior LVEF changes with Dapagliflozin over Vildagliptin (MD=–2.32; 95% CI:–3.64, −1.00), Empagliflozin (MD = –0.25; 95% CI: −3.41, −1.08), and Remogliflozin (MD = –6.18; 95% CI: −10.75, −1.61). Notably, no significant difference emerged between Dapagliflozin and Sotagliflozin (MD = –1.94; 95% CI: −4.01, 0.13).

#### 3.3.5 NT-proBNP plasma levels

**Figure 5:**
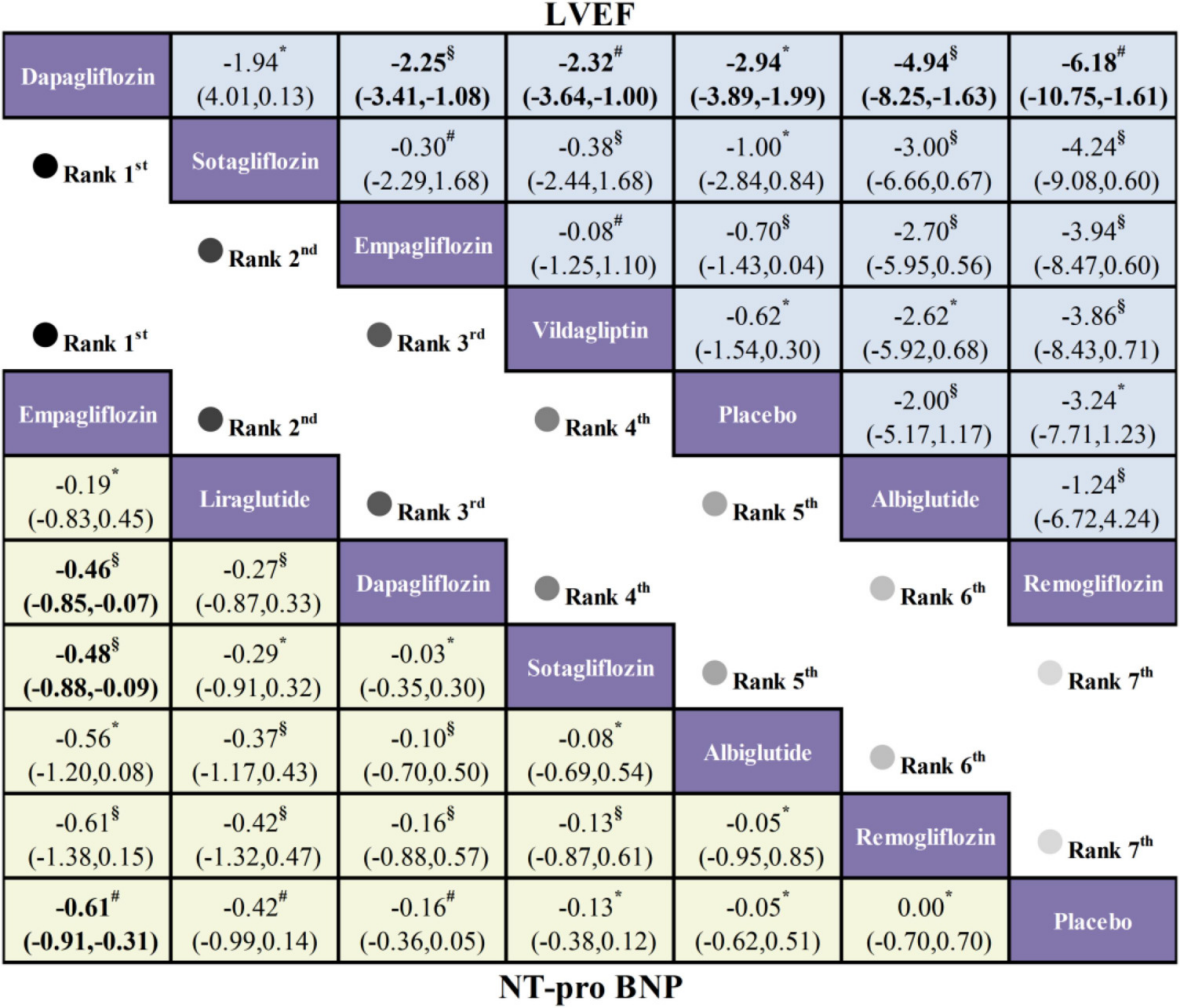
League table comparing therapeutic effects of hypoglycemic drugs on LVEF and NT-proBNP in HFrEF patients with T2DM (GRADE: Low:§ very Low:* moderate:#).

Data on NT-proBNP plasma levels comprised 9 studies encompassing 7 treatment regimens and aggregating 5,645 patients. Empagliflozin demonstrated significant reductions in NT-proBNP vs. placebo (SMD = −0.61, 95% CI: −0.91, −0.31). Statistically superior effects were additionally observed for Empagliflozin relative to both Dapagliflozin (SMD = −0.46, 95% CI: −0.85, −0.07) and Sotagliflozin (SMD = −0.48, 95% CI: −0.88, −0.09), suggesting a comparative efficacy advantage for NT-proBNP reduction (Figure 5).

#### 3.3.6 eGFR

Regarding eGFR, a total of 7 trials involving 10,149 patients and 6 treatment regimens were included. Results showed that compared with placebo, only Empagliflozin (MD = −2.08, 95% CI: −3.38, −0.78) significantly reduced patients' eGFR with statistically significant differences. But this is based on low-certainty evidence due to imprecision and incoherence bias.

#### 3.3.7 Glycated hemoglobin

**Figure 6:**
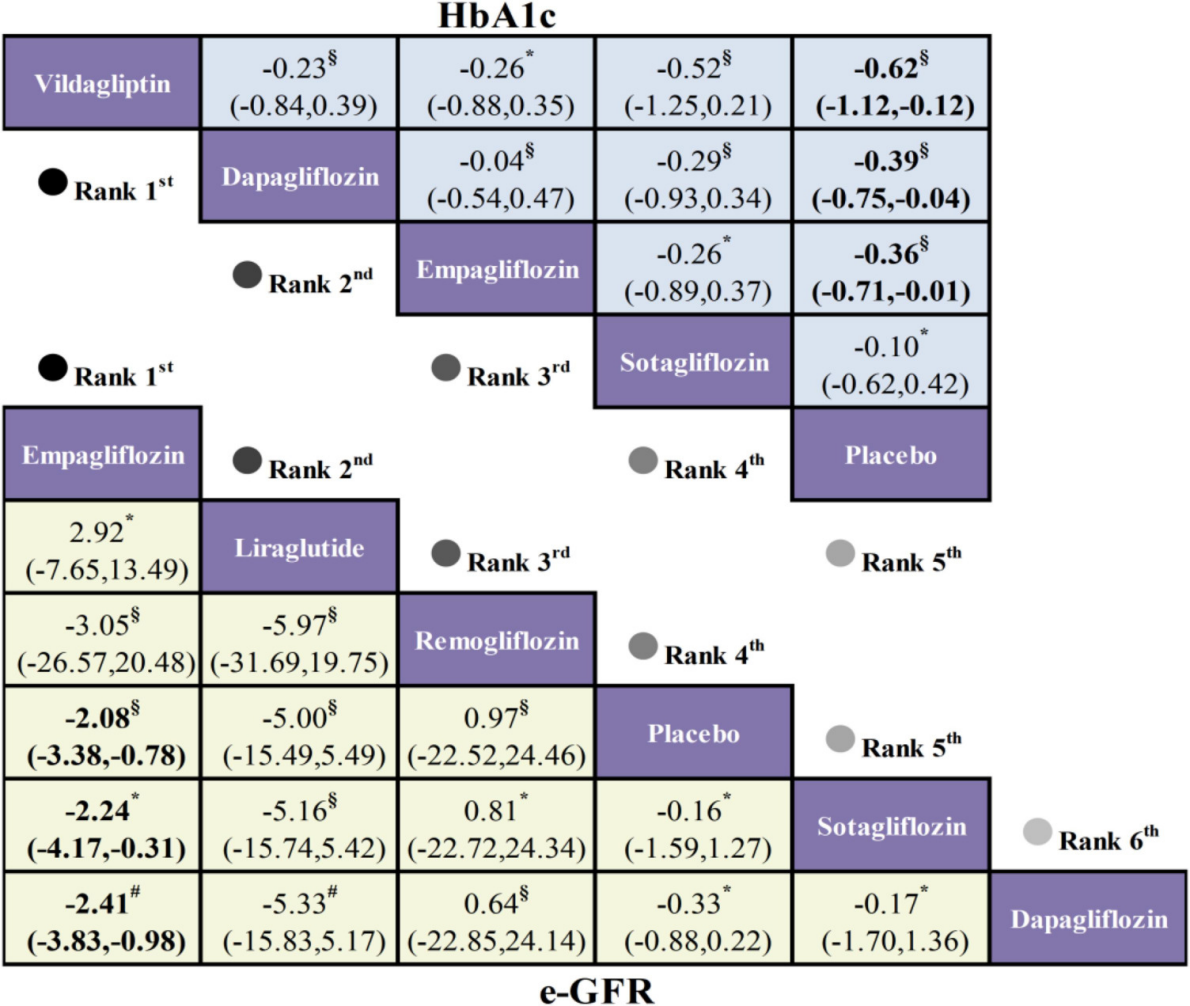
League table comparing the efficacy of different hypoglycemic drugs on HbA1c and eGFR in HFrEF patients with T2DM (GRADE scoring: Low:§ very Low:* moderate:#).

The HbA1c outcomes included 7 studies involving 5,698 patients and 5 treatment regimens. Compared with placebo, Dapagliflozin (MD = −0.39, 95% CI: −0.75, −0.04), Empagliflozin (MD = −0.36, 95% CI: −0.71, −0.01), and Vildagliptin (MD = −0.62, 95% CI: −1.12, −0.12) all demonstrated greater reductions in HbA1c with statistically significant differences. No significant differences were observed in pairwise comparisons between Dapagliflozin, Empagliflozin, Vildagliptin, and Sotagliflozin (Figure 6).

### 3.4 SUCRA values

**Figure 7:**
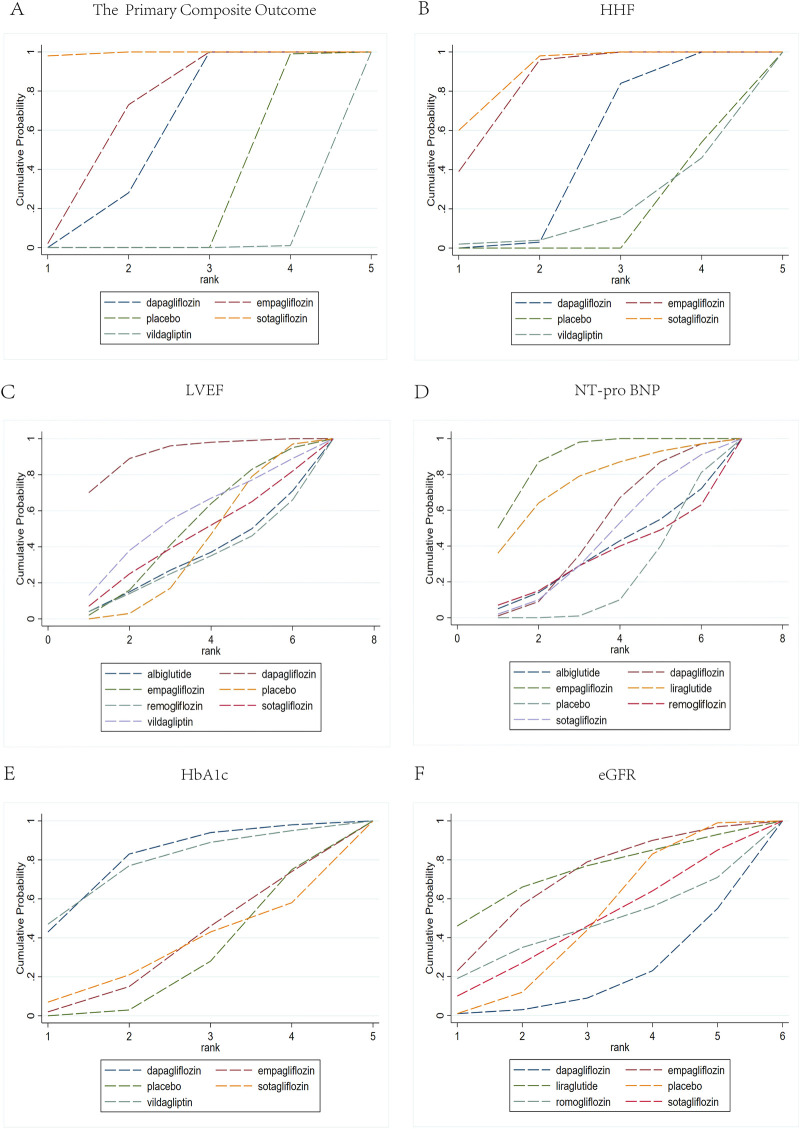
SUCRA graphs compare cumulative probabilities across rank values for various medications. (**A)** shows the primary composite outcome; (**B)** shows HHF (hospitalization for heart failure); (**C)** shows LVEF (left ventricular ejection fraction); (**D)** shows NT-pro BNP (N-terminal pro b-type natriuretic peptide); (**E)** shows HbA1c (hemoglobin A1c); (**F)** shows eGFR (estimated glomerular filtration rate).

The efficacy and safety were ranked based on SUCRA values of each intervention protocol (Figure 7). In patients with HFrEF and concomitant T2DM, Sotagliflozin demonstrated the highest probability of superiority (SUCRA=99.5) for the composite endpoint of cardiovascular death or hospitalization for heart failure(very low certainty due to within-study, heterogeneity and Incoherence bias). Empagliflozin (SUCRA=68.5) and Dapagliflozin (SUCRA=56.9) also conferred measurable protection against this endpoint. For HHF, Sotagliflozin again ranked higher (SUCRA=89.4). Conversely, Dapagliflozin achieved optimal efficacy in improving LVEF (SUCRA=99.4, very low certainty due to within-study, heterogeneity and Incoherence bias). Empagliflozin showed the greater advantage in reducing NT-proBNP and ameliorating heart failure biomarkers (SUCRA=93.3). Regarding renal outcomes, Empagliflozin (SUCRA=77.5) and Liraglutide (SUCRA=77.4) exhibited closely comparable and favorable rankings. Regarding HbA1c reduction and glycemic stability, Vildagliptin (SUCRA=86.8) and Dapagliflozin (SUCRA=65.0) ranked relatively high.

### 3.5 Sensitivity analysis, meta-regression, and publication bias

For the six outcome measures, in the leave-one-out sensitivity analysis, we observed that regardless of which study was excluded, the relative direction of effects remained consistent across the Composite Outcome, HHF, NT-proBNP, and eGFR outcome measures. The absolute magnitude of effects showed minimal variation, with highly overlapping 95% confidence intervals and no substantial changes in statistical significance, indicating robustness of the primary analysis results. However, in the primary analysis comparing dapagliflozin with placebo for the outcome measure of LVEF, a significant advantage was observed. After excluding the study by Fahmida Ilyas (2021), the pooled effect size still indicated that dapagliflozin was superior to placebo, although its 95% confidence interval crossed the zero line. The same was observed for the HbA1c, Dapagliflozin vs. placebo demonstrated a significant advantage in the primary analysis. However, after excluding Mark C. Petrie (2020), the direction of the pooled effect size still indicated that Dapagliflozin was superior to placebo, yet its 95% confidence interval crossed the null line, and statistical significance was no longer maintained. These findings should be interpreted with caution (Supplementary Table S3).

For all outcome measures, we performed univariate network meta-regression to assess whether the follow-up duration and patient age at the study level modified the relative therapeutic effects among different interventions. The regression results showed no significant linear association between either follow-up duration or subject age and the therapeutic effects of any intervention relative to placebo (Supplementary Table S4).

Funnel plots were constructed for all outcome measures to assess potential publication bias. The approximately symmetrical distribution of study estimates, absent discernible outliers, suggests a low risk of publication bias in the present meta-analysis (Supplementary Figure S1-S6).

### 3.6 GRADE assessment

GRADE assessment via CINeMA was applied to all six outcome measures, with quality ratings ranging from high to very low. As all outcome measures in this study featured open-loop structures, some concerns exist regarding the indirectness dimension. For the composite outcome of cardiovascular death and hospitalization for heart failure, 9 out of 10 comparisons received low GRADE ratings, with the remaining one rated very low; In comparisons of HHF outcomes, 7 out of 10 received low-quality ratings, while the remaining three were rated very low. Among 21 pairwise comparisons of LVEF outcomes, merely 4 attained Moderate quality ratings, whereas 11 and 6 were graded as Low and Very Low, respectively. Similarly, for NT-proBNP levels (21 comparisons), only 3 reached Moderate quality; 9 were rated as Low quality, and the remaining 9 were rated as Very Low quality. Glomerular filtration rate outcomes (15 comparisons) fared marginally better, yet still exhibited fragility—Moderate ratings in just 2 cases, contrasted with Low 7 and Very Low 6 designations. For HbA1c, 7 out of 10 comparisons received a Low GRADE rating, while the remaining 3 were rated as Very Low. Detailed ratings are presented in Supplementary Table S5.

## 4 Discussion

This study focuses on the high-risk phenotype of patients with HFrEF comorbid with T2DM. In the absence of sufficient head-to-head randomized evidence, we integrated existing RCT evidence and employed network meta-analysis to compare the effects of different glucose-lowering strategies on cardiovascular outcomes, cardiac function, and metabolic and renal indicators. Overall, this study's findings support that SGLT2i-based regimens demonstrate more consistent advantages in reducing severe HF-related events in this population, with Sotagliflozin showing particularly prominent relative effects on both the primary composite endpoint and HF hospitalization outcomes. In contrast, evidence regarding non-SGLT2 agents for hard endpoints remains relatively limited. Regarding sensitivity analysis, although most outcome measures demonstrate robustness, a small number of outcome measures exhibit consistent effect directions with uncertain statistical significance. Meanwhile, patients with reduced ejection fraction heart failure complicated by T2DM predominantly belong to the elderly population, and cumulative time differences in endpoint events were observed during long-term follow-up. Therefore, meta-regression analysis was conducted using age and follow-up duration as effect modifiers in the included population. Although the regression analysis results showed no significant linear association between follow-up duration or participant age and the therapeutic effects of any intervention relative to placebo, the large span of follow-up time may have influenced the final outcome indicators, particularly the primary outcome. Thus, the interpretation of the results requires caution.

### 4.1 Key findings and clinical interpretation

Regarding primary outcomes (composite of cardiovascular death or hospitalization for heart failure, hospitalization due to heart failure), this study observed Sotagliflozin exhibiting superior relative effects and higher ranking, while Vildagliptin did not demonstrate clear benefits in HF hospitalization-related outcomes. This result aligns with previous meta-analyses focusing on SGLT2 inhibitors (51), demonstrating that for patients with HFrEF comorbid with T2DM, if reducing major adverse cardiovascular events (MACE) is prioritized in clinical decision-making, SGLT2 inhibitors should be considered as a higher-priority therapeutic option. However, due to the very low level of evidence, the outcome should be interpreted with caution. Significantly, in the SOLOIST-WHF trial, sotagliflozin demonstrated a 33% reduction in the composite endpoint of cardiovascular death, hospitalization for heart failure, and urgent heart failure visits vs. placebo—an effect magnitude congruent with the present analysis (50, 52). While studies have characterized cardiometabolic mechanisms primarily through selective SGLT2i, Sotagliflozin functions as a dual SGLT1/2 inhibitor with distinct pharmacological properties. SGLT1i suppresses intestinal absorption of glucose and galactose, whereas SGLT2i enhances urinary glucose excretion via reduced reabsorption in proximal renal tubules. This dual action potentiates lipid-lowering effects and cardiovascular risk reduction beyond monovalent SGLT2i (53). Previous meta-analyses did not include Sotagliflozin in the composite of cardiovascular death or hospitalization for heart failure, nor in the analysis of hospitalization due to heart failure. Therefore, findings not mentioned in these prior meta-analyses (54).

Regarding secondary outcomes including LVEF, NT-proBNP, eGFR, and HbA1c, this study observed that SGLT2i ranked highly in relative effects on LVEF, NT-proBNP, and eGFR indicators. SGLT2 inhibitors are strongly recommended by current guidelines for acute heart failure in diabetic patients, with additional benefits in slowing renal function decline (55). This effect has also been confirmed in previous clinical trials on acute decompensated heart failure and studies examining cardiorenal protective effects of SGLT2i (19, 43), consistent with clinical guideline recommendations (1). Although the precise mechanisms remain incompletely characterized, several hypotheses have emerged regarding SGLT2is' cardioprotective effects. Notably, severe left ventricular remodeling—manifested by increased end-systolic/diastolic volumes and reduced LVEF—represents a pathological hallmark of HFrEF. The magnitude of this remodeling independently correlates with hospitalization frequency and mortality risk (56–58). SGLT2i ameliorates this process through dual cardiomyocyte-directed actions: (1) inhibition of myocardial Na⁺/H⁺ exchange (NHE) reduces intracellular calcium overload, thereby enhancing myocyte stability and attenuation of transforming growth factor-beta (TGF-*β*)1-mediated fibroblast activation reverses maladaptive ventricular remodeling. Collectively, these pathways contribute significantly to SGLT2i's observed reduction in HF-related hospitalizations and mortality among HFrEF patients (18, 59); (2) SGLT2i lowers renal glucose threshold to promote urinary glucose excretion, inducing osmotic diuresis that reduces cardiac preload and afterload, thereby decreasing myocardial oxygen consumption (60); (3) SGLT2 inhibitors enhance myocardial energetic efficiency through coordinated downregulation of the insulin-to-glucagon ratio, preferential utilization of ketone bodies, and stimulation of ATP biosynthesis with improved storage fidelity (59, 61).

Early clinical trials of GLP-1RAs consistently reported cardiovascularly neutral outcomes in high-risk populations (62–64). Recent evidence, however, reveals that liraglutide exerts comparable nephroprotective efficacy to Empagliflozin, particularly concerning glomerular filtration rate preservation. Notably, randomized controlled trials establish that GLP-1RAs significantly Improve glycemic control, blood pressure, and dyslipidemia, attenuate systemic inflammation, enhance microvascular perfusion (65–67). Animal experiments have shown Liraglutide demonstrates renoprotective properties by significantly attenuating mesangial expansion and renal fibrosis, while reducing CD45 + inflammatory cell infiltration (68, 69). Compelling evidence further indicates that GLP-1RAs confer broad-spectrum anti-inflammatory effects, extending to chronic inflammatory conditions including atherosclerosis and diabetic complications beyond the kidney (70). Accumulating evidence demonstrates that GLP-1RAs mediate anti-inflammatory activity in chronic inflammatory pathologies, notably atherosclerosis and diabetes mellitus (71, 72). The pathogenic cascade linking atherogenic risk factors to coronary heart disease culminates in myocardial ischemia, acute infarction, and ultimately fatal heart failure. Consequently, GLP-1 RAs confer cardiovascular protection predominantly through multifactorial modulation of atherosclerosis—directly attenuating plaque progression and indirectly mitigating its risk determinants—thereby reducing MACE and cardiovascular mortality (73–77).Does this result suggest that in clinical practice, early administration of GLP-1 RAs is recommended for patients with coronary atherosclerotic heart disease to control further progression of atherosclerosis and reduce cardiovascular events and mortality.

Regarding the indicator of reducing HbA1c and maintaining glycemic stability, Vildagliptin demonstrates a higher relative efficacy ranking. Its mechanism shares similarities with GLP-1RAs, primarily through DPP-4 inhibitors increasing incretin levels and maintaining their biological activity by selectively inhibiting the inactivation of bioactive peptides. Although DPP-4 inhibitors have advantages in terms of glycemic control safety and convenience, their cardiovascular effects exhibit heterogeneity.Multiple studies have shown that certain DPP4 inhibitors may increase the risk of hospitalization for heart failure (78, 79).Meanwhile, a comprehensive meta-analysis of 40 double-blind, randomized phase III/IV trials with prospective cardiovascular event adjudication revealed no association between GLP-1 RAs and increased MACE risk (58). This agent demonstrated favorable cardiovascular safety profiles in heart failure patients; nevertheless, evidence supporting its cardiovascular benefits remains inconclusive. These observations are consistent with existing data on DPP-4i. Our NMA evaluates the cardiac function effects of novel hypoglycemic agents in patients with heart failure. Additionally, relevant literature reviews (80) indicate that SGLT2i, DPP4i and GLP-1RA may possess broader cardioprotective effects beyond glycemic control, including antiarrhythmic properties. For instance, a study published by Shih-Jie Jhuo et al. in 2022 (81) demonstrated that SGLT2i reduced cardiac arrhythmias modulating ion channels and anti-fibrotic pathways. But these observations are based on external evidence and not directly evaluated in our analysis. Future research may further explore the potential of novel hypoglycemic agents in this regard.

### 4.2 Advantages and limitations

This study represents the first comprehensive evaluation of the integrated therapeutic effects of three popular new hypoglycemic drugs specifically in patients with HFrEF comorbid with T2DM. Methodologically, sensitivity analysis was conducted using the Leave-One-Out approach to ensure the robustness of the literature conclusions, while meta-regression was employed to exclude potential modifying effects of candidate covariates on the therapeutic effect. Additionally, this study included 16 publications and clinical trials, encompassing a total of 14,710 patients, providing valuable insights for clinical application. Although this study yielded numerous valuable findings, the following limitations should be noted. Firstly, the network structure of this study primarily relied on comparisons with placebo, with scarce direct head-to-head evidence. The absence of closed loops made inconsistency assessment challenging, making it likely that the ranking of drugs was influenced by the weighting of individual large-scale trials and variations in study populations. Consequently, the ranking results are more suitable for “proposing a potential hierarchy” rather than substituting for real head-to-head comparisons. Second, some studies experienced premature termination or interrupted follow-up due to COVID-19, limiting the comprehensive assessment of clinical benefits in real-world settings. Meanwhile, the vast majority of studies included in the analysis did not report hazard ratios (HR) for major composite endpoints such as cardiovascular death or hospitalization for heart failure. To standardize data processing and incorporate as many studies as possible, we employed an event-based odds ratio (OR) analysis method, which may have some impact on the accuracy and reliability of the study results. Finally, safety outcomes were inadequately reported in several trials, and the low incidence of clinically significant but rare adverse events resulted in limited detection power for inter-drug safety differences.

## 5 Conclusion

This network meta-analysis compared effects of three novel glucose-lowering therapies on cardiovascular outcomes, cardiac function, and safety in patients with coexisting HFrEF and T2DM. The results indicated that SGLT2i is superior to DPP4i and GLP-1 RAs in reduce MACE and renoprotective effects in patients with HFrEF and comorbid T2DM. Whereas Vildagliptin appears to be more effective for HbA1c reduction in this analysis. Meanwhile, Liraglutide exerts comparable nephroprotective efficacy to Empagliflozin. Although SGLT2i and GLP-1 RAs have demonstrated clear cardiovascular protective effects in diabetic patients with heart failure or atherosclerotic disease, their mechanisms of benefit, target populations, and risk profiles differ. In contrast, while DPP-4i exhibit glycemic safety, their potential risk of heart failure and lack of organ-protective effects warrant clinical vigilance. Future research should focus on exploring precision-tailored drug matching strategies based on patient subtypes.

## Data Availability

The original contributions presented in the study are included in the article/Supplementary Material, further inquiries can be directed to the corresponding author/s.

## References

[1] Borja I James S Agewall S Antunes MJ Bucciarelli-Ducci C Bueno H 2017 ESC guidelines for the management of acute myocardial infarction in patients presenting with ST-segment elevation: the task force for the management of acute myocardial infarction in patients presenting with ST-segment elevation of the European Society of Cardiology (ESC). Eur Heart J. (2017) 39(2):119–77. 10.1093/eurheartj/ehx393 28886621

[2] Williams B Mancia G Spiering W Agabiti Rosei E Azizi M Burnier M [2018 ESC/ESH guidelines for the management of arterial hypertension]. Kardiol Pol. (2019) 77(2):71–159. 10.5603/KP.2019.0018 30816983

[3] Cosentino F Grant PJ Aboyans V Bailey CJ Ceriello A Delgado V 2019 ESC guidelines on diabetes, pre-diabetes, and cardiovascular diseases developed in collaboration with the EASD. Eur Heart J. (2019) 41(2):255–323. 10.1093/eurheartj/ehz486 31497854

[4] Lebedev DA Lyasnikova EA Vasilyeva EY Babenko AY Shlyakhto EV. Type 2 diabetes Mellitus and chronic heart failure with midrange and preserved ejection fraction: a focus on Serum biomarkers of fibrosis. J Diabetes Res. (2020) 2020(0):6976153. 10.1155/2020/6976153 33224989 PMC7669344

[5] Seferović PM Petrie MC Filippatos GS Anker SD Rosano G Bauersachs J Type 2 diabetes mellitus and heart failure: a position statement from the heart failure association of the European Society of Cardiology. Eur J Heart Fail. (2018) 20(5):853–72. 10.1002/ejhf.1170 29520964

[6] Miki T Yuda S Kouzu H Miura T. Diabetic cardiomyopathy: pathophysiology and clinical features. Heart Fail Rev. (2012) 18(2):149–66. 10.1007/s10741-012-9313-3 PMC3593009 22453289

[7] Paulus WJ Tschöpe C. A novel paradigm for heart failure with preserved ejection fraction: comorbidities drive myocardial dysfunction and remodeling through coronary microvascular endothelial inflammation. J Am Coll Cardiol. (2013) 62(4):263–71. 10.1016/j.jacc.2013.02.092 23684677

[8] Kenny HC Abel ED. Heart failure in type 2 diabetes Mellitus. Circ Res. (2019) 124(1):121–41. 10.1161/CIRCRESAHA.118.311371 30605420 PMC6447311

[9] McGavock JM Lingvay I Zib I Tillery T Salas N Unger R Cardiac steatosis in diabetes mellitus: a 1H-magnetic resonance spectroscopy study. Circulation. (2007) 116(10):1170–5. 10.1161/CIRCULATIONAHA.106.645614 17698735

[10] Heidenreich PA Bozkurt B Aguilar D Allen LA Byun JJ Colvin MM 2022 AHA/ACC/HFSA guideline for the management of heart failure: executive summary: a report of the American College of Cardiology/American Heart Association joint committee on clinical practice guidelines. J Am Coll Cardiol. (2022) 79(17):1757–80. 10.1016/j.jacc.2021.12.011 35379504

[11] Zhang Y Zhang J Butler J Yang X Xie P Guo D Contemporary epidemiology, management, and outcomes of patients hospitalized for heart failure in China: results from the China heart failure (China-HF) registry. J Card Fail. (2017) 23(12):868–75. 10.1016/j.cardfail.2017.09.014 29029965

[12] Bugger H Abel ED. Molecular mechanisms of diabetic cardiomyopathy. Diabetologia. (2014) 57(4):660–71. 10.1007/s00125-014-3171-6 24477973 PMC3969857

[13] Poornima IG Parikh P Shannon RP. Diabetic cardiomyopathy: the search for a unifying hypothesis. Circ Res. (2006) 98(5):596–605. 10.1161/01.RES.0000207406.94146.c2 16543510

[14] Seferović PM Paulus WJ. Clinical diabetic cardiomyopathy: a two-faced disease with restrictive and dilated phenotypes. Eur Heart J. (2015) 36(27):1718–27, 1727a–1727c. 10.1093/eurheartj/ehv134 25888006

[15] MacDonald MR Petrie MC Hawkins NM Petrie JR Fisher M McKelvie R Diabetes, left ventricular systolic dysfunction, and chronic heart failure. Eur Heart J. (2008) 29(10):1224–40. 10.1093/eurheartj/ehn156 18424786

[16] Dunlay SM Givertz MM Aguilar D Allen LA Chan M Desai AS Type 2 diabetes Mellitus and heart failure: a scientific statement from the American Heart Association and the heart failure society of America: this statement does not represent an update of the 2017 ACC/AHA/HFSA heart failure guideline update. Circulation. (2019) 140(7):e294–324. 10.1161/CIR.0000000000000691 31167558

[17] Zinman B Wanner C Lachin JM Fitchett D Bluhmki E Hantel S Empagliflozin, cardiovascular outcomes, and mortality in type 2 diabetes. N Engl J Med. (2015) 373(22):2117–28. 10.1056/NEJMoa1504720 26378978

[18] Lee MM Brooksbank KJ Wetherall K Mangion K Roditi G Campbell RT Effect of empagliflozin on left ventricular volumes in patients with type 2 diabetes, or prediabetes, and heart failure with reduced ejection fraction (SUGAR-DM-HF). Circulation. (2020) 143(6):516–25. 10.1161/CIRCULATIONAHA.120.052186 33186500 PMC7864599

[19] McMurray JJ Solomon SD Inzucchi SE Køber L Kosiborod MN Martinez FA Dapagliflozin in patients with heart failure and reduced ejection fraction. N Engl J Med. (2019) 381(21):1995–2008. 10.1056/NEJMoa1911303 31535829

[20] Drucker DJ Nauck MA. The incretin system: glucagon-like peptide-1 receptor agonists and dipeptidyl peptidase-4 inhibitors in type 2 diabetes. Lancet. (2006) 368(9548):1696–705. 10.1016/S0140-6736(06)69705-5 17098089

[21] Ussher JR Drucker DJ. Cardiovascular biology of the incretin system. Endocr Rev. (2012) 33(2):187–215. 10.1210/er.2011-1052 22323472 PMC3528785

[22] Kristensen SL Rørth R Jhund PS Docherty KF Sattar N Preiss D Cardiovascular, mortality, and kidney outcomes with GLP-1 receptor agonists in patients with type 2 diabetes: a systematic review and meta-analysis of cardiovascular outcome trials. Lancet Diabetes Endocrinol. (2019) 7(10):776–85. 10.1016/S2213-8587(19)30249-9 31422062

[23] Sokos GG Nikolaidis LA Mankad S Elahi D Shannon RP. Glucagon-like peptide-1 infusion improves left ventricular ejection fraction and functional status in patients with chronic heart failure. J Card Fail. (2006) 12(9):694–9. 10.1016/j.cardfail.2006.08.211 17174230

[24] Velez M Peterson EL Wells K Swadia T Sabbah HN Keoki Williams L Association of antidiabetic medications targeting the glucagon-like peptide 1 pathway and heart failure events in patients with diabetes. J Card Fail. (2014) 21(1):2–8. 10.1016/j.cardfail.2014.10.012 25451709 PMC4276467

[25] Kornelius E Huang J-Y Lo S-C Huang C-N Yang Y-S. The risk of depression, anxiety, and suicidal behavior in patients with obesity on glucagon like peptide-1 receptor agonist therapy. Sci Rep. (2024) 14(1):24433. 10.1038/s41598-024-75965-2 39424950 PMC11489776

[26] Ishii M Shibata R Kondo K Kambara T Shimizu Y Tanigawa T Vildagliptin stimulates endothelial cell network formation and ischemia-induced revascularization via an endothelial nitric-oxide synthase-dependent mechanism. J Biol Chem. (2014) 289(39):27235–45. 10.1074/jbc.M114.557835 25100725 PMC4175356

[27] Miyoshi T Nakamura K Yoshida M Miura D Oe H Akagi S Effect of vildagliptin, a dipeptidyl peptidase 4 inhibitor, on cardiac hypertrophy induced by chronic beta-adrenergic stimulation in rats. Cardiovasc Diabetol. (2014) 13(0):43. 10.1186/1475-2840-13-43 24521405 PMC3926272

[28] Green JB Angelyn Bethel M Armstrong PW Buse JB Engel SS Garg J Effect of sitagliptin on cardiovascular outcomes in type 2 diabetes. N Engl J Med. (2015) 373(3):232–42. 10.1056/NEJMoa1501352 26052984

[29] Scirica BM Bhatt DL Braunwald E Gabriel Steg P Davidson J Hirshberg B Saxagliptin and cardiovascular outcomes in patients with type 2 diabetes mellitus. N Engl J Med. (2013) 369(14):1317–26. 10.1056/NEJMoa1307684 23992601

[30] White WB Cannon CP Heller SR Nissen SE Bergenstal RM Bakris GL Alogliptin after acute coronary syndrome in patients with type 2 diabetes. N Engl J Med. (2013) 369(14):1327–35. 10.1056/NEJMoa1305889 23992602

[31] Writing Committee Members., ACC/AHA Joint Committee Members. 2022 AHA/ACC/HFSA guideline for the management of heart failure. J Card Fail. (2022) 28(5):e1–e167. 10.1016/j.cardfail.2022.02.010 35378257

[32] Bauersachs J Soltani S. [Guidelines of the ESC 2021 on heart failure]. Herz. (2021) 47(1):12–8. 10.1007/s00059-021-05084-5 34825250

[33] Chow EW Fan Y Wu H Lau ES Yang A Chow E Age-specific associations between blood pressure and cardiovascular disease, kidney disease, and death among individuals with type 2 diabetes: a population-based cohort study. Cardiovasc Diabetol. (2026) 25(1):60. 10.1186/s12933-025-03072-1 41606584 PMC12922390

[34] Galli M Benenati S Laudani C Simeone B Sarto G Ortega-Paz L Cardiovascular effects and tolerability of GLP-1 receptor agonists: a systematic review and meta-analysis of 99,599 patients. J Am Coll Cardiol. (2025) 86(20):1805–19. 10.1016/j.jacc.2025.08.027 40892610

[35] Lyu XZ Sun F Zhan SY. [Risk related to bias assessment: (4) revised cochrane risk of bias tool for cluster-randomized control trials (RoB2.0)]. Zhonghua Liu Xing Bing Xue Za Zhi. (2018) 39(2):240–4. Chinese. 10.3760/cma.j.issn.0254-6450.2018.02.020 29495213

[36] Fu Q Zhou L Fan Y Liu F Fan Y Zhang X Effect of SGLT-2 inhibitor, dapagli0ozin, on left ventricular remodeling in patients with type 2 diabetes and HFrEF. BMC Cardiovasc Disord. (2023) 23(1):544. 10.1186/s12872-023-03591-3 37940879 PMC10633988

[37] Afshani MR Torfi E Akiash N Jahanshahi A Mohamadi A Sherafat O. Effect of empagliflozin on left ventricular volumes in type 2 diabetes or prediabetes heart failure patients with reduced ejection fraction. Acta Cardiol. (2024) 79(4):419–25. 10.1080/00015385.2023.2240130 38511517

[38] Kato ET Silverman MG Mosenzon O Zelniker TA Cahn A Furtado RH Effect of dapagliflozin on heart failure and mortality in type 2 diabetes Mellitus. Circulation. (2019) 139(22):2528–36. 10.1161/CIRCULATIONAHA.119.040130 30882238

[39] Iacoviello M Marini M Gori M Orso F Gonzini L Gorini M DAPA-HF applicability: the point of view of a cardiology setting. Acta Cardiol. (2023) 78(7):840–5. 10.1080/00015385.2023.2243130 37605991

[40] Lepore JJ Olson E Demopoulos L Haws T Fang Z Barbour AM Effects of the novel long-acting GLP-1 agonist, albiglutide, on cardiac function, cardiac metabolism, and exercise capacity in patients with chronic heart failure and reduced ejection fraction. JACC Heart Fail. (2016) 4(7):559–66. 10.1016/j.jchf.2016.01.008 27039125

[41] McMurray JJ Ponikowski P Bolli GB Lukashevich V Kozlovski P Kothny W Effects of vildagliptin on ventricular function in patients with type 2 diabetes Mellitus and heart failure: a randomized placebo-controlled trial. JACC Heart Fail. (2017) 6(1):8–17. 10.1016/j.jchf.2017.08.004 29032139

[42] Nielsen R Jorsal A Tougaard RS Rasmussen JJ Schou M Videbaek L The impact of the glucagon-like peptide-1 receptor agonist liraglutide on natriuretic peptides in heart failure patients with reduced ejection fraction with and without type 2 diabetes. Diabetes Obes Metab. (2020) 22(11):2141–50. 10.1111/dom.14135 32627271

[43] Anker SD Butler J Filippatos G Khan MS Marx N Lam CS Effect of empagliflozin on cardiovascular and renal outcomes in patients with heart failure by baseline diabetes Status: results from the EMPEROR-reduced trial. Circulation. (2020) 143(4):337–49. 10.1161/CIRCULATIONAHA.120.051824 33175585 PMC7834911

[44] McMurray JJ Docherty KF de Boer RA Hammarstedt A Kitzman DW Kosiborod MN Effect of dapagliflozin versus placebo on symptoms and 6-Minute walk distance in patients with heart failure: the DETERMINE randomized clinical trials. Circulation. (2023) 149(11):825–38. 10.1161/CIRCULATIONAHA.123.065061 38059368

[45] Ilyas F Jones L Tee SL Horsfall M Swan A Wollaston F Acute pleiotropic effects of dapagliflozin in type 2 diabetic patients with heart failure with reduced ejection fraction: a crossover trial. ESC Heart Fail. (2021) 8(5):4346–52. 10.1002/ehf2.13553 34382353 PMC8497349

[46] Petrie MC Verma S Docherty KF Inzucchi SE Anand I Belohlávek J Effect of dapagliflozin on worsening heart failure and cardiovascular death in patients with heart failure with and without diabetes. JAMA. (2020) 323(14):1353–68. 10.1001/jama.2020.1906 32219386 PMC7157181

[47] Lassen MCH Ostrominski JW Inzucchi SE Claggett BL Kulac I Jhund P Effect of dapagliflozin in patients with diabetes and heart failure with mildly reduced or preserved ejection fraction according to background glucose-lowering therapy: a pre-specified analysis of the DELIVER trial. Eur J Heart Fail. (2024) 26(7):1539–48. 10.1002/ejhf.3269 38745498

[48] Eshraghi A Khalesi S Amini K Salleh FH Sharifikia M Hajmiri MS Empagliflozin ameliorates the oxidative stress profile in type 2 diabetic patients with heart failure and reduced ejection fraction: results of a randomized, double-blind, placebo-controlled study. Rev Recent Clin Trials. (2025) 20(2):167–79. 10.2174/0115748871323540241212060946 39779555

[49] Bhushan S Mardikar H Deshpande N Jayagopal PB Mane A Gadkari R Open-label, randomized, multi-centre study to evaluate safety and efficacy of remogliflozin in patients with type 2 diabetes mellitus with acute decompensated heart failure (HF with reduced ejection fraction, HFrEF). (remo safe-AHF study)[J]. Indian Heart J. (2023) 75:S51. 10.1016/j.ihj.2023.11.109

[50] Bhatt DL Szarek M Steg PG Cannon CP Leiter LA McGuire DK Sotagliflozin in patients with diabetes and recent worsening heart failure. N Engl J Med. (2020) 384(2):117–28. 10.1056/NEJMoa2030183 33200892

[51] McGuire DK Shih WJ Cosentino F Charbonnel B Cherney DZ Dagogo-Jack S Association of SGLT2 inhibitors with cardiovascular and kidney outcomes in patients with type 2 diabetes: a meta-analysis. JAMA Cardiol. (2020) 6(2):148–58. 10.1001/jamacardio.2020.4511 PMC7542529 33031522

[52] Verma S Anker SD Butler J Bhatt DL. Early initiation of SGLT2 inhibitors is important, irrespective of ejection fraction: sOLOIST-WHF in perspective. ESC Heart Failure. (2020) 7(6):3261–7. 10.1002/ehf2.13148

[53] Cariou B Charbonnel B. Sotagliflozin as a potential treatment for type 2 diabetes mellitus. Expert Opin Investig Drugs. (2015) 24(12):1647–56. 10.1517/13543784.2015.1100361 26548423

[54] Song R Liu F Shi X Sun S Chen J Gao H. Effects of new hypoglycemic drugs on patients with heart failure: a systematic review and network meta-analysis. Postgrad Med J. (2024) 101(1194):330–50. 10.1093/postmj/qgae148 39487697

[55] O'Meara E McDonald M Chan M Ducharme A Ezekowitz JA Giannetti N CCS/CHFS heart failure guidelines: clinical trial update on functional mitral regurgitation, SGLT2 inhibitors, ARNI in HFpEF, and tafamidis in amyloidosis. Can J Cardiol. (2020) 36(2):159–69. 10.1016/j.cjca.2019.11.036 32036861

[56] Konstam MA Kramer DG Patel AR Maron MS Udelson JE. Left ventricular remodeling in heart failure: current concepts in clinical significance and assessment. JACC Cardiovasc Imaging. (2011) 4(1):98–108. 10.1016/j.jcmg.2010.10.008 21232712

[57] Kramer DG Trikalinos TA Kent DM Antonopoulos GV Konstam MA Udelson JE. Quantitative evaluation of drug or device effects on ventricular remodeling as predictors of therapeutic effects on mortality in patients with heart failure and reduced ejection fraction: a meta-analytic approach. J Am Coll Cardiol. (2010) 56(5):392–406. 10.1016/j.jacc.2010.05.011 20650361 PMC4523221

[58] Cohn JN Ferrari R Sharpe N. Cardiac remodeling–concepts and clinical implications: a consensus paper from an international forum on cardiac remodeling. Behalf of an international forum on cardiac remodeling. J Am Coll Cardiol. (2000) 35(3):569–82. 10.1016/s0735-1097(99)00630-0 10716457

[59] Baartscheer A Hardziyenka M Schumacher CA Belterman CNW van Borren MMGJ Verkerk AO Chronic inhibition of the na+/H+—exchanger causes regression of hypertrophy, heart failure, and ionic and electrophysiological remodelling. Br J Pharmacol. (2008) 154(6):1266–75. 10.1038/bjp.2008.189 18493245 PMC2483399

[60] Vallon V Thomson SC. Targeting renal glucose reabsorption to treat hyperglycaemia: the pleiotropic effects of SGLT2 inhibition. Diabetologia. (2016) 60(2):215–25. 10.1007/s00125-016-4157-3 27878313 PMC5884445

[61] Karmazyn M. NHE-1: still a viable therapeutic target. J Mol Cell Cardiol. (2013) 61(0):77–82. 10.1016/j.yjmcc.2013.02.006 23429008

[62] Pfeffer MA Claggett B Diaz R Dickstein K Gerstein HC Køber LV Lixisenatide in patients with type 2 diabetes and acute coronary syndrome. N Engl J Med. (2015) 373(23):2247–57. 10.1056/NEJMoa1509225 26630143

[63] Holman RR Bethel MA Mentz RJ Thompson VP Lokhnygina Y Buse JB Effects of once-weekly exenatide on cardiovascular outcomes in type 2 diabetes. N Engl J Med. (2017) 377(13):1228–39. 10.1056/NEJMoa1612917 28910237 PMC9792409

[64] Jorsal A Kistorp C Holmager P Tougaard RS Nielsen R Hänselmann A Effect of liraglutide, a glucagon-like peptide-1 analogue, on left ventricular function in stable chronic heart failure patients with and without diabetes (LIVE)-a multicentre, double-blind, randomised, placebo-controlled trial. Eur J Heart Fail. (2016) 19(1):69–77. 10.1002/ejhf.657 27790809

[65] Lingvay I Catarig A-M Frias JP Kumar H Lausvig NL le Roux CW Efficacy and safety of once-weekly semaglutide versus daily canagliflozin as add-on to metformin in patients with type 2 diabetes (SUSTAIN 8): a double-blind, phase 3b, randomised controlled trial. Lancet Diabetes Endocrinol. (2019) 7(11):834–44. 10.1016/S2213-8587(19)30311-0 31540867

[66] Zinman B Bhosekar V Busch R Holst I Ludvik B Thielke D Semaglutide once weekly as add-on to SGLT-2 inhibitor therapy in type 2 diabetes (SUSTAIN 9): a randomised, placebo-controlled trial. Lancet Diabetes Endocrinol. (2019) 7(5):356–67. 10.1016/S2213-8587(19)30066-X 30833170

[67] Capehorn MS Catarig A-M Furberg JK Janez A Price HC Tadayon S Efficacy and safety of once-weekly semaglutide 1.0 mg vs once-daily liraglutide 1.2 mg as add-on to 1–3 oral antidiabetic drugs in subjects with type 2 diabetes (sustain 10). Diabetes Metab. (2019) 46(2):100–9. 10.1016/j.diabet.2019.101117 31539622

[68] Pin C Xiaozhi S Xiangjin X Yiyang L Zhulin S Rongdan W Liraglutide ameliorates early renal injury by the activation of renal Foxo1 in a type 2 diabetic kidney disease rat model. Diabetes Res Clin Pract. (2018) 137(0):173–82. 10.1016/j.diabres.2017.09.006 29355652

[69] Mima A Hiraoka-Yamomoto J Li Q Kitada M Li C Geraldes P Protective effects of GLP-1 on glomerular endothelium and its inhibition by PKC*β* activation in diabetes. Diabetes. (2012) 61(11):2967–79. 10.2337/db11-1824 22826029 PMC3478518

[70] Maria EO Frederikke ES Henrik EJ Charles P Lotte BK Peter HK. Liraglutide improves the kidney function in a murine model of chronic kidney disease. Nephron. (2020) 144(11):595–606. 10.1159/000509418 32877912

[71] Rakipovski G Rolin B Nøhr J Klewe I Frederiksen KS Augustin R The GLP-1 analogs liraglutide and semaglutide reduce atherosclerosis in ApoE(-/-) and LDLr(-/-) mice by a mechanism that includes inflammatory pathways. JACC Basic Transl Sci. (2019) 3(6):844–57. 10.1016/j.jacbts.2018.09.004 PMC6314963 30623143

[72] Bisgaard LS Bosteen MH Fink LN Sørensen CM Rosendahl A Mogensen CK Liraglutide reduces both atherosclerosis and kidney inflammation in moderately uremic LDLr-/- mice. PLoS One. (2016) 11(12):e0168396. 10.1371/journal.pone.0168396 27992511 PMC5161477

[73] Rossella M Viviana C Stefano R Massimo F. Glp-1ras and cardiovascular disease: is the endothelium a relevant platform? Acta Diabetol. (2023) 60(11):1441–8. 10.1007/s00592-023-02124-w 37401947 PMC10520195

[74] Park B Bakbak E Teoh H Krishnaraj A Dennis F Quan A GLP-1 receptor agonists and atherosclerosis protection: the vascular endothelium takes center stage. Am J Physiol Heart Circ Physiol. (2024) 326(5):H1159–76. 10.1152/ajpheart.00574.2023 38426865

[75] Bruen R Curley S Kajani S Crean D O'Reilly ME Lucitt MB Liraglutide dictates macrophage phenotype in apolipoprotein E null mice during early atherosclerosis. Cardiovasc Diabetol. (2017) 16(1):143. 10.1186/s12933-017-0626-3 29110715 PMC5674826

[76] Rakipovski G Rolin B Nøhr J Klewe I Frederiksen KS Augustin R The GLP-1 analogs liraglutide and semaglutide reduce atherosclerosis in ApoE-/- and LDLr-/- mice by a mechanism that includes in0ammatory pathways. JACC Basic Transl Sci. (2018) 3(6):844–57. 10.1016/j.jacbts.2018.09.004 30623143 PMC6314963

[77] Drucker DJ. The cardiovascular biology of glucagon-like peptide-1. Cell Metab. (2016) 24(1):15–30. 10.1016/j.cmet.2016.06.009 27345422

[78] Spinar J Smahelová A. SAVOR-TIMI 53 - vysledky saxagliptinu a kardiovaskularni vysledky u pacientu s diabetes mellitus 2. Typu [SAVORTIMI 53 - saxagliptin and cardiovascular outcomes in patients with type 2 diabetes mellitus]. Vnitr Lek. (2013) 59(11):1003–7. Czech. 24279445

[79] Iqbal N Parker A Frederich R Donovan M Hirshberg B. Assessment of the cardiovascular safety of saxagliptin in patients with type 2 diabetes mellitus: pooled analysis of 20 clinical trials. Cardiovasc Diabetol. (2014) 13(0):33. 10.1186/1475-2840-13-3 24490835 PMC3918110

[80] Cristina T Mariana T Catalina G-O Ahmed A-A Simona-Alina A-A Florica VM Associations between oral glucose-lowering agents and increased risk for lifethreateningarrhythmias in patients with type 2 diabetes Mellitus-a literaturereview. Medicina (Kaunas). (2023) 59(10):176–73. 10.3390/medicina59101760 37893478 PMC10608201

[81] Shih-Jie J Tsung-Hsien L Yi-Hsiung L Wei-Chung T I-Hsin L Bin-Nan W Clinical observation of Sglt2 inhibitor therapy for cardiac arrhythmia andrelated cardiovascular disease in diabetic patients with controlled hypertension. J Pers Med. (2022) 12(2):271–84. 10.3390/jpm12020271 35207759 PMC8880188

