# Supplementary material for: Evaluation of the therapeutic effect of new hypoglycemic drugs on patients with heart failure with reduced ejection fraction and type 2 diabetes: a systematic review and network meta-analysis
Source: Front Cardiovasc Med. 2026 May 8;13:1799254. doi: 10.3389/fcvm.2026.1799254 (PMC13194605; doi:10.3389/fcvm.2026.1799254)
Supplement: Supplementary file 1 [file Datasheet1.docx]

Supplementary Material

Evaluation of the therapeutic effect of new hypoglycemic drugs on patients with heart failure with reduced ejection fraction and type 2 diabetes: a systematic review and network meta-analysis.

Table S1 PRISMA NMA Checklist of Items to Include When Reporting a Systematic Review Involving a Network Meta-analysis

| **Section/Topic** | **Item #** | **Checklist Item** | **Reported on Page #** |
| --- | --- | --- | --- |
| **TITLE** |  |  |  |
| Title | 1 | Identify the report as a systematic review *incorporating*  *anetwork meta-analysis (or related form of meta-analysis).* | 1 |
|  |  |  |  |
| **ABSTRACT** |  |  |  |
| Structured summary | 2 | Provide a structured summary including, as applicable:  **Background:** main objectives  **Methods:** data sources; study eligibility criteria, participants, and interventions; study appraisal; and *synthesis methods, such as network meta-analysis.*  **Results:** number of studies and participants identified; summary estimates with corresponding confidence/credible intervals; *treatment rankings may also be discussed. Authors may choose to summarize pairwise comparisons against a chosen treatment included in their analyses for brevity.*  **Discussion/Conclusions:** limitations; conclusions and implications of findings. | 1-2 |
|  |  |  |  |
| **INTRODUCTION** |  |  |  |
| Rationale | 3 | Describe the rationale for the review in the context of what is already known*, including mention of why a network meta-analysis has been conducted.* | 2 |
| Objectives | 4 | Provide an explicit statement of questions being addressed, with reference to participants, interventions, comparisons, outcomes, and study design (PICOS). | 3 |
|  |  |  |  |
| **METHODS** |  |  |  |
| Protocol and registration | 5 | Indicate whether a review protocol exists and if and where it can be accessed (e.g., Web address); and, if available, provide registration information, including registration number. | 3 |
| Eligibility criteria | 6 | Specify study characteristics (e.g., PICOS, length of follow-up) and report characteristics (e.g., years considered, language, publication status) used as criteria for eligibility, giving rationale. *Clearly describe eligible treatments included in the treatment network, and note whether any have been clustered or merged into the same node (with justification).* | 3 |
| Information sources | 7 | Describe all information sources (e.g., databases with dates of coverage, contact with study authors to identify additional studies) in the search and date last searched. | 3 |
| Search | 8 | Present full electronic search strategy for at least one database, including any limits used, such that it could be repeated. | 3，Supplementary  Table S2 |
| Study selection | 9 | State the process for selecting studies (i.e., screening, eligibility, included in systematic review, and, if applicable, included in the meta-analysis). | 3-4，Figure 1 |
| Data collection process | 10 | Describe method of data extraction from reports (e.g., piloted forms, independently, in duplicate) and any processes for obtaining and confirming data from investigators. | 4 |
| Data items | 11 | List and define all variables for which data were sought (e.g., PICOS, funding sources) and any assumptions and simplifications made. | 4 |
| Geometry of the network | 12 | Describe methods used to explore the geometry of the treatment network under study and potential biases related to it. This should include how the evidence base has been graphically summarized for presentation, and what characteristics were compiled and used to describe the evidence base to readers. | 4-5 |
| Risk of bias within individual studies | 13 | Describe methods used for assessing risk of bias of individual studies (including specification of whether this was done at the study or outcome level), and how this information is to be used in any data synthesis. | 4-5 |
| Summary measures | 14 | State the principal summary measures (e.g., risk ratio, difference in means). *Also describe the use of additional summary measures assessed, such as treatment rankings and surface under the cumulative ranking curve (SUCRA) values, as well as modified approaches used to present summary findings from meta-analyses.* | 5 |
| Planned methods of analysis | 15 | Describe the methods of handling data and combining results of studies for each network meta-analysis. | 5 |
| Assessment of Inconsistency | 16 | Describe the statistical methods used to evaluate the agreement of direct and indirect evidence in the treatment network(s) studied. Describe efforts taken to address its presence when found. | 5 |
| Risk of bias across studies | 17 | Specify any assessment of risk of bias that may affect the cumulative evidence (e.g., publication bias, selective reporting within studies). | 5-6 |
| Additional analyses | 18 | Describe methods of additional analyses if done, indicating which were pre-specified. This may include, but not be limited to, the following:   - Sensitivity or subgroup analyses; - Meta-regression analyses; - *Alternative formulations of the treatment network* | 6 |
| **RESULTS†** |  |  |  |
| Study selection | 19 | Give numbers of studies screened, assessed for eligibility, and included in the review, with reasons for exclusions at each stage, ideally with a flow diagram. | 6-7，Figrue 1 |
| Study characteristics | 20 | For each study, present characteristics for which data were extracted (e.g., study size, PICOS, follow-up period) and provide the citations. | 8-10,Table 1 |
| Summary of network geometry | 21 | Provide a brief overview of characteristics of the treatment network. This may include commentary on the abundance of trials and randomized patients for the different interventions and pairwise comparisons in the network, gaps of evidence in the treatment network, and potential biases reflected by the network structure. | 12 |
| Risk of bias within studies | 22 | Present data on risk of bias of each study and, if available, any outcome level assessment. | 11, Figure 2 |
| Presentation of network structure | 23 | Provide a network graph of the included studies to enable visualization of the geometry of the treatment network. | 12，Figure 3 |
| Results of individual studies | 24 | For all outcomes considered (benefits or harms), present, for each study: 1) simple summary data for each intervention group, and 2) effect estimates and confidence intervals. *Modified approaches may be needed to deal with information from larger networks.* | 12-13 |
| Synthesis of results | 25 | Present results of each meta-analysis done, including confidence/credible intervals. *In larger networks, authors may focus on comparisons versus a particular comparator (e.g. placebo or standard care), with full findings presented in an appendix. League tables and forest plots may be considered to summarize pairwise comparisons.* If additional summary measures were explored (such as treatment rankings), these should also be presented. | 14-16, Figure 4-7, Supplementary Table S6 |
| **Exploration for inconsistency** | **S1** | Describe results from investigations of inconsistency. This may include such information as measures of model fit to compare consistency and inconsistency models, P values from statistical tests, or summary of inconsistency estimates from different parts of the treatment network. | **22-27, Supplementary 5, Table S5.1-S5.6** |
| **Risk of bias across studies** | **S2** | Present results of any assessment of risk of bias across studies for the evidence base being studied. | **29-38, Supplementary 7 Figure S1-S6, Supplementary 8 Figure S7-S10** |
| **Results of additional analyses** | **S3** | Give results of additional analyses, if done (e.g., sensitivity or subgroup analyses, meta-regression analyses*, alternative network geometries studied, alternative choice of prior distributions for Bayesian analyses,* and so forth). | **10-21, Supplementary S3,S4 Table S3.1-S3.6, Table S4.1-S4.6** |
|  |  |  |  |
| **DISCUSSION** |  |  |  |
| Summary of evidence | 26 | Summarize the main findings, including the strength of evidence for each main outcome; consider their relevance to key groups (e.g., healthcare providers, users, and policy-makers). | 17-19 |
| Advantages and Limitations | 27 | Discuss limitations at study and outcome level (e.g., risk of bias), and at review level (e.g., incomplete retrieval of identified research, reporting bias). *Comment on the validity of the assumptions, such as transitivity and consistency. Comment on any concerns regarding network geometry (e.g., avoidance of certain comparisons).* | 19 |
| Conclusions | 28 | Provide a general interpretation of the results in the context of other evidence, and implications for future research. | 19 |

PICOS = population, intervention, comparators, outcomes, study design.

* Text in italics indicate S wording specific to reporting of network meta-analyses that has been added to guidance from the PRISMA statement.

† Authors may wish to plan for use of appendices to present all relevant information in full detail for items in this section.

| **Table S2.Literature Search Strategy** | |
| --- | --- |
| **Pubmed** | 1:(((((Heart Failures, Systolic[Title/Abstract]) OR (Systolic Heart Failures[Title/Abstract])) OR (Heart Failure, Reduced Ejection Fraction[Title/Abstract])) OR (Systolic Heart Failure[Title/Abstract])) OR ("Heart Failure, Diastolic"[Mesh])) AND (("Diabetes Mellitus, Type 2"[Mesh]) OR (((((((((((((((((((((((((((((((Diabetes Mellitus, Stable[Title/Abstract]) OR (Stable Diabetes Mellitus[Title/Abstract])) OR (Diabetes Mellitus, Noninsulin Dependent[Title/Abstract])) OR (Diabetes Mellitus, Adult-Onset[Title/Abstract])) OR (Adult-Onset Diabetes Mellitus[Title/Abstract])) OR (Diabetes Mellitus, Adult Onset[Title/Abstract])) OR (Diabetes Mellitus, Ketosis-Resistant[Title/Abstract])) OR (Diabetes Mellitus, Ketosis Resistant[Title/Abstract])) OR (Ketosis-Resistant Diabetes Mellitus[Title/Abstract])) OR (Diabetes Mellitus, Non Insulin Dependent[Title/Abstract])) OR (Diabetes Mellitus, Non-Insulin-Dependent[Title/Abstract])) OR (Non-Insulin-Dependent Diabetes Mellitus[Title/Abstract])) OR (Diabetes Mellitus, Type II[Title/Abstract])) OR (NIDDM[Title/Abstract])) OR (Diabetes Mellitus, Maturity-Onset[Title/Abstract])) OR (Diabetes Mellitus, Maturity Onset[Title/Abstract])) OR (Maturity-Onset Diabetes Mellitus[Title/Abstract])) OR (Maturity Onset Diabetes Mellitus[Title/Abstract])) OR (MODY[Title/Abstract])) OR (Diabetes Mellitus, Slow-Onset[Title/Abstract])) OR (Diabetes Mellitus, Slow Onset[Title/Abstract])) OR (Slow-Onset Diabetes Mellitus[Title/Abstract])) OR (Type 2 Diabetes Mellitus[Title/Abstract])) OR (Noninsulin-Dependent Diabetes Mellitus[Title/Abstract])) OR (Noninsulin Dependent Diabetes Mellitus[Title/Abstract])) OR (Maturity-Onset Diabetes[Title/Abstract])) OR (Diabetes, Maturity-Onset[Title/Abstract])) OR (Maturity Onset Diabetes[Title/Abstract])) OR (Type 2 Diabetes[Title/Abstract])) OR (Diabetes, Type 2[Title/Abstract])) OR (Diabetes Mellitus, Noninsulin-Dependent[Title/Abstract]))) 800  2:("dapagliflozin" [Supplementary Concept]) OR (((((((((2S,3R,4R,5S,6R)-2-(4-chloro-3-(4-ethoxybenzyl)phenyl)-6- (Hydroxymethyl)tetrahydro-2H-pyran-3,4,5-triol[Title/Abstract])) OR (2-(3-(4-ethoxybenzyl)-4-chlorophenyl)-6-hydroxymethyltetrahydro-2H-pyran-3,4,5-triol[Title/Abstract])) OR (BMS 512148[Title/Abstract])) OR (BMS-512148[Title/Abstract])) OR (BMS512148[Title/Abstract])) OR (Farxiga[Title/Abstract]) OR (Forxiga[Title/Abstract]))) 2166  3:("empagliflozin" [Supplementary Concept]) OR ((((((1-chloro-4-(glucopyranos-1-yl)-2-(4-(tetrahydrofuran-3-yloxy)benzyl)benzene[Title/Abstract]) OR (BI 10773[Title/Abstract])) OR (BI-10773[Title/Abstract])) OR (BI10773[Title/Abstract])) OR (Jardiance[Title/Abstract]))) 2083 4:("(2S,3R,4R,5S,6R)-2-(4-chloro-3-(4-ethoxybenzyl)phenyl)-6-(methylthio)tetrahydro-2H-pyran-3,4,5-triol" [Supplementary Concept]) OR ((((sotagliflozin[Title/Abstract]) OR (LX4211[Title/Abstract])) OR (LX-4211[Title/Abstract]))) 295  5:("Canagliflozin"[Mesh]) OR ((((Invokana[Title/Abstract]) OR (Canagliflozin, Anhydrous[Title/Abstract])) OR (1-(Glucopyranosyl)-4-methyl-3-(5-(4-fluorophenyl)-2-thienylmethyl)benzene - T777973[Title/Abstract])) OR (Canagliflozin Hemihydrate[Title/Abstract])) 1243  6:("Vildagliptin"[Mesh]) OR (((((2S)-(((3-Hydroxyadamantan-1-yl)amino)acetyl)pyrrolidine-2-carbonitrile[Title/Abstract])) OR (NVP-LAF237[Title/Abstract])) OR (NVP LAF237[Title/Abstract]) OR (Galvus[Title/Abstract])) 827  7:("Liraglutide"[Mesh]) OR (((((NN 2211[Title/Abstract]) OR (NN-2211[Title/Abstract])) OR (NN2211[Title/Abstract])) OR (Victoza[Title/Abstract])) OR (Saxenda[Title/Abstract])) 3089  8:(((((("Canagliflozin"[Mesh]) OR ((((Invokana[Title/Abstract]) OR (Canagliflozin, Anhydrous[Title/Abstract])) OR (1-(Glucopyranosyl)-4-methyl-3-(5-(4-fluorophenyl)-2-thienylmethyl)benzene - T777973[Title/Abstract])) OR (Canagliflozin Hemihydrate[Title/Abstract]))) OR (("Vildagliptin"[Mesh]) OR (((((2S)-(((3-Hydroxyadamantan-1-yl)amino)acetyl)pyrrolidine-2-carbonitrile[Title/Abstract])) OR (NVP-LAF237[Title/Abstract])) OR (NVP LAF237[Title/Abstract]) OR (Galvus[Title/Abstract])))) OR (("Liraglutide"[Mesh]) OR (((((NN 2211[Title/Abstract]) OR (NN-2211[Title/Abstract])) OR (NN2211[Title/Abstract])) OR (Victoza[Title/Abstract])) OR (Saxenda[Title/Abstract])))) OR (("dapagliflozin" [Supplementary Concept]) OR (((((((((2S,3R,4R,5S,6R)-2-(4-chloro-3-(4-ethoxybenzyl)phenyl)-6- (Hydroxymethyl)tetrahydro-2H-pyran-3,4,5-triol[Title/Abstract])) OR (2-(3-(4-ethoxybenzyl)-4-chlorophenyl)-6-hydroxymethyltetrahydro-2H-pyran-3,4,5-triol[Title/Abstract])) OR (BMS 512148[Title/Abstract])) OR (BMS-512148[Title/Abstract])) OR (BMS512148[Title/Abstract])) OR (Farxiga[Title/Abstract]) OR (Forxiga[Title/Abstract]))))) OR (("empagliflozin" [Supplementary Concept]) OR ((((((1-chloro-4-(glucopyranos-1-yl)-2-(4-(tetrahydrofuran-3-yloxy)benzyl)benzene[Title/Abstract]) OR (BI 10773[Title/Abstract])) OR (BI-10773[Title/Abstract])) OR (BI10773[Title/Abstract])) OR (Jardiance[Title/Abstract]))))) OR (("(2S,3R,4R,5S,6R)-2-(4-chloro-3-(4-ethoxybenzyl)phenyl)-6-(methylthio)tetrahydro-2H-pyran-3,4,5-triol" [Supplementary Concept]) OR ((((sotagliflozin[Title/Abstract]) OR (LX4211[Title/Abstract])) OR (LX-4211[Title/Abstract])))) 8879  9:(Randomized controlled trial[Publication Type] OR Randomized[Title/Abstract] OR Placebo[Title/Abstract])1142962  10:(((((((Heart Failures, Systolic[Title/Abstract]) OR (Systolic Heart Failures[Title/Abstract])) OR (Heart Failure, Reduced Ejection Fraction[Title/Abstract])) OR (Systolic Heart Failure[Title/Abstract])) OR ("Heart Failure, Diastolic"[Mesh])) AND (("Diabetes Mellitus, Type 2"[Mesh]) OR (((((((((((((((((((((((((((((((Diabetes Mellitus, Stable[Title/Abstract]) OR (Stable Diabetes Mellitus[Title/Abstract])) OR (Diabetes Mellitus, Noninsulin Dependent[Title/Abstract])) OR (Diabetes Mellitus, Adult-Onset[Title/Abstract])) OR (Adult-Onset Diabetes Mellitus[Title/Abstract])) OR (Diabetes Mellitus, Adult Onset[Title/Abstract])) OR (Diabetes Mellitus, Ketosis-Resistant[Title/Abstract])) OR (Diabetes Mellitus, Ketosis Resistant[Title/Abstract])) OR (Ketosis-Resistant Diabetes Mellitus[Title/Abstract])) OR (Diabetes Mellitus, Non Insulin Dependent[Title/Abstract])) OR (Diabetes Mellitus, Non-Insulin-Dependent[Title/Abstract])) OR (Non-Insulin-Dependent Diabetes Mellitus[Title/Abstract])) OR (Diabetes Mellitus, Type II[Title/Abstract])) OR (NIDDM[Title/Abstract])) OR (Diabetes Mellitus, Maturity-Onset[Title/Abstract])) OR (Diabetes Mellitus, Maturity Onset[Title/Abstract])) OR (Maturity-Onset Diabetes Mellitus[Title/Abstract])) OR (Maturity Onset Diabetes Mellitus[Title/Abstract])) OR (MODY[Title/Abstract])) OR (Diabetes Mellitus, Slow-Onset[Title/Abstract])) OR (Diabetes Mellitus, Slow Onset[Title/Abstract])) OR (Slow-Onset Diabetes Mellitus[Title/Abstract])) OR (Type 2 Diabetes Mellitus[Title/Abstract])) OR (Noninsulin-Dependent Diabetes Mellitus[Title/Abstract])) OR (Noninsulin Dependent Diabetes Mellitus[Title/Abstract])) OR (Maturity-Onset Diabetes[Title/Abstract])) OR (Diabetes, Maturity-Onset[Title/Abstract])) OR (Maturity Onset Diabetes[Title/Abstract])) OR (Type 2 Diabetes[Title/Abstract])) OR (Diabetes, Type 2[Title/Abstract])) OR (Diabetes Mellitus, Noninsulin-Dependent[Title/Abstract])))) AND ((((((("Canagliflozin"[Mesh]) OR ((((Invokana[Title/Abstract]) OR (Canagliflozin, Anhydrous[Title/Abstract])) OR (1-(Glucopyranosyl)-4-methyl-3-(5-(4-fluorophenyl)-2-thienylmethyl)benzene - T777973[Title/Abstract])) OR (Canagliflozin Hemihydrate[Title/Abstract]))) OR (("Vildagliptin"[Mesh]) OR (((((2S)-(((3-Hydroxyadamantan-1-yl)amino)acetyl)pyrrolidine-2-carbonitrile[Title/Abstract])) OR (NVP-LAF237[Title/Abstract])) OR (NVP LAF237[Title/Abstract]) OR (Galvus[Title/Abstract])))) OR (("Liraglutide"[Mesh]) OR (((((NN 2211[Title/Abstract]) OR (NN-2211[Title/Abstract])) OR (NN2211[Title/Abstract])) OR (Victoza[Title/Abstract])) OR (Saxenda[Title/Abstract])))) OR (("dapagliflozin" [Supplementary Concept]) OR (((((((((2S,3R,4R,5S,6R)-2-(4-chloro-3-(4-ethoxybenzyl)phenyl)-6- (Hydroxymethyl)tetrahydro-2H-pyran-3,4,5-triol[Title/Abstract])) OR (2-(3-(4-ethoxybenzyl)-4-chlorophenyl)-6-hydroxymethyltetrahydro-2H-pyran-3,4,5-triol[Title/Abstract])) OR (BMS 512148[Title/Abstract])) OR (BMS-512148[Title/Abstract])) OR (BMS512148[Title/Abstract])) OR (Farxiga[Title/Abstract]) OR (Forxiga[Title/Abstract]))))) OR (("empagliflozin" [Supplementary Concept]) OR ((((((1-chloro-4-(glucopyranos-1-yl)-2-(4-(tetrahydrofuran-3-yloxy)benzyl)benzene[Title/Abstract]) OR (BI 10773[Title/Abstract])) OR (BI-10773[Title/Abstract])) OR (BI10773[Title/Abstract])) OR (Jardiance[Title/Abstract]))))) OR (("(2S,3R,4R,5S,6R)-2-(4-chloro-3-(4-ethoxybenzyl)phenyl)-6-(methylthio)tetrahydro-2H-pyran-3,4,5-triol" [Supplementary Concept]) OR ((((sotagliflozin[Title/Abstract]) OR (LX4211[Title/Abstract])) OR (LX-4211[Title/Abstract])))))) AND (Randomized controlled trial[Publication Type] OR Randomized[Title/Abstract] OR Placebo[Title/Abstract]) 92 |
| **Cochrane** | #1 MeSH descriptor: [Heart Failure, Systolic] explode all trees 371  #2 MeSH descriptor: [Diabetes Mellitus, Type 2] explode all trees 26908  #3 (Heart Failure, Systolic):ti,ab,kw OR (Systolic Heart Failure):ti,ab,kw OR (Systolic Heart Failures):ti,ab,kw OR (Heart Failures, Systolic):ti,ab,kw OR (Heart Failure, Reduced Ejection Fraction):ti,ab,kw (Word variations have been searched) 12305  #4 #1 OR #3 12305  #5 (Diabetes Mellitus, Type 2):ti,ab,kw OR (Type 2 Diabetes Mellitus):ti,ab,kw OR (MODY):ti,ab,kw OR (NIDDM):ti,ab,kw OR (Diabetes Mellitus, Adult Onset):ti,ab,kw (Word variations have been searched) 58558  #6 (ketosis-resistant diabetes mellitus):ti,ab,kw OR (type 2 diabetes):ti,ab,kw OR (Diabetes Mellitus, Maturity-Onset):ti,ab,kw OR (Maturity Onset Diabetes Mellitus):ti,ab,kw OR (Diabetes Mellitus, Non Insulin Dependent):ti,ab,kw (Word variations have been searched) 69634  #7 (Noninsulin-Dependent Diabetes Mellitus):ti,ab,kw OR (Stable Diabetes Mellitus):ti,ab,kw OR (Diabetes Mellitus, Stable):ti,ab,kw OR (Diabetes Mellitus, Noninsulin Dependent):ti,ab,kw OR (Maturity Onset Diabetes):ti,ab,kw (Word variations have been searched) 5258  #8 (Diabetes Mellitus, Ketosis-Resistant):ti,ab,kw OR (Slow-Onset Diabetes Mellitus):ti,ab,kw OR (Diabetes Mellitus, Stable):ti,ab,kw OR (Diabetes, Type 2):ti,ab,kw OR (Diabetes Mellitus, Maturity Onset):ti,ab,kw (Word variations have been searched) 68672  #9 (Maturity-Onset Diabetes Mellitus):ti,ab,kw OR (Noninsulin Dependent Diabetes Mellitus):ti,ab,kw OR (Diabetes Mellitus, Adult-Onset):ti,ab,kw OR (Maturity-Onset Diabetes):ti,ab,kw OR (Diabetes Mellitus, Non-Insulin-Dependent):ti,ab,kw (Word variations have been searched) 23400  #10 (Non-Insulin-Dependent Diabetes Mellitus):ti,ab,kw OR (Diabetes, Maturity-Onset):ti,ab,kw OR (Diabetes Mellitus, Ketosis Resistant):ti,ab,kw OR (Adult-Onset Diabetes Mellitus):ti,ab,kw OR (Diabetes Mellitus, Type II):ti,ab,kw (Word variations have been searched) 27228  #11 (Diabetes Mellitus, Slow Onset):ti,ab,kw OR (Diabetes Mellitus, Slow-Onset):ti,ab,kw OR (Diabetes Mellitus, Noninsulin-Dependent):ti,ab,kw (Word variations have been searched) 653  #12 #2 OR #5 OR #6 OR #7 OR #8 OR #9 OR #10 OR #11 72180  #13 #4 AND #12 in Trials 1018  #14 (Farxiga):ti,ab,kw OR (Forxiga):ti,ab,kw (Word variations have been searched) 164  #15 (BMS 512148):ti,ab,kw OR (BMS-512148):ti,ab,kw OR (BMS512148):ti,ab,kw (Word variations have been searched) in Trials 43  #16 #14 OR #15 in Trials 197  #17 (BI 10773):ti,ab,kw OR (BI-10773):ti,ab,kw OR (BI10773):ti,ab,kw OR (Jardiance):ti,ab,kw (Word variations have been searched) 249  #18 (sotagliflozin):ti,ab,kw OR (LX4211):ti,ab,kw OR (LX-4211):ti,ab,kw in Trials 210  #19 MeSH descriptor: [Canagliflozin] explode all trees 401  #20 (Canagliflozin):ti,ab,kw OR (Invokana):ti,ab,kw OR (Canagliflozin Hemihydrate):ti,ab,kw OR (Canagliflozin, Anhydrous):ti,ab,kw OR (1 (Glucopyranosyl) 4 methyl 3 (5 (4 fluorophenyl) 2 thienylmethyl)benzene T777973):ti,ab,kw in Trials 862  #21 #19 OR #20 862  #22 MeSH descriptor: [Vildagliptin] explode all trees 375  #23 (Vildagliptin):ti,ab,kw OR (NVP-LAF237):ti,ab,kw OR (NVP LAF237):ti,ab,kw OR ((2S)(((3 Hydroxyadamantan 1 yl)amino)acetyl)pyrrolidine 2 carbonitrile):ti,ab,kw OR (Galvus):ti,ab,kw in Trials 1303  #24 #22 OR #23 1306  #25 MeSH descriptor: [Liraglutide] explode all trees 1025  #26 (Liraglutide):ti,ab,kw OR (Saxenda):ti,ab,kw OR (NN2211):ti,ab,kw OR (NN 2211):ti,ab,kw OR (NN-2211):ti,ab,kw in Trials 2516  #27 (Victoza):ti,ab,kw in Trials 174  #28 #25 OR #26 OR #27 2519  #29 #16 OR #17 OR #18 OR #21 OR #24 OR 28 in Trials 286531  #30 #13 AND #29 in Trials 257 |
| **Embase** | #29. #28 AND (2023:py OR 2024:py OR 2025:py) 105  #28. #7 AND #26 AND #27 293  #27. 'randomized controlled trial'/exp OR 'randomized controlled trial' 1416745  #26. #10 OR #13 OR #16 OR #19 OR #22 OR #25 42227  #25. #23 OR #24 17091  #24. '4p 004' OR '4p004' OR 'biolide' OR 'diavic' OR 'glucagon like peptide 1 [7-37] [26 (6 n hexadecanoyl gamma glutamyllysine) 34 arginine]' OR 'ligobesy' OR 'liraglutide recombinant' OR 'n26 (hexadecanoyl gamma glutamyl) glucagon like peptide 1 [7-37] [34 arginine]' OR 'nevolat' OR 'nn 2211' OR 'nn2211' OR 'nnc 90 1170' OR 'nnc 90-1170' OR 'nnc90 1170' OR 'nnc90-1170' OR 'pinsubet' OR 'plaobes' OR 'rd 12014' OR 'rd12014' OR 'saxenda' OR 'victoza' OR 'zegluxen' OR 'liraglutide':ab,ti 9985  #23. 'liraglutide'/exp OR 'liraglutide' 17070  #22. #20 OR #21 5845  #21. '1 [ (3 hydroxy 1 adamantylamino) acetyl] 2 pyrolidinecarbonitrile' OR '1 [ (3 hydroxyadamant 1 ylamino) acetyl] pyrolidine 2 carbonitrile' OR '1 [2 [ (3 hydroxy 1 adamantyl) amino] acetyl] pyrrolidine 2 carbonitrile' OR '1 [2 [ (3 hydroxyadamantan 1 yl) amino] acetyl] pyrrolidine 2 carbonitrile' OR '1 [2 [ (3 hydroxytricyclo [3.3.1.1 (3, 7)] dec 1 yl) amino] acetyl] 2 pyrrolidinecarbonitrile' OR '1 [ [(3 hydroxy 1 adamantyl) amino] acetyl] 2 pyrrolidinecarbonitrile' OR '1 [n (3 hydroxyadamantan 1 yl) glycyl] pyrrolidine 2 carbonitrile' OR '2 cyano 1 [ [(3 hydroxy 1 adamantanyl) amino] acetyl] pyrrolidine' OR '2 cyano 1 [ [(3 hydroxy 1 adamantyl) amino] acetyl] pyrrolidine' OR '2 cyano 1 [ [(3 hydroxyadamantan 1 yl) amino] acetyl] pyrrolidine' OR 'agnis' OR 'dalmevin' OR 'equa (vildagliptin)' OR 'galvus' OR 'glypvilo' OR 'jalra' OR 'laf 237' OR 'laf237' OR 'nvp laf 237' OR 'nvp laf237' OR 'saxotin' OR 'tutecvi' OR 'vidagliptin' OR 'videl' OR 'viglita' OR 'vildus' OR 'vysov' OR 'xiliarx' OR 'vildagliptin':ab,ti 3075  #20. 'vildagliptin'/exp OR 'vildagliptin' 5768  #19. #17 OR #18 7449  #18. '1 (glucopyranosyl) 4 methyl 3 [5 (4 fluorophenyl) 2 thienylmethyl] benzene' OR '1, 5 anhydro 1 c [3 [5 (4 fluorophenyl) 2 thenyl] 4 methylphenyl] glucitol' OR '1, 5 anhydro 1 c [3 [ [5 (4 fluorophenyl) 2 thienyl] methyl] 4 methylphenyl] d glucitol' OR '1, 5 anhydro 1 c [3 [ [5 (4 fluorophenyl) thiophen 2 yl] methyl] 4 methylphenyl] d glucitol' OR '2 [3 [5 (4 fluorophenyl) 2 thiophenylmethyl] 4 methylphenyl] 6 (hydroxymethyl) tetrahydro 3, 4, 5 pyrantriol' OR '2 [3 [5 (4 fluorophenyl) thiophen 2 ylmethyl] 4 methylphenyl] 6 (hydroxymethyl) tetrahydropyran 3, 4, 5 triol' OR '2 [3 [ [5 (4 fluorophenyl) 2 thiophenyl] methyl] 4 methylphenyl] 6 (hydroxymethyl) 3, 4, 5 oxanetriol' OR '2 [3 [ [5 (4 fluorophenyl) thiophen 2 yl] methyl] 4 methylphenyl] 6 (hydroxymethyl) oxane 3, 4, 5 triol' OR 'canagliflocin' OR 'canagliflozin hemihydrate' OR 'canaglu' OR 'invokana' OR 'jnj 28431754' OR 'jnj28431754' OR 'sulisent' OR 'ta 7284' OR 'ta7284' OR 'canagliflozin':ab,ti 3632  #17. 'canagliflozin'/exp OR 'canagliflozin' 7435  #16. #14 OR #15 1265  #15. '2 [4 chloro 3 (4 ethoxybenzyl) phenyl] 6 (methylthio) tetrahydro 2h pyran 3, 4, 5 triol' OR 'inpefa' OR 'lp 802034' OR 'lp802034' OR 'lx 4211' OR 'lx4211' OR 'methyl 5 [4 chloro 3 (4 ethoxybenzyl) phenyl] 1 thio beta levo xylopyranoside' OR 'methyl 5 [4 chloro 3 [ (4 ethoxyphenyl) methyl] phenyl] 1 thio beta levo xylopyranoside' OR 'sar 439954' OR 'sar439954' OR 'zynquista' OR 'sotagliflozin':ab,ti 628  #14. 'sotagliflozin'/exp OR 'sotagliflozin' 1247  #13. #11 OR #12 12838  #12. '1 chloro 4 (1 glucopyranosyl) 2 [4 (3 oxolanyloxy) benzyl] benzene' OR '1 chloro 4 (1 glucopyranosyl) 2 [4 (3 tetrahydrofuranyloxy) benzyl] benzene' OR '1 chloro 4 (1 glucopyranosyl) 2 [ [4 (3 oxolanyloxy) phenyl] methyl] benzene' OR '1 chloro 4 (1 glucopyranosyl) 2 [ [4 (3 tetrahydrofuranyloxy) phenyl] methyl] benzene' OR '1 chloro 4 (glucopyranos 1 yl) 2 [4 (oxolan 3 yloxy) benzyl] benzene' OR '1 chloro 4 (glucopyranos 1 yl) 2 [4 (tetrahydrofuran 3 yloxy) benzyl] benzene' OR '1 chloro 4 (glucopyranos 1 yl) 2 [ [4 (oxolan 3 yloxy) phenyl] methyl] benzene' OR '1 chloro 4 (glucopyranos 1 yl) 2 [ [4 (tetrahydrofuran 3 yloxy) phenyl] methyl] benzene' OR '1, 5 anhydro 1 [4 chloro 3 [4 [ (tetrahydro 3 furyl) oxy] benzyl] phenyl] glucitol' OR '1, 5 anhydro 1 [4 chloro 3 [ [4 [ (3 oxolanyl) oxy] phenyl] methyl] phenyl] glucitol' OR '1, 5 anhydro 1 [4 chloro 3 [ [4 [ (oxolan 3 yl) oxy] phenyl] methyl] phenyl] glucitol' OR '1, 5 anhydro 1 [4 chloro 3 [ [4 [ (tetrahydro 3 furanyl) oxy] phenyl] methyl] phenyl] glucitol' OR '1, 5 anhydro 1 [4 chloro 3 [ [4 [ (tetrahydrofuran 3 yl) oxy] phenyl] methyl] phenyl] glucitol' OR '1, 5 anhydro 1 c [4 chloro 3 [ [4 [ (3 oxolanyl) oxy] phenyl] methyl] phenyl] dextro glucitol' OR '1, 5 anhydro 1 c [4 chloro 3 [ [4 [ (oxolan 3 yl) oxy] phenyl] methyl] phenyl] dextro glucitol' OR '1, 5 anhydro 1 c [4 chloro 3 [ [4 [ (tetrahydro 3 furanyl) oxy] phenyl] methyl] phenyl] dextro glucitol' OR '1, 5 anhydro 1 c [4 chloro 3 [ [4 [ (tetrahydrofuran 3 yl) oxy] phenyl] methyl] phenyl] dextro glucitol' OR '2 [4 chloro 3 [4 [ (3 oxolanyl) oxy] benzyl] phenyl] 6 (hydroxymethyl) tetrahydro 2h pyran 3, 4, 5 triol' OR '2 [4 chloro 3 [4 [ (3 tetrahydrofuranyl) oxy] benzyl] phenyl] 6 (hydroxymethyl) tetrahydro 2h pyran 3, 4, 5 triol' OR '2 [4 chloro 3 [4 [ (oxolan 3 yl) oxy] benzyl] phenyl] 6 (hydroxymethyl) tetrahydro 2h pyran 3, 4, 5 triol' OR '2 [4 chloro 3 [4 [ (tetrahydrofuran 3 yl) oxy] benzyl] phenyl] 6 (hydroxymethyl) tetrahydro 2h pyran 3, 4, 5 triol' OR '2 [4 chloro 3 [ [4 (3 oxolanyl) oxyphenyl] methyl] phenyl] 6 (hydroxymethyl) oxane 3, 4, 5 triol' OR '2 [4 chloro 3 [ [4 (3 tetrahydrofuranyl) oxyphenyl] methyl] phenyl] 6 (hydroxymethyl) oxane 3, 4, 5 triol' OR '2 [4 chloro 3 [ [4 (oxolan 3 yl) oxyphenyl] methyl] phenyl] 6 (hydroxymethyl) oxane 3, 4, 5 triol' OR '2 [4 chloro 3 [ [4 (tetrahydrofuran 3 yl) oxyphenyl] methyl] phenyl] 6 (hydroxymethyl) oxane 3, 4, 5 triol' OR 'bi 10773' OR 'bi10773' OR 'ckd 398' OR 'ckd398' OR 'gibtulio' OR 'jardiance' OR 'oboravo' OR 'empagliflozin':ab,ti 7163  #11. 'empagliflozin'/exp OR 'empagliflozin' 12825  #10. #8 OR #9 12939  #9. '1 [4 chloro 3 (4 ethoxybenzyl) phenyl] 1 deoxy beta d glucopyranose' OR '1, 5 anhydro 1 c [4 chloro 3 [ (4 ethoxyphenyl) methyl] phenyl] dextro glucitol' OR '2 (3 (4 ethoxybenzyl) 4 chlorophenyl) 6 hydroxymethyltetrahydro 2h pyran 3, 4, 5 triol' OR '2 [4 chloro 3 (4 ethoxybenzyl) phenyl] 6 (hydroxymethyl) oxane 3, 4, 5 triol' OR '2 [4 chloro 3 [ (4 ethoxyphenyl) methyl] phenyl] 6 (hydroxymethyl) oxane 3, 4, 5 triol' OR 'andatang' OR 'bms 512148' OR 'bms512148' OR 'ckd 380' OR 'ckd380' OR 'dapagliflozin acetate' OR 'dapagliflozin propanediol' OR 'dapagliflozin propanediol monohydrate' OR 'dwp 16001' OR 'dwp16001' OR 'edistride' OR 'farxiga' OR 'forxiga' OR 'hgp 1602' OR 'hgp 1812' OR 'hgp1602' OR 'hgp1812' OR 'lyn 045' OR 'lyn045' OR 'oxra' OR 'dapagliflozin':ab,ti 7299  #8. 'dapagliflozin'/exp OR 'dapagliflozin' 12914  #7. #3 AND #6 1725  #6. #4 OR #5 495131  #5. 'adult onset diabetes' OR 'adult onset diabetes mellitus' OR 'diabetes mellitus type 2' OR 'diabetes mellitus type ii' OR 'diabetes mellitus, maturity onset' OR 'diabetes mellitus, non insulin dependent' OR 'diabetes mellitus, non-insulin-dependent' OR 'diabetes mellitus, type 2' OR 'diabetes mellitus, type ii' OR 'diabetes type 2' OR 'diabetes type ii' OR 'diabetes, adult onset' OR 'dm 2' OR 'insulin independent diabetes' OR 'insulin independent diabetes mellitus' OR 'ketosis resistant diabetes mellitus' OR 'maturity onset diabetes' OR 'maturity onset diabetes mellitus' OR 'niddm' OR 'niddm (non insulin dependent diabetes mellitus)' OR 'non insulin dependent (type 2) diabetes mellitus' OR 'non insulin dependent diabetes' OR 'non-insulin-dependent diabetes mellitus' OR 'noninsulin dependent (type 2) diabetes mellitus' OR 'noninsulin dependent diabetes' OR 'noninsulin dependent diabetes mellitus' OR 't2dm' OR 'tiidm' OR 'type 2 (insulin independent) diabetes' OR 'type 2 diabetes' OR 'type 2 diabetes mellitus' OR 'type ii diabetes' OR 'type ii diabetes mellitus' OR 'non insulin dependent diabetes mellitus':ab,ti 477951  #4. 'non insulin dependent diabetes mellitus'/exp OR 'non insulin dependent diabetes mellitus' 430778  #3. #1 OR #2 22757  #2. 'hf with reduced ejection fraction' OR 'hf-ref (heart failure with reduced ejection fraction)' OR 'hfref (heart failure with reduced ejection fraction)' OR 'heart failure with reduced ejection fraction':ab,ti 11696  #1. 'heart failure with reduced ejection fraction'/exp OR 'heart failure with reduced ejection fraction' 22006 |

**Supplement 3 Sensitivity Analysis**

**Table S3.1 Sensitivity Analysis Using the composite of outcome as the Outcome**

| **dropped_id** | **comparison** | **log_eff** | **log_lci** | **log_uci** | **OR** | **OR_lci** | **OR_uci** | **connected** |
| --- | --- | --- | --- | --- | --- | --- | --- | --- |
| Mats Christian Højbjerg Lassen，2024 | dapagliflozin | -0.37074823 | -0.6151078 | -0.1263887 | 0.69021769 | 0.5405826 | 0.8812723 | 1 |
| Mats Christian Højbjerg Lassen，2024 | empagliflozin | -0.37205679 | -0.6035752 | -0.1405383 | 0.6893151 | 0.546853 | 0.8688903 | 1 |
| Mats Christian Højbjerg Lassen，2024 | sotagliflozin | -0.70843435 | -0.9548958 | -0.4619729 | 0.49241454 | 0.3848523 | 0.6300394 | 1 |
| Mats Christian Højbjerg Lassen，2024 | vildagliptin | 0.93118369 | 0.1680131 | 1.694354 | 2.537511 | 1.182952 | 5.44313 | 1 |
| Stefan D. Anker，2021 | dapagliflozin | -0.29290432 | -0.4171914 | -0.1686172 | 0.74609353 | 0.6588948 | 0.8448322 | 1 |
| Stefan D. Anker，2021 | sotagliflozin | -0.70843435 | -0.9362869 | -0.4805818 | 0.49241454 | 0.392081 | 0.6184235 | 1 |
| Stefan D. Anker，2021 | vildagliptin | 0.93118369 | 0.1738181 | 1.688549 | 2.537511 | 1.189839 | 5.411624 | 1 |
| D.L. Bhatt，2020 | dapagliflozin | -0.29290432 | -0.4171914 | -0.1686172 | 0.74609353 | 0.6588948 | 0.8448322 | 1 |
| D.L. Bhatt，2020 | empagliflozin | -0.37205679 | -0.5836563 | -0.1604573 | 0.6893151 | 0.557855 | 0.8517542 | 1 |
| D.L. Bhatt，2020 | vildagliptin | 0.93118369 | 0.1738181 | 1.688549 | 2.537511 | 1.189839 | 5.411624 | 1 |
| John J.V. McMurray，2017 | dapagliflozin | -0.29290432 | -0.4171914 | -0.1686172 | 0.74609353 | 0.6588948 | 0.8448322 | 1 |
| John J.V. McMurray，2017 | empagliflozin | -0.37205679 | -0.5836563 | -0.1604573 | 0.6893151 | 0.557855 | 0.8517542 | 1 |
| John J.V. McMurray，2017 | sotagliflozin | -0.70843435 | -0.9362869 | -0.4805818 | 0.49241454 | 0.392081 | 0.6184235 | 1 |
| Eri Toda Kato，2019 | dapagliflozin | -0.26128438 | -0.3933704 | -0.1291983 | 0.7700619 | 0.6747788 | 0.8787997 | 1 |
| Eri Toda Kato，2019 | empagliflozin | -0.37205679 | -0.5836563 | -0.1604573 | 0.6893151 | 0.557855 | 0.8517542 | 1 |
| Eri Toda Kato，2019 | sotagliflozin | -0.70843435 | -0.9362869 | -0.4805818 | 0.49241454 | 0.392081 | 0.6184235 | 1 |
| Eri Toda Kato，2019 | vildagliptin | 0.93118369 | 0.1738181 | 1.688549 | 2.537511 | 1.189839 | 5.411624 | 1 |
| Mark C. Petrie，2020 | dapagliflozin | -0.33706764 | -0.6557418 | -0.0183935 | 0.71386055 | 0.5190569 | 0.9817747 | 1 |
| Mark C. Petrie，2020 | empagliflozin | -0.37205679 | -0.7605022 | 0.0163887 | 0.6893151 | 0.4674316 | 1.016524 | 1 |
| Mark C. Petrie，2020 | sotagliflozin | -0.70843435 | -1.105967 | -0.3109016 | 0.49241454 | 0.3308907 | 0.732786 | 1 |
| Mark C. Petrie，2020 | vildagliptin | 0.93118369 | 0.1067334 | 1.755634 | 2.537511 | 1.112638 | 5.787115 | 1 |

**Table S3.2 Sensitivity Analysis Using HHF as the Outcome**

| **dropped_id** | **comparison** | **log_eff** | **log_lci** | **log_uci** | **OR** | **OR_lci** | **OR_uci** | **connected** |
| --- | --- | --- | --- | --- | --- | --- | --- | --- |
| Stefan D. Anker，2021 | dapagliflozin | -0.30550054 | -0.5142025 | -0.0967986 | 0.7367545 | 0.5979773 | 0.9077389 | 1 |
| Stefan D. Anker，2021 | sotagliflozin | -0.63600102 | -0.8041019 | -0.4679002 | 0.52940528 | 0.4474896 | 0.626316 | 1 |
| Stefan D. Anker，2021 | vildagliptin | 0.03488231 | -0.6094541 | 0.6792187 | 1.0354978 | 0.5436476 | 1.972336 | 1 |
| D.L. Bhatt，2020 | dapagliflozin | -0.30550054 | -0.5142025 | -0.0967986 | 0.73675451 | 0.5979773 | 0.9077388 | 1 |
| D.L. Bhatt，2020 | empagliflozin | -0.59802883 | -0.7998028 | -0.3962548 | 0.5498945 | 0.4494176 | 0.6728352 | 1 |
| D.L. Bhatt，2020 | sotagliflozin | -0.57393364 | -0.8170717 | -0.3307956 | 0.56330524 | 0.4417233 | 0.718352 | 1 |
| D.L. Bhatt，2020 | vildagliptin | 0.03488231 | -0.609454 | 0.6792186 | 1.0354978 | 0.5436476 | 1.972336 | 1 |
| John J.V. McMurray，2017 | dapagliflozin | -0.30550054 | -0.5142025 | -0.0967986 | 0.7367545 | 0.5979773 | 0.9077389 | 1 |
| John J.V. McMurray，2017 | empagliflozin | -0.59802883 | -0.7998028 | -0.3962548 | 0.5498945 | 0.4494176 | 0.6728352 | 1 |
| John J.V. McMurray，2017 | sotagliflozin | -0.63600102 | -0.8041019 | -0.4679002 | 0.52940528 | 0.4474896 | 0.626316 | 1 |
| Eri Toda Kato，2019 | dapagliflozin | -0.2640904 | -0.5035155 | -0.0246653 | 0.76790412 | 0.6044021 | 0.9756364 | 1 |
| Eri Toda Kato，2019 | empagliflozin | -0.59802883 | -0.7998028 | -0.3962548 | 0.5498945 | 0.4494176 | 0.6728352 | 1 |
| Eri Toda Kato，2019 | sotagliflozin | -0.63600102 | -0.8041019 | -0.4679002 | 0.52940528 | 0.4474896 | 0.626316 | 1 |
| Eri Toda Kato，2019 | vildagliptin | 0.03488231 | -0.6094541 | 0.6792187 | 1.0354978 | 0.5436476 | 1.972336 | 1 |
| Mark C. Petrie，2020 | dapagliflozin | -0.43650713 | -0.8623633 | -0.010651 | 0.64628989 | 0.4221632 | 0.9894055 | 1 |
| Mark C. Petrie，2020 | empagliflozin | -0.59802883 | -0.7998028 | -0.3962548 | 0.5498945 | 0.4494176 | 0.6728352 | 1 |
| Mark C. Petrie，2020 | sotagliflozin | -0.63600102 | -0.8041019 | -0.4679002 | 0.52940528 | 0.4474896 | 0.626316 | 1 |
| Mark C. Petrie，2020 | vildagliptin | 0.03488231 | -0.6094541 | 0.6792187 | 1.0354978 | 0.5436476 | 1.972336 | 1 |

**Table S3.3 Sensitivity Analysis Using LVEF as the Outcome**

| **dropped_id** | **comparison** | **eff** | **lci** | **uci** | **connected** |
| --- | --- | --- | --- | --- | --- |
| A. Eshraghi，2025 | dapagliflozin | 1.9602451 | -0.05786 | 3.97835 | 1 |
| A. Eshraghi，2025 | empagliflozin | 0.20397629 | -1.177886 | 1.585839 | 1 |
| A. Eshraghi，2025 | albiglutide | -0.33546801 | -3.127364 | 2.456428 | 1 |
| A. Eshraghi，2025 | remogliflozin | -0.47825994 | -3.302042 | 2.345522 | 1 |
| A. Eshraghi，2025 | sotagliflozin | 0.06907027 | -2.675537 | 2.813678 | 1 |
| A. Eshraghi，2025 | vildagliptin | 0.49846197 | -2.255208 | 3.252132 | 1 |
| M. R. Afshani，2024 | dapagliflozin | 1.9599534 | -0.0528601 | 3.972767 | 1 |
| M. R. Afshani，2024 | empagliflozin | 0.25401601 | -1.125401 | 1.633433 | 1 |
| M. R. Afshani，2024 | albiglutide | -0.33546801 | -3.119695 | 2.448759 | 1 |
| M. R. Afshani，2024 | remogliflozin | -0.47825994 | -3.29446 | 2.33794 | 1 |
| M. R. Afshani，2024 | sotagliflozin | 0.06907027 | -2.667736 | 2.805876 | 1 |
| M. R. Afshani，2024 | vildagliptin | 0.49846197 | -2.247432 | 3.244356 | 1 |
| **Fahmida Ilyas，2021** | **dapagliflozin** | **4.1121181** | **3.192944** | **5.031292** | 1 |
| Fahmida Ilyas，2021 | empagliflozin | 0.27487004 | 0.0828794 | 0.4668607 | 1 |
| Fahmida Ilyas，2021 | albiglutide | -0.33546801 | -0.8700582 | 0.1991222 | 1 |
| Fahmida Ilyas，2021 | remogliflozin | -0.47825994 | -1.160057 | 0.2035376 | 1 |
| Fahmida Ilyas，2021 | sotagliflozin | 0.06907027 | -0.0857695 | 0.22391 | 1 |
| Fahmida Ilyas，2021 | vildagliptin | 0.49846197 | 0.2267982 | 0.7701257 | 1 |
| Stefan D. Anker，2021 | dapagliflozin | 1.9604072 | -0.0606581 | 3.981472 | 1 |
| Stefan D. Anker，2021 | empagliflozin | 0.17332837 | -1.215323 | 1.56198 | 1 |
| Stefan D. Anker，2021 | albiglutide | -0.33546801 | -3.131654 | 2.460717 | 1 |
| Stefan D. Anker，2021 | remogliflozin | -0.47825994 | -3.306283 | 2.349763 | 1 |
| Stefan D. Anker，2021 | sotagliflozin | 0.06907027 | -2.679901 | 2.818041 | 1 |
| Stefan D. Anker，2021 | vildagliptin | 0.49846197 | -2.259557 | 3.256481 | 1 |
| Matthew M.Y. Lee，2021 | dapagliflozin | 1.96025 | -0.0579443 | 3.978444 | 1 |
| Matthew M.Y. Lee，2021 | empagliflozin | 0.2227588 | -1.160125 | 1.605642 | 1 |
| Matthew M.Y. Lee，2021 | albiglutide | -0.33546801 | -3.127493 | 2.456557 | 1 |
| Matthew M.Y. Lee，2021 | remogliflozin | -0.47825994 | -3.30217 | 2.34565 | 1 |
| Matthew M.Y. Lee，2021 | sotagliflozin | 0.06907027 | -2.675669 | 2.813809 | 1 |
| Matthew M.Y. Lee，2021 | vildagliptin | 0.49846197 | -2.255339 | 3.252263 | 1 |
| Qianyu Fu，2023 | dapagliflozin | -0.08118756 | -0.7262776 | 0.5639026 | 1 |
| Qianyu Fu，2023 | empagliflozin | 0.27487003 | 0.0828793 | 0.4668607 | 1 |
| Qianyu Fu，2023 | albiglutide | -0.33546801 | -0.8700582 | 0.1991222 | 1 |
| Qianyu Fu，2023 | remogliflozin | -0.47825994 | -1.160057 | 0.2035376 | 1 |
| Qianyu Fu，2023 | sotagliflozin | 0.06907027 | -0.0857695 | 0.22391 | 1 |
| Qianyu Fu，2023 | vildagliptin | 0.49846197 | 0.2267982 | 0.7701257 | 1 |
| D.L. Bhatt，2020 | dapagliflozin | 1.9454244 | 0.1530705 | 3.737778 | 1 |
| D.L. Bhatt，2020 | empagliflozin | 0.2034261 | -0.8860012 | 1.292853 | 1 |
| D.L. Bhatt，2020 | albiglutide | -0.33546801 | -2.799687 | 2.128751 | 1 |
| D.L. Bhatt，2020 | remogliflozin | -0.47825994 | -2.978547 | 2.022027 | 1 |
| D.L. Bhatt，2020 | vildagliptin | 0.49846197 | -1.922362 | 2.919286 | 1 |
| John J.V. McMurray，2017 | dapagliflozin | 1.9454244 | 0.1530705 | 3.737778 | 1 |
| John J.V. McMurray，2017 | empagliflozin | 0.2034261 | -0.8860012 | 1.292853 | 1 |
| John J.V. McMurray，2017 | albiglutide | -0.33546801 | -2.799687 | 2.128751 | 1 |
| John J.V. McMurray，2017 | remogliflozin | -0.47825994 | -2.978547 | 2.022027 | 1 |
| John J.V. McMurray，2017 | sotagliflozin | 0.06907027 | -2.341441 | 2.479581 | 1 |
| John J. Lepore，2016 | dapagliflozin | 1.9454244 | 0.1530705 | 3.737778 | 1 |
| John J. Lepore，2016 | empagliflozin | 0.2034261 | -0.8860012 | 1.292853 | 1 |
| John J. Lepore，2016 | remogliflozin | -0.47825994 | -2.978547 | 2.022027 | 1 |
| John J. Lepore，2016 | sotagliflozin | 0.06907027 | -2.341441 | 2.479581 | 1 |
| John J. Lepore，2016 | vildagliptin | 0.49846197 | -1.922362 | 2.919286 | 1 |
| S. Bhushan，2023 | dapagliflozin | 1.9454244 | 0.1530705 | 3.737778 | 1 |
| S. Bhushan，2023 | empagliflozin | 0.2034261 | -0.8860012 | 1.292853 | 1 |
| S. Bhushan，2023 | albiglutide | -0.33546801 | -2.799687 | 2.128751 | 1 |
| S. Bhushan，2023 | sotagliflozin | 0.06907027 | -2.341441 | 2.479581 | 1 |
| S. Bhushan，2023 | vildagliptin | 0.49846197 | -1.922362 | 2.919286 | 1 |

**Table S3.4 Sensitivity Analysis Using NT-pro BNP as the Outcome**

| **dropped_id** | **comparison** | **eff** | **lci** | **uci** | **connected** |
| --- | --- | --- | --- | --- | --- |
| S. Bhushan，2023 | albiglutide | -0.05378396 | -0.6582476 | 0.5506797 | 1 |
| S. Bhushan，2023 | dapagliflozin | -0.16155553 | -0.4070269 | 0.0839158 | 1 |
| S. Bhushan，2023 | empagliflozin | -0.51566929 | -0.789248 | -0.2420906 | 1 |
| S. Bhushan，2023 | liraglutide | -0.42395142 | -1.025373 | 0.17747 | 1 |
| S. Bhushan，2023 | sotagliflozin | -0.12910661 | -0.4570374 | 0.1988241 | 1 |
| Fahmida Ilyas，2021 | albiglutide | -0.05378396 | -0.6756189 | 0.568051 | 1 |
| Fahmida Ilyas，2021 | dapagliflozin | -0.15566134 | -0.4379262 | 0.1266036 | 1 |
| Fahmida Ilyas，2021 | empagliflozin | -0.50463992 | -0.7910923 | -0.2181875 | 1 |
| Fahmida Ilyas，2021 | liraglutide | -0.42395142 | -1.04283 | 0.1949267 | 1 |
| Fahmida Ilyas，2021 | remogliflozin | 0.0009589 | -0.7444527 | 0.7463704 | 1 |
| Fahmida Ilyas，2021 | sotagliflozin | -0.12910661 | -0.4880509 | 0.2298377 | 1 |
| Stefan D. Anker，2021 | albiglutide | -0.05378396 | -0.5738216 | 0.4662537 | 1 |
| Stefan D. Anker，2021 | dapagliflozin | -0.13742794 | -0.2188772 | -0.0559786 | 1 |
| Stefan D. Anker，2021 | empagliflozin | -0.28556481 | -0.5651888 | -0.0059409 | 1 |
| Stefan D. Anker，2021 | liraglutide | -0.42395142 | -0.9404497 | 0.0925469 | 1 |
| Stefan D. Anker，2021 | remogliflozin | 0.0009589 | -0.6619127 | 0.6638305 | 1 |
| Stefan D. Anker，2021 | sotagliflozin | -0.12910661 | -0.2413625 | -0.0168507 | 1 |
| Matthew M.Y. Lee，2021 | albiglutide | -0.05378396 | -0.6215533 | 0.5139854 | 1 |
| Matthew M.Y. Lee，2021 | dapagliflozin | -0.15560094 | -0.3584225 | 0.0472206 | 1 |
| Matthew M.Y. Lee，2021 | empagliflozin | -0.60991832 | -0.8964127 | -0.323424 | 1 |
| Matthew M.Y. Lee，2021 | liraglutide | -0.42395142 | -0.9884807 | 0.1405779 | 1 |
| Matthew M.Y. Lee，2021 | remogliflozin | 0.0009589 | -0.6999845 | 0.7019023 | 1 |
| Matthew M.Y. Lee，2021 | sotagliflozin | -0.12910661 | -0.3831229 | 0.1249097 | 1 |
| D.L. Bhatt，2020 | albiglutide | -0.05378396 | -0.6582476 | 0.5506797 | 1 |
| D.L. Bhatt，2020 | dapagliflozin | -0.16155552 | -0.4070269 | 0.0839158 | 1 |
| D.L. Bhatt，2020 | empagliflozin | -0.51566929 | -0.789248 | -0.2420906 | 1 |
| D.L. Bhatt，2020 | liraglutide | -0.42395142 | -1.025373 | 0.17747 | 1 |
| D.L. Bhatt，2020 | remogliflozin | 0.0009589 | -0.730024 | 0.7319418 | 1 |
| Roni Nielsen，2020 | albiglutide | -0.05378396 | -0.6582476 | 0.5506797 | 1 |
| Roni Nielsen，2020 | dapagliflozin | -0.16155552 | -0.4070269 | 0.0839158 | 1 |
| Roni Nielsen，2020 | empagliflozin | -0.51566929 | -0.789248 | -0.2420906 | 1 |
| Roni Nielsen，2020 | remogliflozin | 0.0009589 | -0.730024 | 0.7319418 | 1 |
| Roni Nielsen，2020 | sotagliflozin | -0.12910661 | -0.4570374 | 0.1988242 | 1 |
| John J. Lepore，2016 | dapagliflozin | -0.16155553 | -0.4070269 | 0.0839158 | 1 |
| John J. Lepore，2016 | empagliflozin | -0.51566929 | -0.789248 | -0.2420906 | 1 |
| John J. Lepore，2016 | liraglutide | -0.42395142 | -1.025373 | 0.17747 | 1 |
| John J. Lepore，2016 | remogliflozin | 0.0009589 | -0.730024 | 0.7319418 | 1 |
| John J. Lepore，2016 | sotagliflozin | -0.12910661 | -0.4570374 | 0.1988241 | 1 |
| Mark C. Petrie，2020 | albiglutide | -0.05378396 | -0.7013464 | 0.5937784 | 1 |
| Mark C. Petrie，2020 | dapagliflozin | -0.20373113 | -0.6220732 | 0.214611 | 1 |
| Mark C. Petrie，2020 | empagliflozin | -0.4921354 | -0.7929671 | -0.1913037 | 1 |
| Mark C. Petrie，2020 | liraglutide | -0.42395142 | -1.068675 | 0.2207721 | 1 |
| Mark C. Petrie，2020 | remogliflozin | 0.0009589 | -0.7660461 | 0.7679639 | 1 |
| Mark C. Petrie，2020 | sotagliflozin | -0.12910661 | -0.5309764 | 0.2727632 | 1 |
| John J.V. McMurray，2023 | albiglutide | -0.05378396 | -0.6983692 | 0.5908013 | 1 |
| John J.V. McMurray，2023 | dapagliflozin | -0.14991258 | -0.4961298 | 0.1963047 | 1 |
| John J.V. McMurray，2023 | empagliflozin | -0.49339967 | -0.7923315 | -0.1944679 | 1 |
| John J.V. McMurray，2023 | liraglutide | -0.42395142 | -1.065685 | 0.2177818 | 1 |
| John J.V. McMurray，2023 | remogliflozin | 0.0009589 | -0.7635342 | 0.765452 | 1 |
| John J.V. McMurray，2023 | sotagliflozin | -0.12910661 | -0.5261613 | 0.267948 | 1 |

**Table S3.5 Sensitivity Analysis Using e-GFR as the Outcome**

| **dropped_id** | **comparison** | **eff** | **lci** | **uci** | **connected** |
| --- | --- | --- | --- | --- | --- |
| Massimo Iacoviello，2023 | dapagliflozin | -0.38531052 | -0.4708538 | -0.2997672 | 1 |
| Massimo Iacoviello，2023 | empagliflozin | 0.15739829 | 0.068481 | 0.2463156 | 1 |
| Massimo Iacoviello，2023 | liraglutide | 0.24046219 | -0.271917 | 0.7528414 | 1 |
| Massimo Iacoviello，2023 | romogliflozin | -0.02686912 | -0.6897742 | 0.6360359 | 1 |
| Massimo Iacoviello，2023 | sotagliflozin | -0.01371263 | -0.1258527 | 0.0984274 | 1 |
| Stefan D. Anker，2021 | dapagliflozin | -0.18949611 | -0.5706264 | 0.1916342 | 1 |
| Stefan D. Anker，2021 | empagliflozin | 0.12935782 | -0.5412577 | 0.7999733 | 1 |
| Stefan D. Anker，2021 | liraglutide | 0.24046219 | -0.4996461 | 0.9805705 | 1 |
| Stefan D. Anker，2021 | remogliflozin | -0.02686912 | -0.8781452 | 0.824407 | 1 |
| Stefan D. Anker，2021 | sotagliflozin | -0.01371263 | -0.5594262 | 0.532001 | 1 |
| Matthew M.Y. Lee，2021 | dapagliflozin | -0.18949611 | -0.5706264 | 0.1916342 | 1 |
| Matthew M.Y. Lee，2021 | empagliflozin | 0.15881406 | -0.3829732 | 0.7006013 | 1 |
| Matthew M.Y. Lee，2021 | liraglutide | 0.24046219 | -0.4996461 | 0.9805705 | 1 |
| Matthew M.Y. Lee，2021 | remogliflozin | -0.02686912 | -0.8781452 | 0.824407 | 1 |
| Matthew M.Y. Lee，2021 | sotagliflozin | -0.01371263 | -0.5594262 | 0.532001 | 1 |
| D.L. Bhatt，2020 | dapagliflozin | -0.1885725 | -0.4829234 | 0.1057784 | 1 |
| D.L. Bhatt，2020 | empagliflozin | 0.14860738 | -0.1908711 | 0.4880859 | 1 |
| D.L. Bhatt，2020 | liraglutide | 0.24046219 | -0.4156784 | 0.8966027 | 1 |
| D.L. Bhatt，2020 | remogliflozin | -0.02686912 | -0.8062478 | 0.7525095 | 1 |
| Roni Nielsen，2020 | dapagliflozin | -0.1885725 | -0.4829234 | 0.1057784 | 1 |
| Roni Nielsen，2020 | empagliflozin | 0.14860738 | -0.1908711 | 0.4880859 | 1 |
| Roni Nielsen，2020 | remogliflozin | -0.02686912 | -0.8062478 | 0.7525095 | 1 |
| Roni Nielsen，2020 | sotagliflozin | -0.01371263 | -0.4386402 | 0.4112149 | 1 |
| Mark C. Petrie，2020 | dapagliflozin | 0.00358836 | -0.053325 | 0.0605017 | 1 |
| Mark C. Petrie，2020 | empagliflozin | 0.15739829 | 0.068481 | 0.2463156 | 1 |
| Mark C. Petrie，2020 | liraglutide | 0.24046219 | -0.271917 | 0.7528414 | 1 |
| Mark C. Petrie，2020 | remogliflozin | -0.02686912 | -0.6897742 | 0.6360359 | 1 |
| Mark C. Petrie，2020 | sotagliflozin | -0.01371263 | -0.1258527 | 0.0984274 | 1 |
| S. Bhushan，2023 | dapagliflozin | -0.1885725 | -0.4829234 | 0.1057784 | 1 |
| S. Bhushan，2023 | empagliflozin | 0.14860737 | -0.1908712 | 0.4880859 | 1 |
| S. Bhushan，2023 | liraglutide | 0.24046219 | -0.4156784 | 0.8966027 | 1 |
| S. Bhushan，2023 | sotagliflozin | -0.01371263 | -0.4386402 | 0.4112149 | 1 |

**Table S3.6 Sensitivity Analysis Using HbA1c as the Outcome**

| **dropped_id** | **comparison** | **eff** | **lci** | **uci** | **connected** |
| --- | --- | --- | --- | --- | --- |
| A. Eshraghi，2025 | dapagliflozin | -5.049284 | -11.83737 | 1.7388 | 1 |
| A. Eshraghi，2025 | empagliflozin | -0.27270098 | -6.993758 | 6.448356 | 1 |
| A. Eshraghi，2025 | sotagliflozin | -0.07424331 | -9.575266 | 9.42678 | 1 |
| A. Eshraghi，2025 | vildagliptin | -5.1512719 | -14.66553 | 4.362982 | 1 |
| Stefan D. Anker，2021 | dapagliflozin | -5.0493293 | -11.83896 | 1.740305 | 1 |
| Stefan D. Anker，2021 | empagliflozin | -0.63011693 | -7.356836 | 6.096602 | 1 |
| Stefan D. Anker，2021 | sotagliflozin | -0.07424331 | -9.577483 | 9.428997 | 1 |
| Stefan D. Anker，2021 | vildagliptin | -5.1512719 | -14.66774 | 4.365196 | 1 |
| Matthew M.Y. Lee，2021 | dapagliflozin | -5.0497015 | -11.85212 | 1.752719 | 1 |
| Matthew M.Y. Lee，2021 | empagliflozin | -0.44592863 | -7.182591 | 6.290734 | 1 |
| Matthew M.Y. Lee，2021 | sotagliflozin | -0.07424331 | -9.595777 | 9.44729 | 1 |
| Matthew M.Y. Lee，2021 | vildagliptin | -5.1512719 | -14.68601 | 4.383464 | 1 |
| Qianyu Fu，2023 | dapagliflozin | -0.23101402 | -0.8730574 | 0.4110293 | 1 |
| Qianyu Fu，2023 | empagliflozin | -0.39715828 | -0.8275818 | 0.0332652 | 1 |
| Qianyu Fu，2023 | sotagliflozin | -0.07424331 | -0.7204409 | 0.5719543 | 1 |
| Qianyu Fu，2023 | vildagliptin | -5.1512719 | -5.969296 | -4.333248 | 1 |
| D.L. Bhatt，2020 | dapagliflozin | -4.9990069 | -10.52824 | 0.530226 | 1 |
| D.L. Bhatt，2020 | empagliflozin | -0.44935618 | -4.897598 | 3.998886 | 1 |
| D.L. Bhatt，2020 | vildagliptin | -5.1512719 | -12.86423 | 2.56169 | 1 |
| John J.V. McMurray，2017 | dapagliflozin | -4.9990069 | -10.52824 | 0.5302261 | 1 |
| John J.V. McMurray，2017 | empagliflozin | -0.44935618 | -4.897598 | 3.998886 | 1 |
| John J.V. McMurray，2017 | sotagliflozin | -0.07424331 | -7.770878 | 7.622392 | 1 |
| **Mark C. Petrie，2020** | **dapagliflozin** | **-10.065502** | **-12.09541** | **-8.035597** | 1 |
| Mark C. Petrie，2020 | empagliflozin | -0.39715828 | -0.8275818 | 0.0332652 | 1 |
| Mark C. Petrie，2020 | sotagliflozin | -0.07424331 | -0.7204409 | 0.5719543 | 1 |
| Mark C. Petrie，2020 | vildagliptin | -5.1512719 | -5.969296 | -4.333248 | 1 |

**Supplement 4 Meta-Regression Analysis**

**Table S4.1 Meta-Regression Analysis of the composite of outcome**

| **Intervening** | **Covariate** | **Coefficient** | **Standard Error** | **Z-statistic** | **P>z** | **[95% confidence interval]** | |
| --- | --- | --- | --- | --- | --- | --- | --- |
| Using Age as a Moderator | | | | | | | |
| _y_B | _cons | -0.0098522 | 0.1358899 | -0.07 | 0.942 | -0.2761915 | 0.2564871 |
| _y_C | cov1 | -0.0297409 | 0.0226679 | -1.31 | 0.19 | -0.0741692 | 0.0146875 |
|  | _cons | 2.325101 | 1.550201 | 1.5 | 0.134 | -0.713237 | 5.363439 |
| _y_D | _cons | -0.4651932 | 0.1377252 | -3.38 | 0.001 | -0.7351295 | -0.1952568 |
| _y_E | _cons | 1.382611 | 0.409796 | 3.37 | 0.001 | 0.5794253 | 2.185796 |
| Using Follow-Up Duration as a Moderator | | | | | | | |
| _y_B | _cons | -0.0020077 | 0.1352517 | -0.01 | 0.988 | -0.2670962 | 0.2630809 |
| _y_C | cov2 | 0.0018474 | 0.001225 | 1.51 | 0.132 | -0.0005536 | 0.0042485 |
|  | _cons | 0.1557464 | 0.1108738 | 1.4 | 0.16 | -0.0615622 | 0.373055 |
| _y_D | _cons | -0.4861802 | 0.1404642 | -3.46 | 0.001 | -0.761485 | -0.2108754 |
| _y_E | _cons | 1.182997 | 0.3925264 | 3.01 | 0.003 | 0.4136591 | 1.952334 |

**Table S4.2 Meta-Regression Analysis of HHF**

| **Intervening** | **Covariate** | **Coefficient** | **Standard Error** | **Z-statistic** | **P>z** | **[95% confidence interval]** | |
| --- | --- | --- | --- | --- | --- | --- | --- |
| Using Age as a Moderator | | | | | | | |
| _y_B | _cons | -0.3339383 | 0.1597502 | -2.09 | 0.037 | -0.647043 | -0.0208337 |
| _y_C | cov1 | -0.0862082 | 0.1246338 | -0.69 | 0.489 | -0.330486 | 0.1580696 |
|  | _cons | 5.953832 | 8.16666 | 0.73 | 0.466 | -10.05253 | 21.96019 |
| _y_D | _cons | -0.7167434 | 0.5748982 | -1.25 | 0.212 | -1.843523 | 0.4100363 |
| _y_E | _cons | 0.5575974 | 0.4669339 | 1.19 | 0.232 | -0.3575762 | 1.472771 |
| Using Follow-Up Duration as a Moderator | | | | | | | |
| _y_B | _cons | -0.2980183 | 0.1483205 | -2.01 | 0.045 | -0.5887211 | -0.0073154 |
| _y_C | cov2 | 0.001796 | 0.0025964 | 0.69 | 0.489 | -0.0032929 | 0.0068849 |
|  | _cons | 0.0916737 | 0.3269492 | 0.28 | 0.779 | -0.5491349 | 0.7324823 |
| _y_D | _cons | -0.4796711 | 0.2553423 | -1.88 | 0.06 | -0.9801328 | 0.0207907 |
| _y_E | _cons | 0.2199484 | 0.3869419 | 0.57 | 0.57 | -0.5384438 | 0.9783406 |

**Table S4.3 Meta-Regression Analysis of LVEF**

| **Intervening** | **Covariate** | **Coefficient** | **Standard Error** | **Z-statistic** | **P>z** | **[95% confidence interval]** | |
| --- | --- | --- | --- | --- | --- | --- | --- |
| Using Age as a Moderator | | | | | | | |
| _y_B | cov1 | -1.477195 | 2.334363 | -0.63 | 0.527 | -6.052463 | 3.098072 |
|  | _cons | 106.7336 | 131.0055 | 0.81 | 0.415 | -150.0325 | 363.4997 |
| _y_C | cov1 | -0.0566292 | 2.326839 | -0.02 | 0.981 | -4.61715 | 4.503891 |
|  | _cons | 3.577868 | 130.3046 | 0.03 | 0.978 | -251.8144 | 258.9702 |
| _y_D | cov1 | -0.0797648 | 2.326763 | -0.03 | 0.973 | -4.640137 | 4.480607 |
|  | _cons | 4.802302 | 130.2989 | 0.04 | 0.971 | -250.5788 | 260.1834 |
| _y_E | _cons | -0.8603184 | 20.9455 | -0.04 | 0.967 | -41.91273 | 40.1921 |
| _y_F | _cons | -0.7121671 | 32.57594 | -0.02 | 0.983 | -64.55984 | 63.13551 |
| _y_G | _cons | 0.2755729 | 16.29015 | 0.02 | 0.987 | -31.65253 | 32.20368 |
| Using Follow-Up Duration as a Moderator | | | | | | | |
| _y_B | cov2 | 0.0762185 | 3.14663 | 0.02 | 0.981 | -6.091063 | 6.2435 |
|  | _cons | 0.2510744 | 37.76225 | 0.01 | 0.995 | -73.76157 | 74.26372 |
| _y_C | cov2 | -0.0131882 | 3.146608 | 0 | 0.997 | -6.180426 | 6.15405 |
|  | _cons | 0.6350835 | 37.76096 | 0.02 | 0.987 | -73.37504 | 74.64521 |
| _y_D | cov2 | -0.0149183 | 3.146608 | 0 | 0.996 | -6.182156 | 6.15232 |
|  | _cons | 0.5144854 | 37.76073 | 0.01 | 0.989 | -73.49519 | 74.52416 |
| _y_E | _cons | -0.1424397 | 0.4354079 | -0.33 | 0.744 | -0.9958235 | 0.7109441 |
| _y_F | _cons | 0.046498 | 75.51859 | 0 | 1 | -147.9672 | 148.0602 |
| _y_G | _cons | 0.237192 | 125.8642 | 0 | 0.998 | -246.452 | 246.9264 |

**Table S4.4 Meta-Regression Analysis of NT-pro BNP**

| **Intervening** | **Covariate** | **Coefficient** | **Standard Error** | **Z-statistic** | **P>z** | **[95% confidence interval]** | |
| --- | --- | --- | --- | --- | --- | --- | --- |
| Using Age as a Moderator | | | | | | | |
| _y_B | cov1 | -0.0088552 | 2.000691 | 0 | 0.996 | -3.930138 | 3.912427 |
|  | _cons | 0.5766524 | 112.0469 | 0.01 | 0.996 | -219.0312 | 220.1845 |
| _y_C | cov1 | 0.162749 | 2.000966 | 0.08 | 0.935 | -3.759072 | 4.08457 |
|  | _cons | -11.32226 | 112.0704 | -0.1 | 0.92 | -230.9761 | 208.3316 |
| _y_D | _cons | -0.3061294 | 18.00687 | -0.02 | 0.986 | -35.59894 | 34.98668 |
| _y_E | cov1 | 0.007104 | 2.00032 | 0 | 0.997 | -3.913451 | 3.927659 |
|  | _cons | -0.3440392 | 112.0181 | 0 | 0.998 | -219.8955 | 219.2074 |
| _y_F | _cons | 0.1186779 | 18.00811 | 0.01 | 0.995 | -35.17656 | 35.41392 |
| _y_G | _cons | 0.0241332 | 28.00591 | 0 | 0.999 | -54.86644 | 54.91471 |
| Using Follow-Up Duration as a Moderator | | | | | | | |
| _y_B | cov2 | 0.0027323 | 2.29372 | 0 | 0.999 | -4.492876 | 4.49834 |
|  | _cons | -0.181911 | 27.52672 | -0.01 | 0.995 | -54.13329 | 53.76947 |
| _y_C | cov2 | -0.0033659 | 2.293719 | 0 | 0.999 | -4.498972 | 4.492241 |
|  | _cons | -0.0651605 | 27.52689 | 0 | 0.998 | -54.01688 | 53.88656 |
| _y_D | _cons | -0.3474197 | 27.52688 | -0.01 | 0.99 | -54.29912 | 53.60428 |
| _y_E | cov2 | 0.0018871 | 2.293719 | 0 | 0.999 | -4.493719 | 4.497493 |
|  | _cons | 0.0311388 | 27.52616 | 0 | 0.999 | -53.91915 | 53.98143 |
| _y_F | _cons | 0.0547422 | 0.4298561 | 0.13 | 0.899 | -0.7877603 | 0.8972447 |
| _y_G | _cons | -0.0300318 | 55.04965 | 0 | 1 | -107.9254 | 107.8653 |

**Table S 4.5 Meta-Regression Analysis of e-GFR**

| **Intervening** | **Covariate** | **Coefficient** | **Standard Error** | **Z-statistic** | **P>z** | **[95% confidence interval]** | |
| --- | --- | --- | --- | --- | --- | --- | --- |
| Using Age as a Moderator | | | | | | | |
| _y_B | cov1 | -0.0232358 | 9.593767 | 0 | 0.998 | -18.82667 | 18.7802 |
|  | _cons | 1.881848 | 633.1909 | 0 | 0.998 | -1239.15 | 1242.913 |
| _y_C | _cons | 0.4421944 | 9.602423 | 0.05 | 0.963 | -18.37821 | 19.2626 |
| _y_D | cov1 | -0.0123287 | 9.593047 | 0 | 0.999 | -18.81435 | 18.7897 |
|  | _cons | 1.003155 | 633.1411 | 0 | 0.999 | -1239.931 | 1241.937 |
| _y_E | _cons | 0.1749439 | 9.60482 | 0.02 | 0.985 | -18.65016 | 19.00005 |
| _y_F | _cons | 0.1264354 | 38.37369 | 0 | 0.997 | -75.08462 | 75.3375 |
| Using Follow-Up Duration as a Moderator | | | | | | | |
| _y_B | cov2 | 0.005924 | 0.0027549 | 2.15 | 0.032 | 0.0005245 | 0.0113234 |
|  | _cons | -0.0319421 | 0.3038385 | -0.11 | 0.916 | -0.6274546 | 0.5635704 |
| _y_C | _cons | 0.2257054 | 0.2631395 | 0.86 | 0.391 | -0.2900386 | 0.7414494 |
| _y_D | cov2 | 0.0055557 | 0.0007489 | 7.42 | 0 | 0.0040879 | 0.0070235 |
|  | _cons | -0.148035 | 0.0429971 | -3.44 | 0.001 | -0.2323077 | -0.0637622 |
| _y_E | _cons | -0.1082139 | 0.3401198 | -0.32 | 0.75 | -0.7748364 | 0.5584086 |
| _y_F | _cons | 0.0382565 | 0.062705 | 0.61 | 0.542 | -0.0846431 | 0.1611561 |

**Table S4.6 Meta-Regression Analysis of HbA1c**

| **Intervening** | **Covariate** | **Coefficient** | **Standard Error** | **Z-statistic** | **P>z** | **[95% confidence interval]** | |
| --- | --- | --- | --- | --- | --- | --- | --- |
| Using Age as a Moderator | | | | | | | |
| _y_B | cov1 | 2.47327 | 0.3662883 | 6.75 | 0 | 1.755359 | 3.191182 |
|  | _cons | -163.4376 | 24.4257 | -6.69 | 0 | -211.3111 | -115.5641 |
| _y_C | cov1 | 2.458547 | 0.2977461 | 8.26 | 0 | 1.874976 | 3.042119 |
|  | _cons | -162.0331 | 19.84634 | -8.16 | 0 | -200.9312 | -123.1349 |
| _y_D | _cons | 9.990985 | 1.191595 | 8.38 | 0 | 7.655501 | 12.32647 |
| _y_E | _cons | -12.29579 | 1.287752 | -9.55 | 0 | -14.81973 | -9.77184 |
| Using Follow-Up Duration as a Moderator | | | | | | | |
| _y_B | cov2 | -0.1990019 | 0.0208329 | -9.55 | 0 | -0.2398337 | -0.1581701 |
|  | _cons | 19.1299 | 2.001195 | 9.56 | 0 | 15.20763 | 23.05217 |
| _y_C | cov2 | -0.2048714 | 0.0207533 | -9.87 | 0 | -0.2455471 | -0.1641956 |
|  | _cons | 19.89866 | 1.990864 | 9.99 | 0 | 15.99664 | 23.80069 |
| _y_D | _cons | 12.44905 | 1.245342 | 10 | 0 | 10.00823 | 14.88988 |
| _y_E | _cons | 4.094141 | 0.949304 | 4.31 | 0 | 2.23354 | 5.954743 |

**Supplement 5 Grading of Recommendations Assessment, Development and Evaluation**

**Table S5.1 Grade of the composite of outcome**

| **Comparison** | **Number of studies** | **Within-study bias** | **Reporting bias** | **Indirectness** | **Imprecision** | **Heterogeneity** | **Incoherence** | **Confidence rating** |
| --- | --- | --- | --- | --- | --- | --- | --- | --- |
| dapagliflozin:placebo | 3 | No concerns | Low risk | No concerns | No concerns | Some concerns | Some concerns | Low |
| empagliflozin:placebo | 1 | No concerns | Low risk | No concerns | No concerns | Some concerns | Some concerns | Low |
| placebo:sotagliflozin | 1 | Some concerns | Low risk | No concerns | No concerns | Some concerns | Some concerns | Very low |
| placebo:vildagliptin | 1 | No concerns | Low risk | No concerns | No concerns | Some concerns | Some concerns | Low |
| dapagliflozin:empagliflozin | 0 | No concerns | Low risk | No concerns | Some concerns | No concerns | Some concerns | Low |
| dapagliflozin:sotagliflozin | 0 | No concerns | Low risk | No concerns | No concerns | Some concerns | Some concerns | Low |
| dapagliflozin:vildagliptin | 0 | No concerns | Low risk | No concerns | No concerns | Some concerns | Some concerns | Low |
| empagliflozin:sotagliflozin | 0 | No concerns | Low risk | No concerns | Some concerns | No concerns | Some concerns | Low |
| empagliflozin:vildagliptin | 0 | No concerns | Low risk | No concerns | No concerns | Some concerns | Some concerns | Low |
| sotagliflozin:vildagliptin | 0 | No concerns | Low risk | No concerns | No concerns | Some concerns | Some concerns | Low |

**Table S5.2 Grade of HHF**

| **Comparison** | **Number of studies** | **Within-study bias** | **Reporting bias** | **Indirectness** | **Imprecision** | **Heterogeneity** | **Incoherence** | **Confidence rating** |
| --- | --- | --- | --- | --- | --- | --- | --- | --- |
| dapagliflozin:placebo | 2 | No concerns | Low risk | No concerns | No concerns | Major concerns | Some concerns | Very low |
| empagliflozin:placebo | 1 | No concerns | Low risk | No concerns | No concerns | Some concerns | Some concerns | Low |
| placebo:sotagliflozin | 1 | Some concerns | Low risk | No concerns | No concerns | Some concerns | Some concerns | Very low |
| placebo:vildagliptin | 1 | No concerns | Low risk | No concerns | Some concerns | No concerns | Some concerns | Low |
| dapagliflozin:empagliflozin | 0 | No concerns | Low risk | No concerns | No concerns | Major concerns | Some concerns | Very low |
| dapagliflozin:sotagliflozin | 0 | No concerns | Low risk | No concerns | No concerns | Some concerns | Some concerns | Low |
| dapagliflozin:vildagliptin | 0 | No concerns | Low risk | No concerns | Some concerns | No concerns | Some concerns | Low |
| empagliflozin:sotagliflozin | 0 | No concerns | Low risk | No concerns | Some concerns | No concerns | Some concerns | Low |
| empagliflozin:vildagliptin | 0 | No concerns | Low risk | No concerns | Some concerns | No concerns | Some concerns | Low |
| sotagliflozin:vildagliptin | 0 | No concerns | Low risk | No concerns | No concerns | Some concerns | Some concerns | Low |

**Table S5.3 Grade of LVEF**

| **Comparison** | **Number of studies** | **Wihin-study bias** | **Reporting bias** | **Indirectness** | **Imprecision** | **Heterogeneity** | **Incoherence** | **Confidence rating** |
| --- | --- | --- | --- | --- | --- | --- | --- | --- |
| albiglutide:placebo | 1 | No concerns | Low risk | No concerns | Some concerns | No concerns | Some concerns | Low |
| dapagliflozin:placebo | 2 | Some concerns | Low risk | No concerns | No concerns | Some concerns | Some concerns | Very low |
| empagliflozin:placebo | 4 | No concerns | Low risk | No concerns | No concerns | Some concerns | Some concerns | Low |
| placebo:remogliflozin | 1 | No concerns | Low risk | No concerns | Major concerns | No concerns | Some concerns | Very low |
| placebo:sotagliflozin | 1 | Some concerns | Low risk | No concerns | Some concerns | No concerns | Some concerns | Very low |
| placebo:vildagliptin | 1 | No concerns | Low risk | No concerns | Major concerns | No concerns | Some concerns | Very low |
| albiglutide:dapagliflozin | 0 | No concerns | Low risk | No concerns | No concerns | Some concerns | Some concerns | Low |
| albiglutide:empagliflozin | 0 | No concerns | Low risk | No concerns | Some concerns | No concerns | Some concerns | Low |
| albiglutide:remogliflozin | 0 | No concerns | Low risk | No concerns | Some concerns | No concerns | Some concerns | Low |
| albiglutide:sotagliflozin | 0 | No concerns | Low risk | No concerns | Some concerns | No concerns | Some concerns | Low |
| albiglutide:vildagliptin | 0 | No concerns | Low risk | No concerns | Major concerns | No concerns | Some concerns | Very low |
| dapagliflozin:empagliflozin | 0 | No concerns | Low risk | No concerns | No concerns | Some concerns | Some concerns | Low |
| dapagliflozin:remogliflozin | 0 | No concerns | Low risk | No concerns | No concerns | No concerns | Some concerns | Moderate |
| dapagliflozin:sotagliflozin | 0 | Some concerns | Low risk | No concerns | No concerns | Major concerns | Some concerns | Very low |
| dapagliflozin:vildagliptin | 0 | No concerns | Low risk | No concerns | No concerns | No concerns | Some concerns | Moderate |
| empagliflozin:remogliflozin | 0 | No concerns | Low risk | No concerns | Some concerns | No concerns | Some concerns | Low |
| empagliflozin:sotagliflozin | 0 | No concerns | Low risk | No concerns | No concerns | No concerns | Some concerns | Moderate |
| empagliflozin:vildagliptin | 0 | No concerns | Low risk | No concerns | No concerns | No concerns | Some concerns | Moderate |
| remogliflozin:sotagliflozin | 0 | No concerns | Low risk | No concerns | Some concerns | No concerns | Some concerns | Low |
| remogliflozin:vildagliptin | 0 | No concerns | Low risk | No concerns | Some concerns | No concerns | Some concerns | Low |
| sotagliflozin:vildagliptin | 0 | No concerns | Low risk | No concerns | Some concerns | No concerns | Some concerns | Low |

**Table S5.4 Grade of NT-pro BNP**

| **Comparison** | **Number of studies** | **Within-stud bias** | **Reporting bias** | **Indirectness** | **Imprecision** | **Heterogeneity** | **Incoherence** | **Confidence rating** |
| --- | --- | --- | --- | --- | --- | --- | --- | --- |
| albiglutide:placebo | 1 | No concerns | Low risk | No concerns | Major concerns | No concerns | Some concerns | Very low |
| dapagliflozin:placebo | 3 | No concerns | Low risk | No concerns | No concerns | No concerns | Some concerns | Moderate |
| empagliflozin:placebo | 2 | No concerns | Low risk | No concerns | No concerns | No concerns | Some concerns | Moderate |
| liraglutide:placebo | 1 | No concerns | Low risk | No concerns | No concerns | No concerns | Some concerns | Moderate |
| placebo:remogliflozin | 1 | No concerns | Low risk | No concerns | Major concerns | No concerns | Some concerns | Very low |
| placebo:sotagliflozin | 1 | Some concerns | Low risk | No concerns | No concerns | Some concerns | Some concerns | Very low |
| albiglutide:dapagliflozin | 0 | No concerns | Low risk | No concerns | Some concerns | No concerns | Some concerns | Low |
| albiglutide:empagliflozin | 0 | No concerns | Low risk | No concerns | Some concerns | Some concerns | Some concerns | Very low |
| albiglutide:liraglutide | 0 | No concerns | Low risk | No concerns | No concerns | Some concerns | Some concerns | Low |
| albiglutide:remogliflozin | 0 | No concerns | Low risk | No concerns | Major concerns | No concerns | Some concerns | Very low |
| albiglutide:sotagliflozin | 0 | No concerns | Low risk | No concerns | Major concerns | No concerns | Some concerns | Very low |
| dapagliflozin:empagliflozin | 0 | No concerns | Low risk | No concerns | No concerns | Some concerns | Some concerns | Low |
| dapagliflozin:liraglutide | 0 | No concerns | Low risk | No concerns | No concerns | Some concerns | Some concerns | Low |
| dapagliflozin:remogliflozin | 0 | No concerns | Low risk | No concerns | Some concerns | No concerns | Some concerns | Low |
| dapagliflozin:sotagliflozin | 0 | Some concerns | Low risk | No concerns | No concerns | Major concerns | Some concerns | Very low |
| empagliflozin:liraglutide | 0 | No concerns | Low risk | No concerns | Major concerns | No concerns | Some concerns | Very low |
| empagliflozin:remogliflozin | 0 | No concerns | Low risk | No concerns | No concerns | Some concerns | Some concerns | Low |
| empagliflozin:sotagliflozin | 0 | No concerns | Low risk | No concerns | Some concerns | No concerns | Some concerns | Low |
| liraglutide:remogliflozin | 0 | No concerns | Low risk | No concerns | Some concerns | No concerns | Some concerns | Low |
| liraglutide:sotagliflozin | 0 | No concerns | Low risk | No concerns | Some concerns | Some concerns | Some concerns | Very low |
| remogliflozin:sotagliflozin | 0 | No concerns | Low risk | No concerns | Some concerns | No concerns | Some concerns | Low |

**Table S5.5 Grade of e-GFR**

| **Comparison** | **Number of studies** | **Within-study bias** | **Reporting bias** | **Indirectness** | **Imprecision** | **Heterogeneity** | **Incoherence** | **Confidence rating** |
| --- | --- | --- | --- | --- | --- | --- | --- | --- |
| dapagliflozin:placebo | 2 | No concerns | Low risk | No concerns | Some concerns | Some concerns | Some concerns | Very low |
| empagliflozin:placebo | 2 | No concerns | Low risk | No concerns | Some concerns | No concerns | Some concerns | Low |
| liraglutide:placebo | 1 | No concerns | Low risk | No concerns | No concerns | Some concerns | Some concerns | Low |
| placebo:romogliflozin | 1 | No concerns | Low risk | No concerns | Some concerns | No concerns | Some concerns | Low |
| placebo:sotagliflozin | 1 | Some concerns | Low risk | No concerns | Some concerns | No concerns | Some concerns | Very low |
| dapagliflozin:empagliflozin | 0 | No concerns | Low risk | No concerns | No concerns | No concerns | Some concerns | Moderate |
| dapagliflozin:liraglutide | 0 | No concerns | Low risk | No concerns | No concerns | No concerns | Some concerns | Moderate |
| dapagliflozin:romogliflozin | 0 | No concerns | Low risk | No concerns | Some concerns | No concerns | Some concerns | Low |
| dapagliflozin:sotagliflozin | 0 | No concerns | Low risk | No concerns | Some concerns | Some concerns | Some concerns | Very low |
| empagliflozin:liraglutide | 0 | No concerns | Low risk | No concerns | Major concerns | No concerns | Some concerns | Very low |
| empagliflozin:romogliflozin | 0 | No concerns | Low risk | No concerns | Some concerns | No concerns | Some concerns | Low |
| empagliflozin:sotagliflozin | 0 | No concerns | Low risk | No concerns | Major concerns | No concerns | Some concerns | Very low |
| liraglutide:romogliflozin | 0 | No concerns | Low risk | No concerns | Some concerns | No concerns | Some concerns | Low |
| liraglutide:sotagliflozin | 0 | No concerns | Low risk | No concerns | Some concerns | No concerns | Some concerns | Low |
| remogliflozin:sotagliflozin | 0 | No concerns | Low risk | No concerns | Major concerns | No concerns | Some concerns | Very low |

**Table S 5.6 Grade of HbA1c**

| **Comparison** | **Number of studies** | **Within-study bias** | **Reporting bias** | **Indirectness** | **Imprecision** | **Heterogeneity** | **Incoherence** | **Confidence rating** |
| --- | --- | --- | --- | --- | --- | --- | --- | --- |
| dapagliflozin:placebo | 2 | No concerns | Low risk | No concerns | No concerns | Some concerns | Some concerns | Low |
| empagliflozin:placebo | 3 | No concerns | Low risk | No concerns | Some concerns | No concerns | Some concerns | Low |
| placebo:sotagliflozin | 1 | Some concerns | Low risk | No concerns | Some concerns | No concerns | Some concerns | Very low |
| placebo:vildagliptin | 1 | No concerns | Low risk | No concerns | No concerns | Some concerns | Some concerns | Low |
| dapagliflozin:empagliflozin | 0 | No concerns | Low risk | No concerns | No concerns | Some concerns | Some concerns | Low |
| dapagliflozin:sotagliflozin | 0 | No concerns | Low risk | No concerns | No concerns | Some concerns | Some concerns | Low |
| dapagliflozin:vildagliptin | 0 | No concerns | Low risk | No concerns | Some concerns | No concerns | Some concerns | Low |
| empagliflozin:sotagliflozin | 0 | No concerns | Low risk | No concerns | Major concerns | No concerns | Some concerns | Very low |
| empagliflozin:vildagliptin | 0 | No concerns | Low risk | No concerns | No concerns | Major concerns | Some concerns | Very low |
| sotagliflozin:vildagliptin | 0 | No concerns | Low risk | No concerns | No concerns | Some concerns | Some concerns | Low |

**Supplement 6 Surface Under the Cumulative Ranking Curve**

**Table S6 SUCRA**

| **Treatment** | **SUCRA** | **PrBest** | **Mean Rank** |
| --- | --- | --- | --- |
| The Composite of Outcome of Worsening Heart Failure or Cardiovascular Death | | | |
| dapagliflozin | 56.9 | 0.1 | 2.7 |
| empagliflozin | 68.5 | 1.7 | 2.3 |
| placebo | 24.8 | 0 | 4.0 |
| sotagliflozin | 99.5 | 98.2 | 1 |
| vildagliptin | 0.2 | 0 | 5.0 |
| Hospitalization for Heart Failure | | | |
| dapagliflozin | 46.8 | 0.2 | 3.1 |
| empagliflozin | 83.5 | 38.6 | 1.7 |
| placebo | 13.6 | 0 | 4.5 |
| sotagliflozin | 89.4 | 59.6 | 1.4 |
| vildagliptin | 16.7 | 1.6 | 4.3 |
| Left Ventricular Ejection Fraction | | | |
| albiglutide | 15.5 | 0.1 | 6.1 |
| dapagliflozin | 99.4 | 96.6 | 1 |
| empagliflozin | 63.4 | 0 | 3.2 |
| placebo | 34.7 | 0 | 4.9 |
| remogliflozin | 9.5 | 0.3 | 6.4 |
| sotagliflozin | 67.7 | 3 | 2.9 |
| vildagliptin | 59.9 | 0 | 3.4 |
| N-terminal pro-B-type Natriuretic Peptide | | | |
| albiglutide | 35.2 | 2.6 | 4.9 |
| dapagliflozin | 49.4 | 0.1 | 4 |
| empagliflozin | 93.3 | 65.8 | 1.4 |
| liraglutide | 75.7 | 27.1 | 2.5 |
| placebo | 20 | 0 | 5.8 |
| remogliflozin | 32.4 | 4.2 | 5.1 |
| sotagliflozin | 44 | 0.1 | 4.4 |
| Estimated Glomerular Filtration Rate | | | |
| dapagliflozin | 24.5 | 0 | 4.8 |
| empagliflozin | 77.5 | 17.8 | 2.1 |
| liraglutide | 77.4 | 52.3 | 2.1 |
| placebo | 43.4 | 0 | 3.8 |
| remogliflozin | 43.5 | 29.8 | 3.8 |
| sotagliflozin | 33.7 | 0.1 | 4.3 |
| Glycated Hemoglobin | | | |
| dapagliflozin | 65 | 17.2 | 2.4 |
| empagliflozin | 60.3 | 12.6 | 2.6 |
| placebo | 10 | 0 | 4.6 |
| sotagliflozin | 27.9 | 3.8 | 3.9 |
| vildagliptin | 86.8 | 66.4 | 1.5 |

**Supplement 7 Funnel Plot of Network**

**
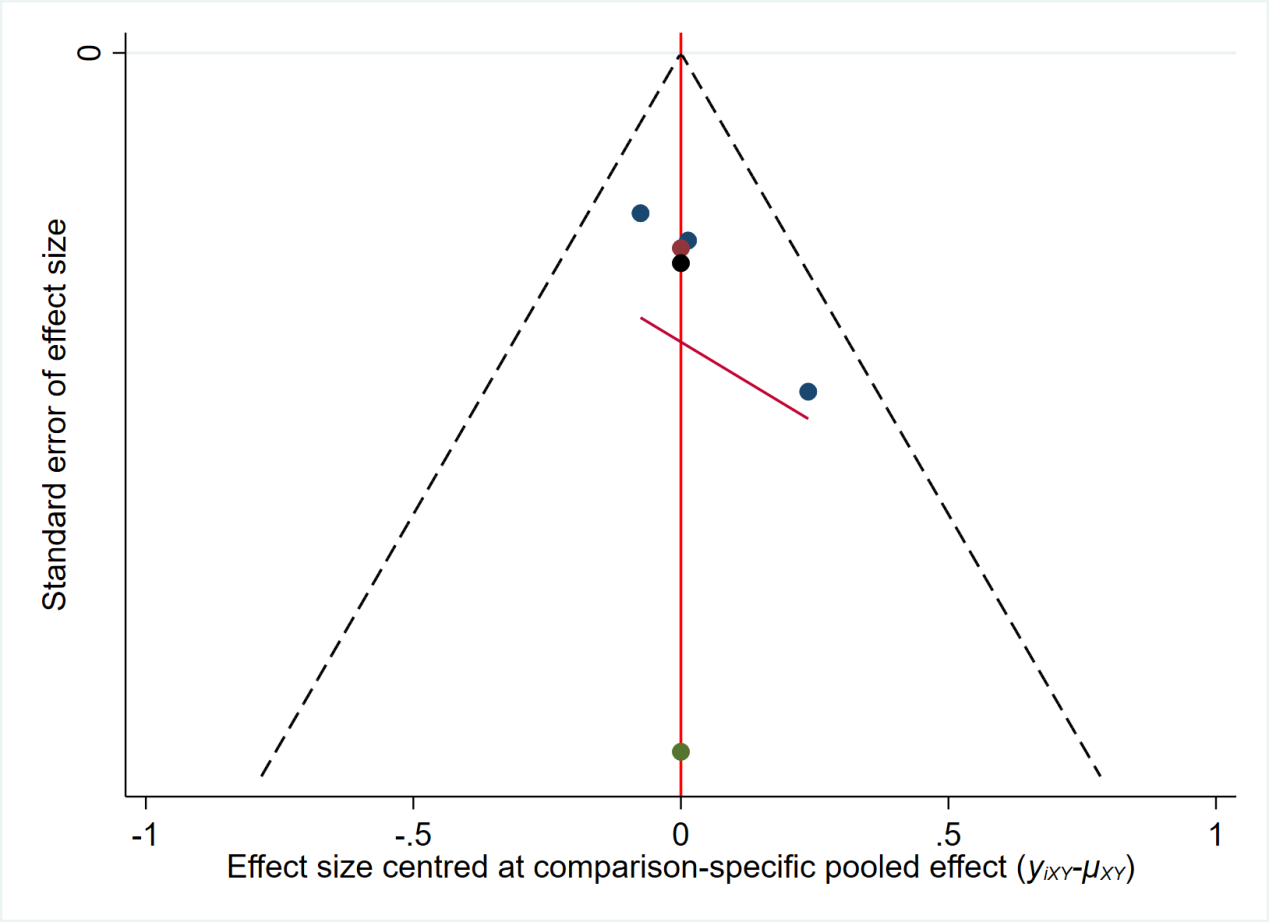
**

**Figure S1. Funnel Plot of Network Meta-analysis of the Composite of Cardiovascular Death and Heart Failure Hospitalization**

**
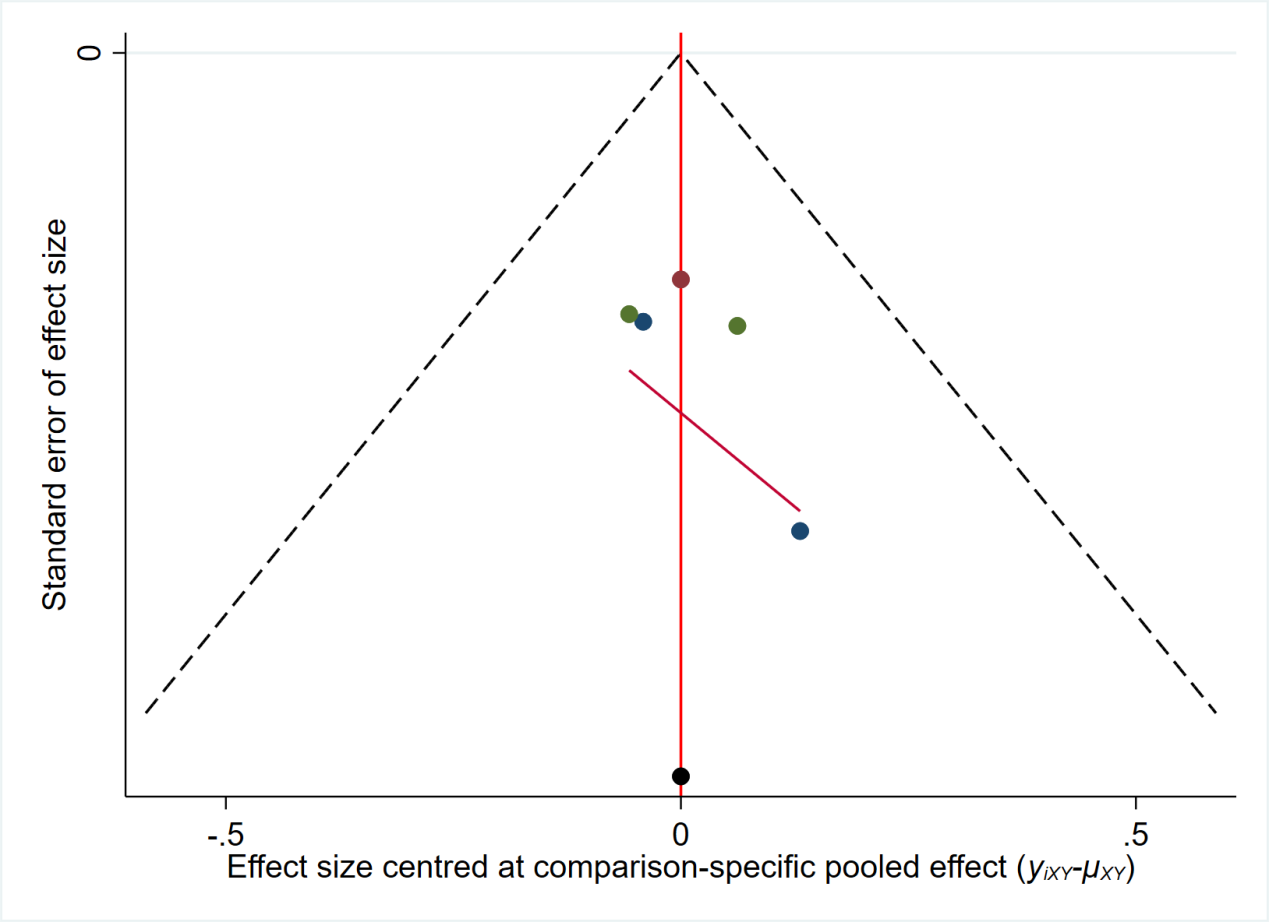
**

**Figure S2. Funnel Plot of Network Meta-analysis of Hospitalization for Heart Failure**

**
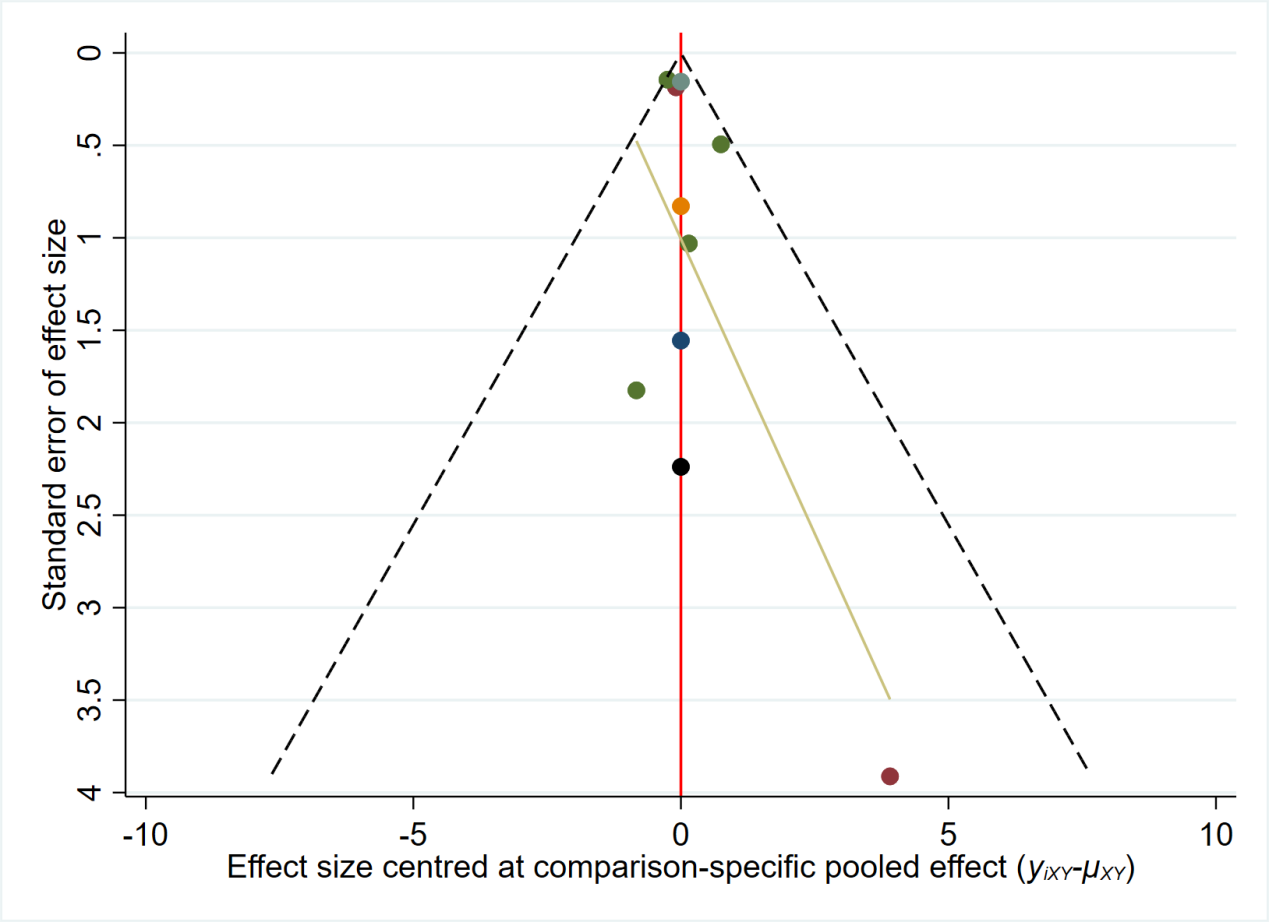
**

**Figure S3. Funnel Plot of Network Meta-analysis of Left Ventricular Ejection Fraction**

**
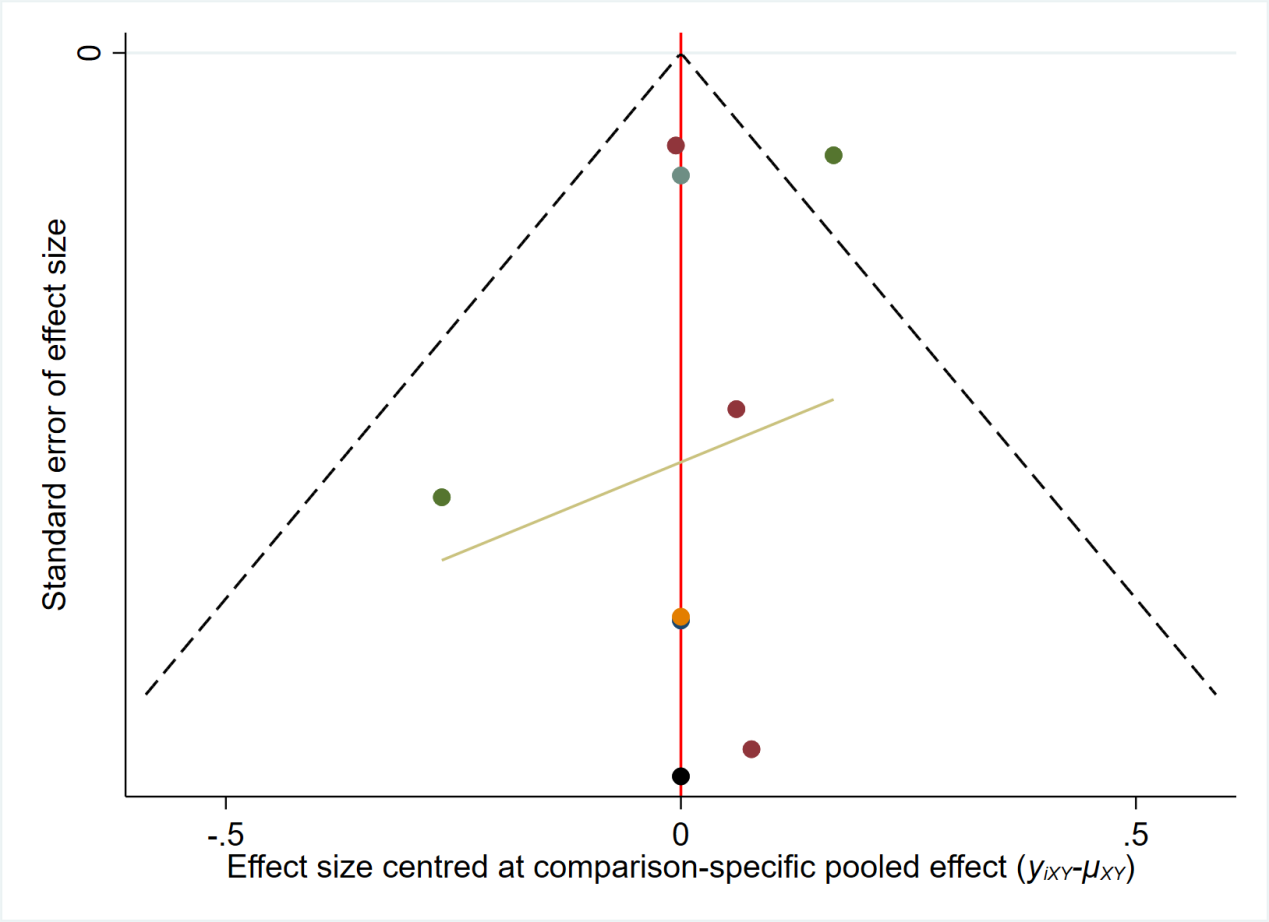
**

**Figure S4. Funnel Plot of Network Meta-analysis of NT-pro BNP**

**
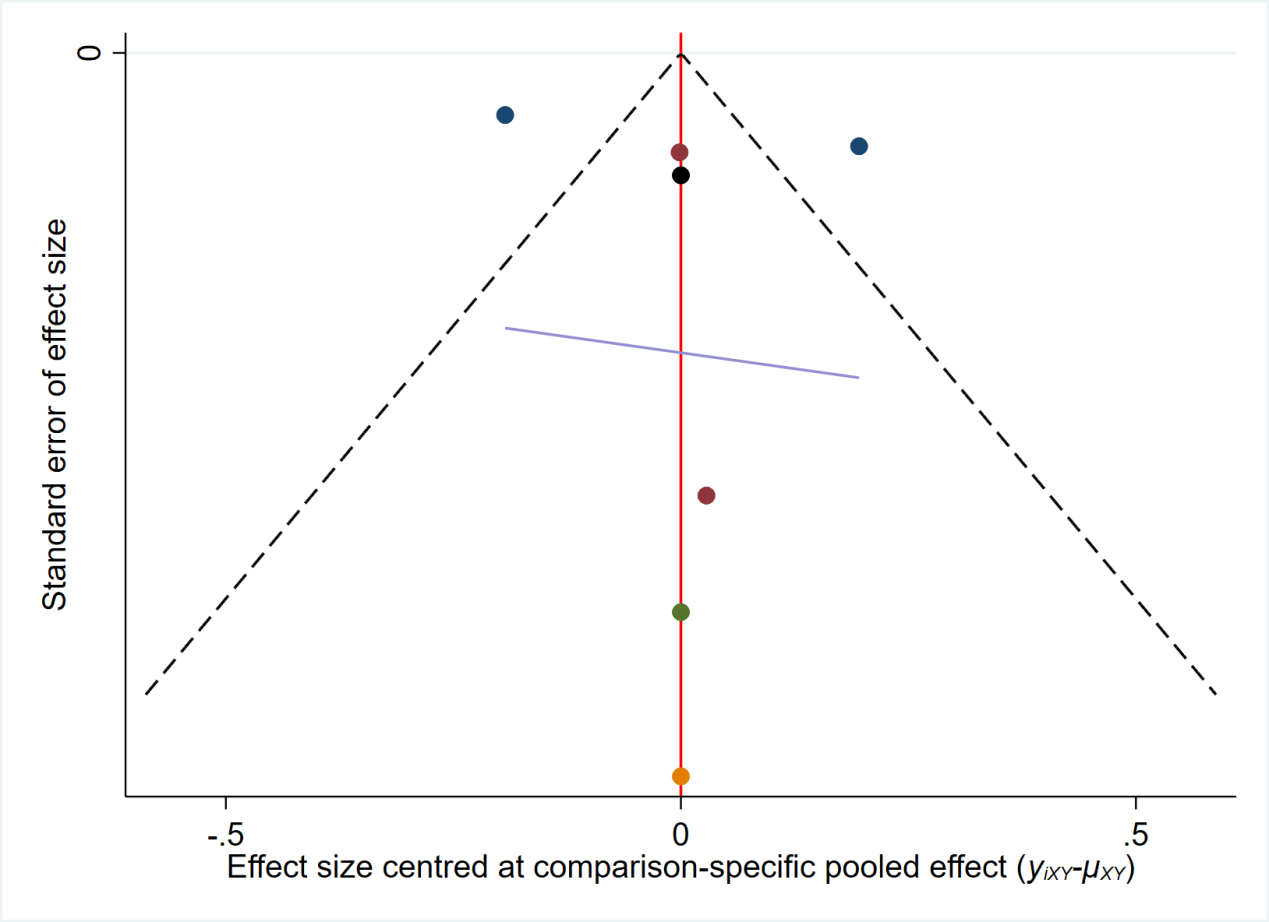
**

**Figure S5. Funnel Plot of Network Meta-analysis of e-GFR**

**
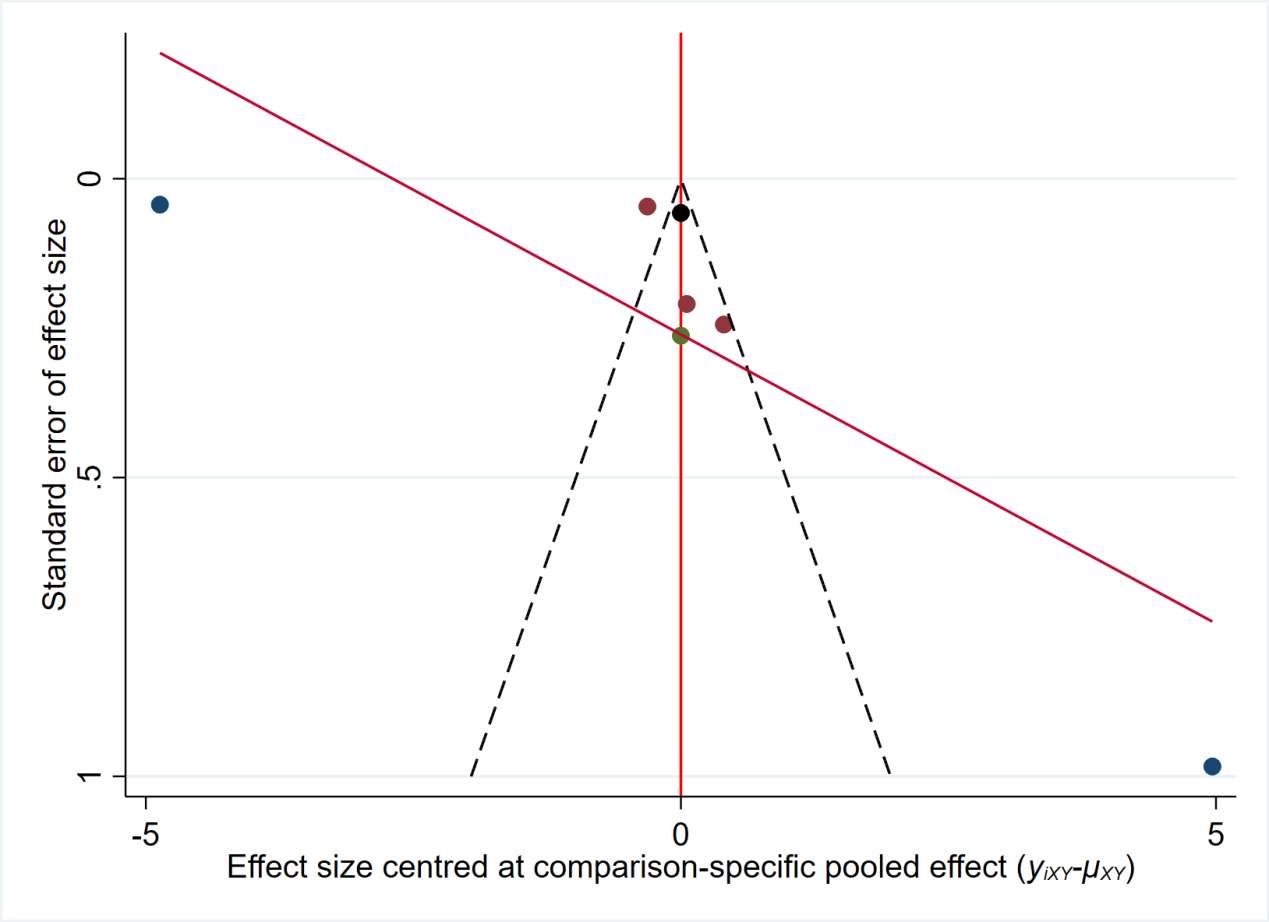
**

**Figure S6. Funnel Plot of Network Meta-analysis of HbA1c**

**Supplement 8 League table comparing therapeutic effects of hypoglycemic drugs**

**
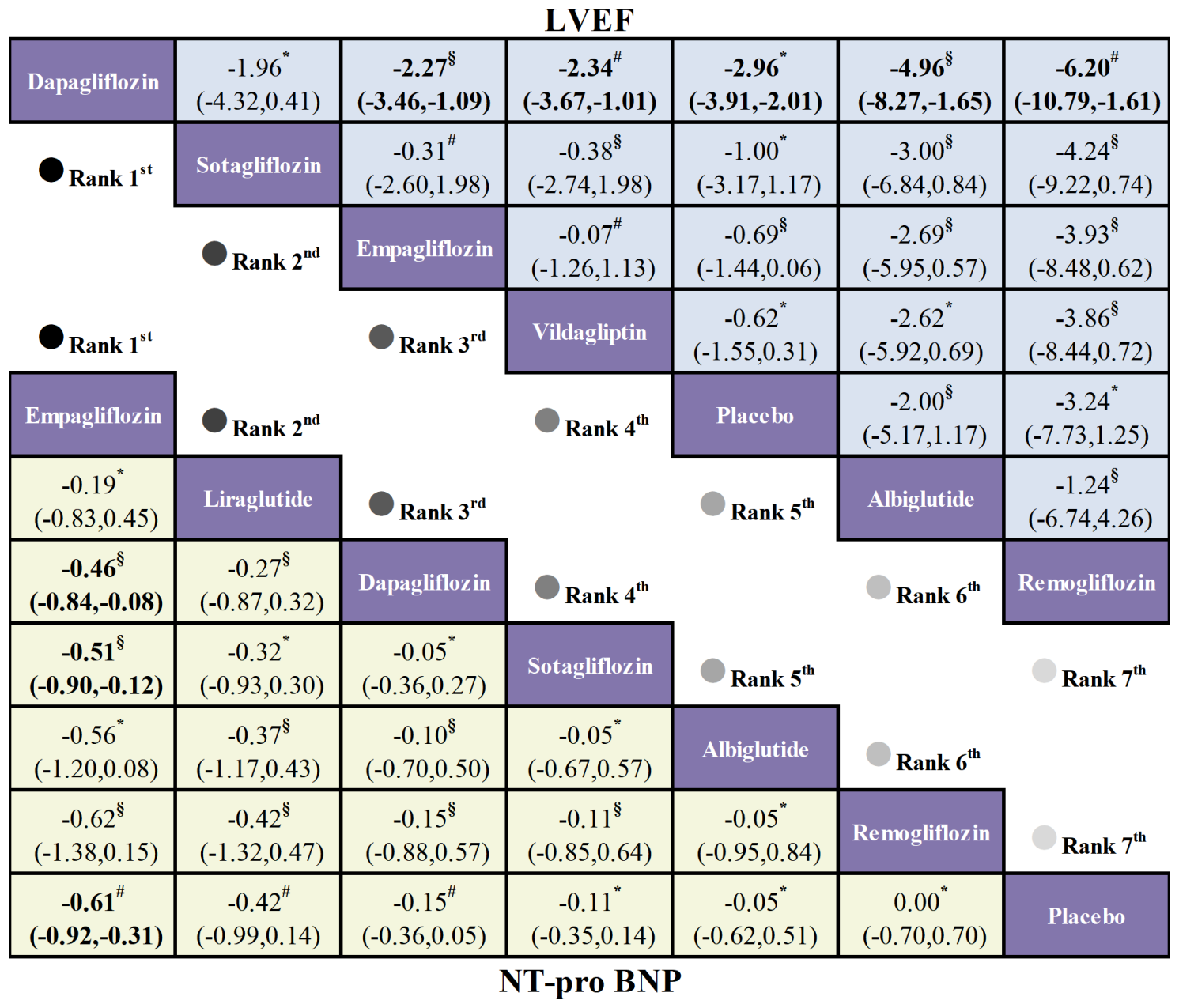
**

**Figure S7**. **The assumption of r = 0.25 for variance imputation,** **league table comparing therapeutic effects of hypoglycemic drugs on LVEF and NT-proBNP in HFrEF patients with T2DM (GRADE: Low:§ Very Low:* Moderate:#)**

**
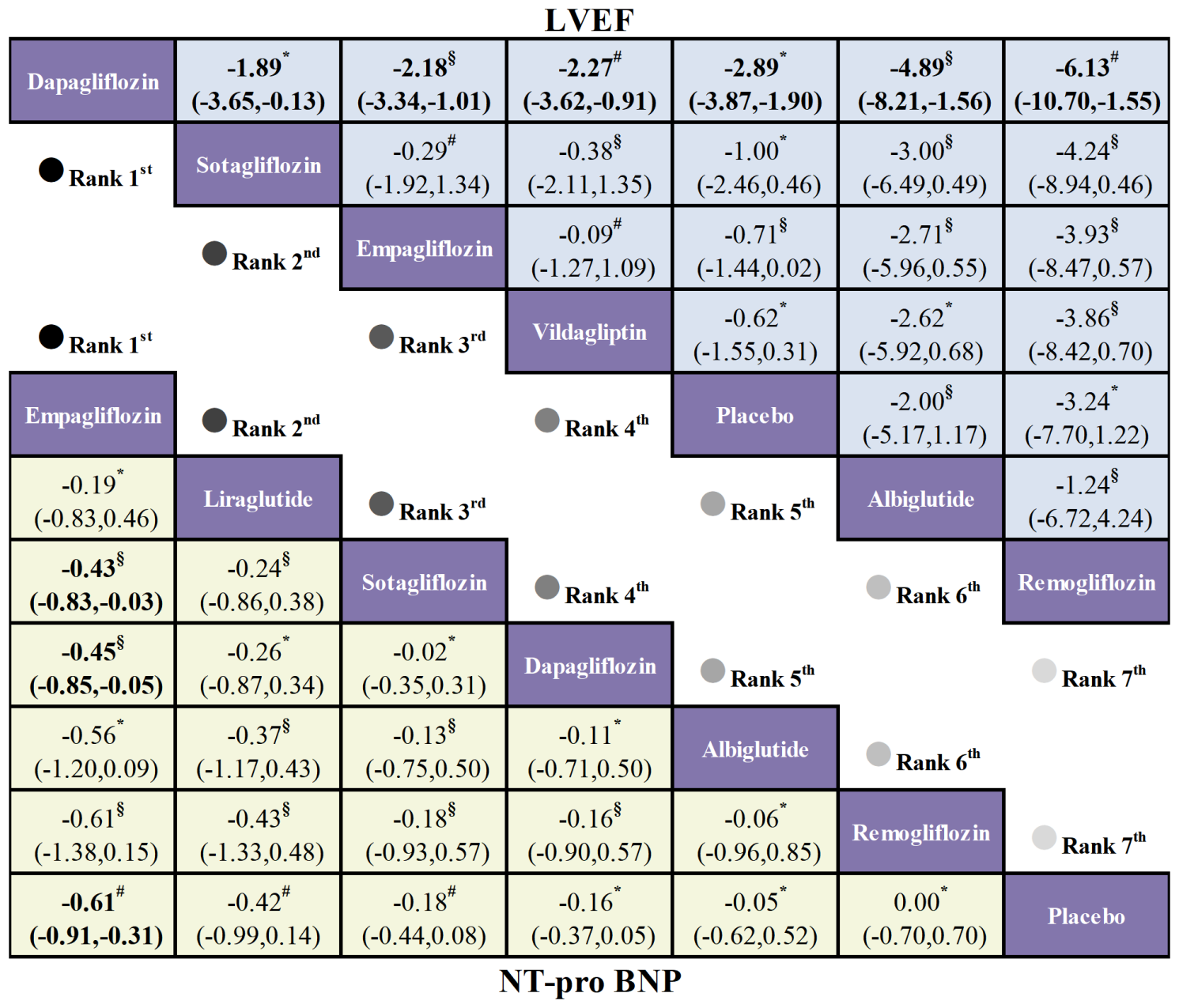
**

**Figure S8**. **The assumption of r = 0.75 for variance imputation,** **league table comparing therapeutic effects of hypoglycemic drugs on LVEF and NT-proBNP in HFrEF patients with T2DM (GRADE: Low:§ Very Low:* Moderate:#)**

**
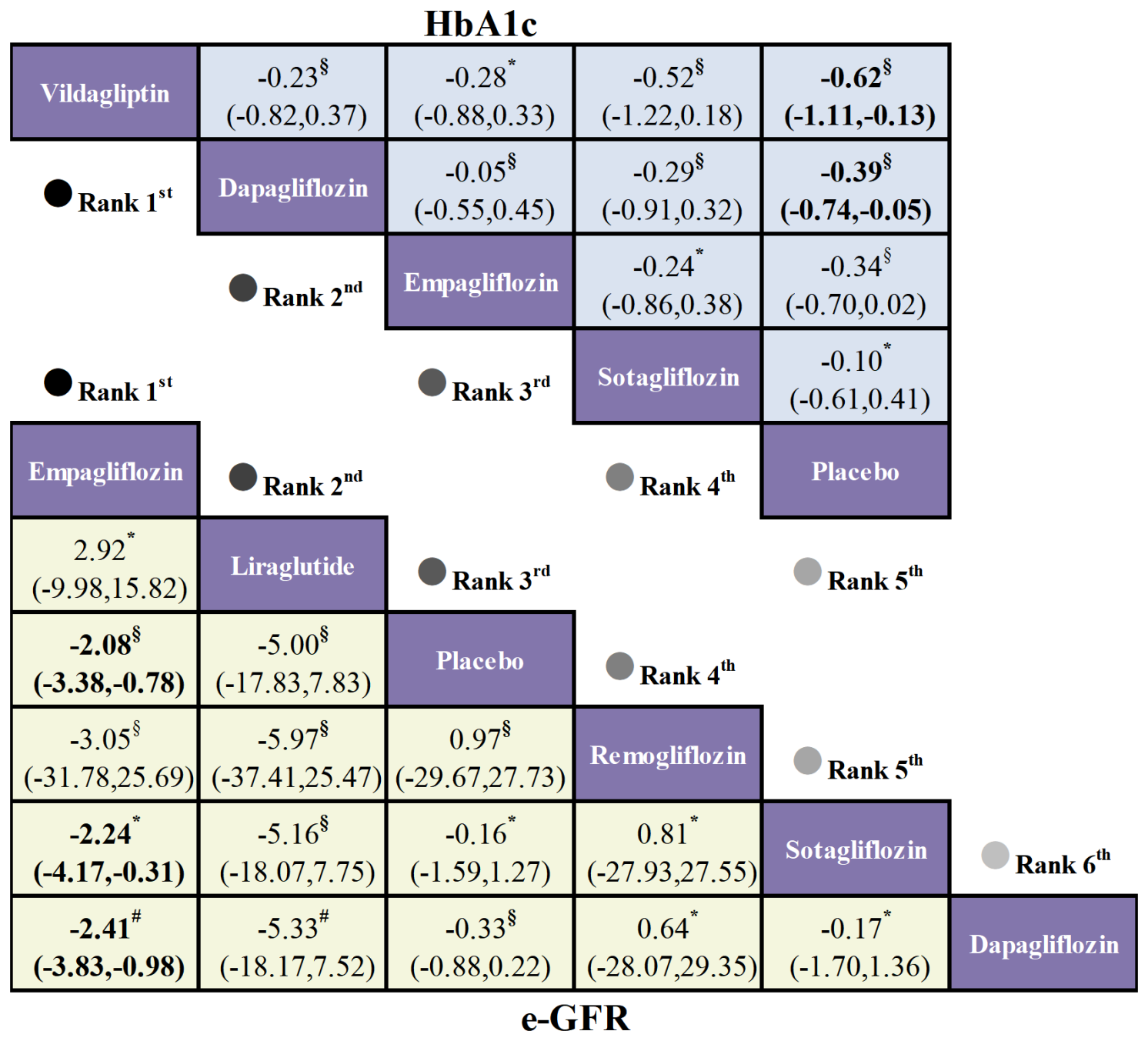
**

**Figure S9**. **The assumption of r = 0.25 for variance imputation, league table comparing the efficacy of different hypoglycemic drugs on HbA1c and e-GFR in HFrEF patients with T2DM (GRADE scoring: Low:§ Very Low:* Moderate:#)**

**
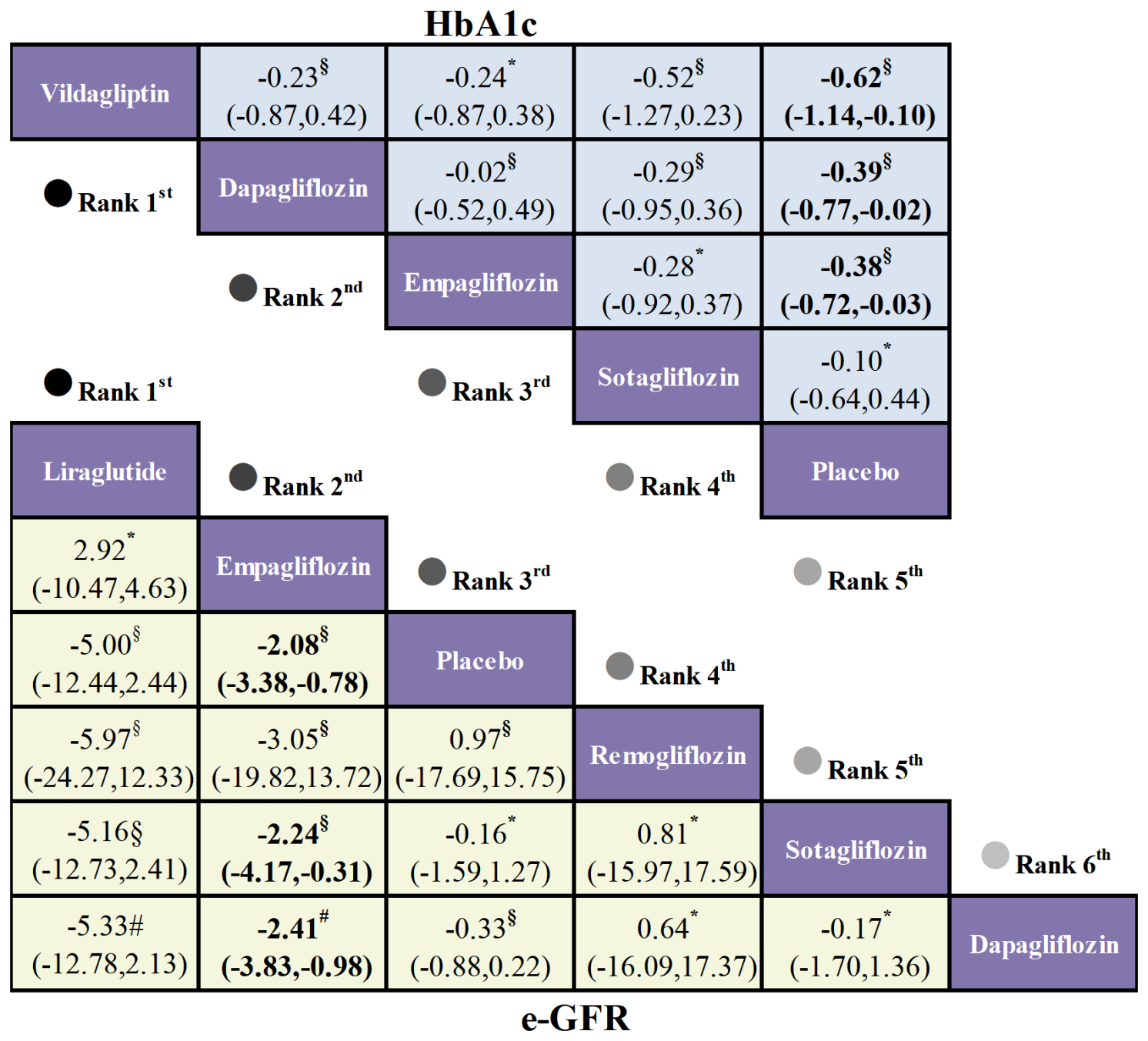
**

**Figure S10**. **The assumption of r = 0.75 for variance imputation, league table comparing the efficacy of different hypoglycemic drugs on HbA1c and e-GFR in HFrEF patients with T2DM (GRADE scoring: Low:§ Very Low:* Moderate:#)**
